# A Great late Ediacaran ice age

**DOI:** 10.1093/nsr/nwad117

**Published:** 2023-04-25

**Authors:** Ruimin Wang, Bing Shen, Xianguo Lang, Bin Wen, Ross N Mitchell, Haoran Ma, Zongjun Yin, Yongbo Peng, Yonggang Liu, Chuanming Zhou

**Affiliations:** Key Laboratory of Orogenic Belts and Crustal Evolution, Ministry of Education and School of Earth and Space Sciences, Peking University, Beijing 100871, China; Key Laboratory of Orogenic Belts and Crustal Evolution, Ministry of Education and School of Earth and Space Sciences, Peking University, Beijing 100871, China; State Key Laboratory of Oil and Gas Reservoir Geology and Exploitation, and Institute of Sedimentary Geology, Chengdu University of Technology, Chengdu 610059, China; State Key Laboratory of Geological Processes and Mineral Resources, School of Earth Sciences, China University of Geosciences, Wuhan 430074, China; State Key Laboratory of Lithospheric Evolution, Institute of Geology and Geophysics, Chinese Academy of Sciences, Beijing 100029, China; College of Earth and Planetary Sciences, University of Chinese Academy of Sciences, Beijing 100049, China; School of Earth Sciences and Engineering, Nanjing University, Nanjing 210023, China; State Key Laboratory of Palaeobiology and Stratigraphy, Nanjing Institute of Geology and Palaeontology, and Center for Excellence in Life and Paleoenvironment, Chinese Academy of Sciences, Nanjing 210008, China; School of Earth Sciences and Engineering, Nanjing University, Nanjing 210023, China; School of Physics, Peking University, Beijing 100871, China; State Key Laboratory of Palaeobiology and Stratigraphy, Nanjing Institute of Geology and Palaeontology, and Center for Excellence in Life and Paleoenvironment, Chinese Academy of Sciences, Nanjing 210008, China

**Keywords:** carbonate carbon isotopes, Shuram excursion, inertial interchange true polar wander (IITPW), hankalchough glaciation, Tarim Block

## Abstract

The emergence of the Ediacara biota soon after the Gaskiers glaciation ca. 580 million years ago (Ma) implies a possible glacial fuse for the evolution of animals. However, the timing of Ediacaran glaciation remains controversial because of poor age constraints on the ∼30 Ediacaran glacial deposits known worldwide. In addition, paleomagnetic constraints and a lack of convincing Snowball-like cap carbonates indicate that Ediacaran glaciations likely did not occur at low latitudes. Thus, reconciling the global occurrences without global glaciation remains a paradox. Here, we report that the large amplitude, globally synchronous ca. 571–562 Ma Shuram carbon isotope excursion occurs below the Ediacaran Hankalchough glacial deposit in Tarim, confirming a post-Shuram glaciation. Leveraging paleomagnetic evidence for a ∼90° reorientation of all continents due to true polar wander, and a non-Snowball condition that rules out low-latitude glaciations, we use paleogeographic reconstructions to further constrain glacial ages. Our results depict a ‘Great Ediacaran Glaciation’ occurring diachronously but continuously from ca. 580–560 Ma as different continents migrated through polar–temperate latitudes. The succession of radiation, turnover and extinction of the Ediacara biota strongly reflects glacial–deglacial dynamics.

## Teaser

A Great Ediacaran Glaciation (GEG) lasting at least 20 million years from continent to continent in mid-to-high latitudes witnessed the evolution of the Ediacara biota.

## INTRODUCTION

**Figure 1.**
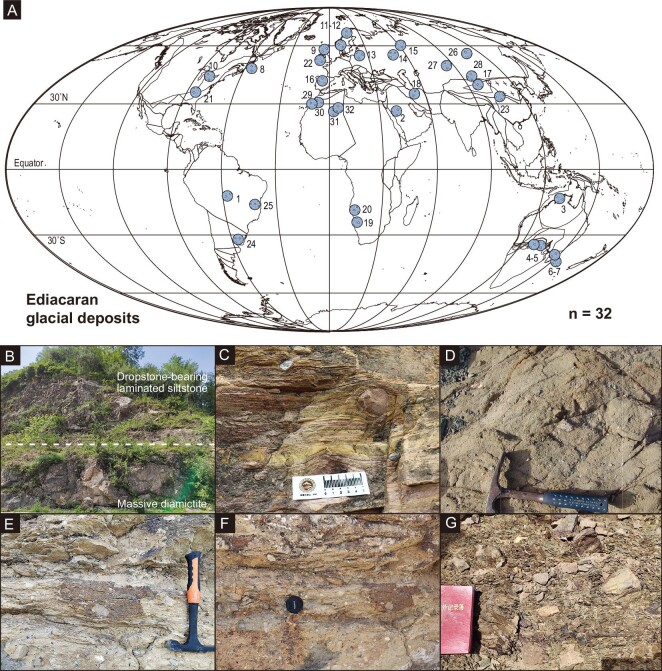
Typical Ediacaran glacial deposits. (A) Global spatial distribution of Ediacaran glacial-periglacial deposits. See compilation in Table S1 for names of each deposit. (B, C) Field photographs of the Luoquan glacial deposits, North China. (D) Field photograph of the Hankalchough glacial deposits in the Quruqtagh area, Tarim Block. (E, F) Field photographs of the Zhengmuguan glacial deposits, North China. (G) Field photograph of the Hongtiegou glacial deposits, Chaidam.

**Figure 2.**
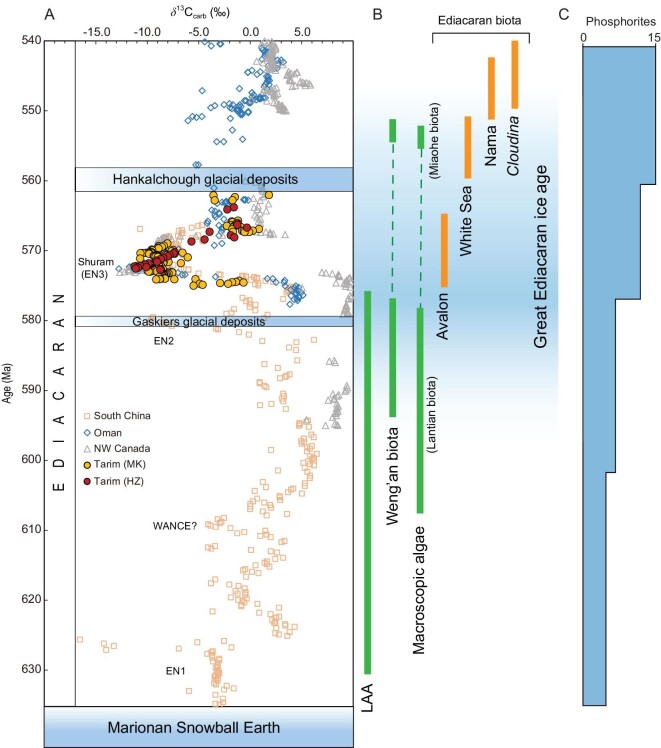
The glaciations, carbonate carbon isotope chemostratigraphy, biological evolution and phosphorite deposits of the Ediacaran period. (A) Four negative carbon isotope excursions (i.e. EN1, WANCE, EN2 and EN3) in the Ediacaran period and two typical Ediacaran glacial deposits (i.e. Gaskiers and Hankalchough glaciations). Carbon isotope data are from Refs [32,37] and references therein, and this study. (B) Typical Ediacaran fossil records. (C) Phosphorite deposits of the Ediacaran period (modified from Refs [77,78]).

Understanding the nature of glaciation throughout Earth history is important, as its waxing and waning dynamics represent one of the most fundamental controls on climate and hence on the evolution of life. Geologic evidence and climate models indicate that low-latitude glaciation, i.e. ice sheets extending to sea level in tropics, occurred under the Snowball Earth climatic condition [1,2], during which Earth's surface was completely or mostly frozen for tens of millions of years [3,4]. Geochronological and paleomagnetic data confirm the occurrences of two Snowball Earth glaciations in the Neoproterozoic: the Sturtian (717–660 million years ago [Ma]) and Marinoan (650/641–635 Ma), bookending the Cryogenian Period [5,6]. Widespread glacial deposits during the succeeding Ediacaran Period (635–538 Ma) appear to imply the existence of a younger Neoproterozoic ice age (Fig. 1; Table S1) that may have coincided closely with the soft-bodied Ediacara biota, the earliest complex macroscopic life forms in Earth history. Yet, neither the spatial extent nor the temporal duration of Ediacaran glaciation has been well constrained. On one hand, global, and even regional, correlations of Ediacaran glacial deposits remain controversial [5,7,8]. It is unclear whether all Ediacaran glacial deposits can be correlated with the ca. 580 Ma Gaskiers glaciation (Fig. 2), which is the only occurrence with a precise age constraint [8], or whether there were multiple episodes of glaciation [7,9–11]. On the other hand, although some Ediacaran glacial deposits have been mapped in the tropics [6], there is general consensus that the Ediacaran ice age was not a Snowball Earth glaciation [8].

A non-Snowball-Earth condition for the Ediacaran ice age is supported by the fossil record, which exhibits uninterrupted biological evolution (Fig. 2). The Ediacara biota first evolved with the Avalon assemblage [12–14] that occurred shortly after (within 5 million years [My]) the Gaskiers glaciation at ca. 580 Ma, diversified with the White Sea assemblage at 560–550 Ma, and declined in diversity in the Nama assemblage at 550–538 Ma before going extinct [15,16]. The same evolutionary window also witnessed the diversification of macroscopic algae [17,18] and acanthomorphic acritarchs [19], followed by the subsequent Cambrian Explosion of the earliest macroscopic biomineralization (e.g. *Cloudina* and associated small shelly fossils) [20,21] and bilaterians [22–24]. Finally, unlike the pervasive depositions of cap carbonate sharply overlying Sturtian and Marinoan glacial deposits with characteristic negative (or close to the mantle value of −5‰) carbonate carbon isotopes (δ^13^C_carb_) [3,25,26], most Ediacaran glacial deposits do not have cap carbonates, and the rare cap carbonates that might occur above Ediacaran glacial deposits do not exclusively have negative δ^13^C_carb_ values and/or have poor age constraints [27,28] (Supplementary Data).

Debate over the extent of Ediacaran glaciation is largely attributable to the absence of precise geochronological and biostratigraphic constraints on most Ediacaran glacial deposits. Only the Gaskiers glacial deposit of Newfoundland is precisely dated between 580.90 and 579.88 Ma, suggesting a short-lived ∼1-My-long glaciation [8]. But there are no other radiometric ages precise enough to determine whether the Ediacaran glacial deposits on other continents could be correlated with the Gaskiers glaciation (Table S1). Similarly, although the latest unambiguous Ediacaran fossils (<550 Ma), such as *Shaanxilithes* and *Charnia*, have been discovered above some glacial deposits (e.g. Zhengmuguan Formation [Fm] in North China and Hongtiegou Fm in Chaidam), these fossil records only place minimum age constraints on those Ediacaran glaciations [29,30].

δ^13^C_carb_ chemostratigraphy may provide additional age constraints on the Ediacaran glaciations in carbonate-dominated successions. The Ediacaran δ^13^C_carb_ chemostratigraphic framework is characterized by four negative carbon isotope excursions (CIEs, i.e. EN1, WANCE, EN2 and EN3) [31,32] (Fig. 2). The fourth, the Shuram CIE, represents the largest negative CIE in Earth history and might have recorded a prominent oxidation of the Ediacaran ocean [32–35]. The Shuram CIE can be easily identified by its extremely low δ^13^C_carb_ values (<10‰) (among other diagnostic attributes; Supplementary Data), warranting its utility for chemostratigraphic correlation [33,36]. The Shuram CIE has been dated, with Re-Os geochronology in Oman and Canada, at between 574.0 ± 4.7 Ma and 567.3 ± 3.0 Ma [37], and further constrained with astrochronology to have an identical duration from four continents of ∼8 My, confirming its global synchronicity between 570.2 ± 1.1 Ma and 562.5 ± 1.1 Ma [38]. Although further comparison between the uncertainties of both these studies awaits, the Shuram CIE is well constrained to have occurred between ca. 571 and 562 Ma [39]. While it has become increasingly apparent that the role of numerous carbonate-platform-related processes in Neoproterozoic δ^13^C excursions is critical for understanding the implications for the global carbon cycle [40–43], the global synchronicity of most of the aforementioned excursions is generally accepted, in particular the Shuram excursion, which is demonstrably globally synchronous [37,43,44] and therefore useful for correlation purposes.

In this study, we report the chemostratigraphy of the Shuiquan Fm that underlies the Hankalchough glacial deposits in the Tarim Block, northwestern China. Identification of the Shuram CIE in the Shuiquan Fm establishes that the Hankalchough glaciation postdates the ca. 571–562 Ma excursion, thus rendering the Hankalchough glaciation ca. 20 My after the short-lived ca. 580 Ma Gaskiers glaciation. On this basis, as our new robust age constraints on the Hankalchough glaciation do not permit correlation with the Gaskiers, we can first reject the possibility of only one Ediacaran glaciation. To further resolve this time gap between the highly diachronous Ediacaran glacial deposits, we integrate available geochronological and high-quality paleomagnetic data, the latter revealing a systematic global reorientation of the continents from ca. 590–580 to 560 Ma, to refine the spatiotemporal pattern of Ediacaran glaciation as it migrated across different continents transiting polar–temperate latitudes. Given a non-Snowball-Earth condition, the global propagation of the ages and durations of Ediacaran glaciation from continent to continent shows a diachronous but continuous Ediacaran ice age lasting from >580 Ma to at least 560 Ma.

## CHEMOSTRATIGRAPHIC CONSTRAINT OF THE HANKALCHOUGH GLACIATION

**Figure 3.**
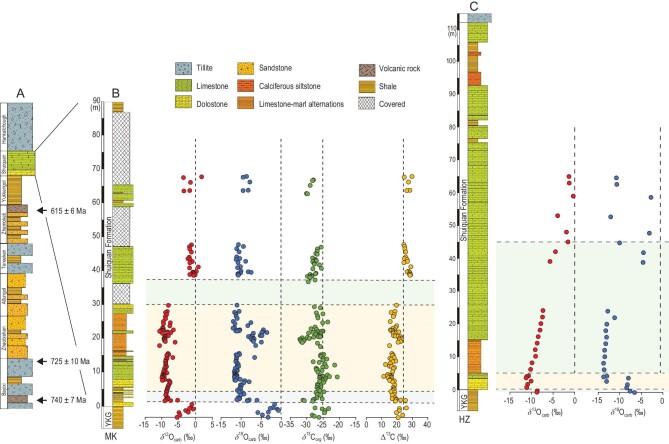
Chemostratigraphic profiles of the Shuiquan Formation at the Mochia–Khutuk (MK) and Heishan–Zhaobishan (HZ) sections. (A) Lithostratigraphy of the Neoproterozoic Quruqtagh Group. (B) Chemostratigraphic profiles of the Shuiquan Formation at the MK section. (C) Chemostratigraphic profiles of the Shuiquan Formation at the HZ section. A rapid drop (gray shadow) and gradual recovery (green shadow) in δ^13^C_carb_ values, a prominent δ^13^C_carb_ negative excursion to −10‰ (yellow shadow), the decoupling of δ^13^C_carb_ and δ^13^C_org_, and the concurrent negative excursion in δ^18^O indicate the Shuram excursion below the Hankalchough Formation, confirming the post-SE glaciation in Tarim.

The Quruqtagh Group of the Tarim Block contains three late Neoproterozoic glacial deposits, in a stratigraphic order: the Beiyixi, Tereeken and Hankalchough formations. Constrained by U-Pb zircon dating of interbedded volcanic layers and carbon isotopes on cap carbonates, the older two glacial intervals can be correlated with the Sturtian and Marinoan Snowball Earth glaciations [45,46], respectively, while the youngest Hankalchough glaciation must be younger than 615 ± 6 Ma [45]. Potential synchroneity with the ca. 580 Ma Gaskiers glaciation is therefore permissible but unknown. The Hankalchough Fm conformably overlies the carbonate-dominated Shuiquan Fm and unconformably underlies the black shale of the early Cambrian Xishanblac Fm [46] (Figs 3A, S1 and S2).

We measured δ^13^C_carb_ of the Shuiquan Fm from two sections. In the Mochia–Khutuk (MK) section, δ^13^C_carb_ increases from −5‰ to ∼0‰ in the basal 3 m, and sharply decreases to −10‰ within 1 m. Such low δ^13^C_carb_ values persist over the next 25 m. After a subsequent sharp increase, δ^13^C_carb_ remains nearly invariant between −2‰ and 0‰ in the rest of the Shuiquan Fm (Fig. 3B). In the Heishan–Zhaobishan (HZ) section, low δ^13^C_carb_ values of −10‰ begin at the base and continue for ∼5 m, followed by a slow increase to ∼0‰ in the rest of the Shuiquan Fm (Fig. 3C).

Compared with the global stack of the Shuram CIE, which is characterized by an initial rapid drop of δ^13^C_carb_ values and a more gradual recovery (Fig. S6; Supplementary Data), the Tarim δ^13^C_carb_ profile does not exhibit the initial excursion in the HZ section, while the recovery stage is not recorded in the MK section (Fig. 3). The latter might be attributed to the poor outcrop (thus not sampled) or the existence of a depositional hiatus. In addition, the Shuiquan Fm represented a depositional transition from siliciclastic to carbonate facies, and an earlier initiation of carbonate deposition in the MK section may explain the absence of the initial excursion in the HZ section (Fig. 3). Alternatively, given a prominent δ^13^C_carb_ depth gradient in the stratified Ediacaran ocean [47], the initial Shuram signal might be obscured in the deeper water HZ section [48]. Either way, the persistently very low δ^13^C_carb_ values of ∼−10‰, lasting for 25 m in the MK section and ∼5 m in the HZ section (Fig. 3), resemble the Shuram CIE observed in other localities globally (Fig. 2; Supplementary Data) rather than the older CIEs earlier in the Ediacaran that are of lesser magnitude, shorter duration and/or are too old for the maximum age constraint of 615 Ma (Fig. 2).

Correlation of the negative δ^13^C_carb_ excursion in the Shuiquan Fm with the Shuram CIE is also supported by geological and additional geochemical evidence. (i) Similar to other Shuram CIE sections globally, the Shuiquan Fm is dominated by limestone, representing deposition in a shallow marine environment (Supplementary Data) [33]. (ii) There is a positive correlation between δ^13^C_carb_ and δ^18^O (Figs S5 and S6) [33,36]. Notably, the negative δ^13^C_carb_ excursion in both the MK and HZ sections is associated with a concurrent decrease in δ^18^O values (as low as −15‰), but δ^18^O does not shift back during the Shuram recovery (Fig. 3). Such δ^13^C_carb_–δ^18^O stratigraphic trends are similar to those of the Shuram CIE in South China, Canada and Siberia [49]. (iii) δ^13^C_carb_ and organic carbon (δ^13^C_org_) isotopes are decoupled in the Shuiquan Fm with δ^13^C_org_ varying irregularly and only slightly between −25‰ and −30‰ during the Shuram CIE [50] (Figs 3 and S6). Therefore, the presence of the ca. 571–562 Ma Shuram CIE in the Shuiquan Fm implies that the overlying Hankalchough glacial diamictite represents a post-Shuram glaciation (Fig. 2).

Our study indicates a large ca. 20 My difference in age between the Gaskiers and Hankalchough glaciations (Fig. 2). The Hankalchough glaciation has been correlated with those documented in Saudi Arabia, Cadomia, Chaidam, North China and West Africa [9–11] (Table S1), but some occurrences, such as Chaidam and North China, are only loosely constrained by biostratigraphy, which provides only minimum constraints on the ages of glaciation [29,30,51]. Thus, it remains unclear whether there were just two episodes of glaciation at 580 Ma and <562 Ma or a potentially continuous succession of multiple glaciations occurring at least somewhere globally, spanning ca. 20 My. The ages and durations of other Ediacaran glaciations need to be further constrained.

## PALEOGEOGRAPHIC CALIBRATION OF AGES OF EDIACARAN GLACIATIONS

**Figure 4.**
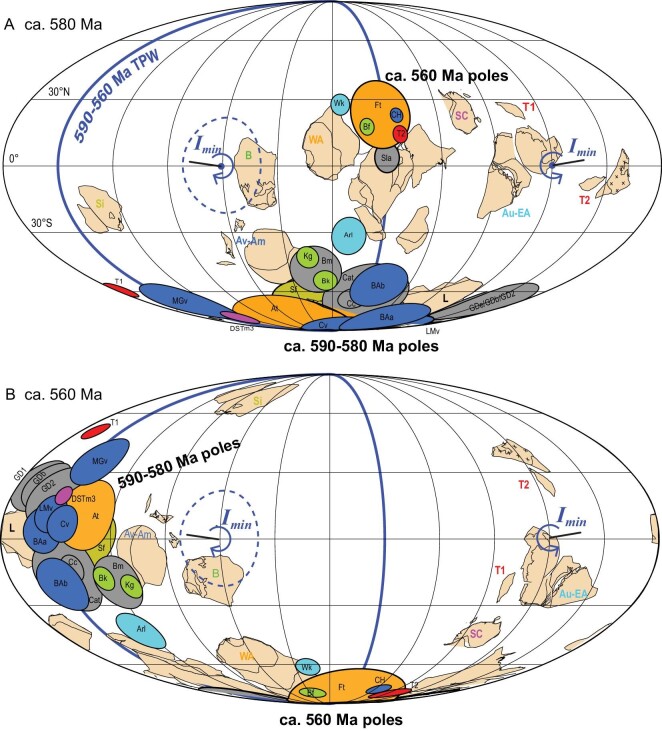
Ca. 580–560 Ma TPW-portion great circle fitted by a global composite apparent polar wander path. (A) Ca. 580 Ma paleogeographic reconstructions. (B) Ca. 560 Ma paleogeographic reconstructions. *I_min_* is the minimum-inertial moment axis, with 95% confidence ellipse in an absolute reference frame. *I_min_* is calculated as the pole to the great circle by fitting the 590–560 Ma paleopoles (Fig. S7). Two alternative (T1, T2) positions for the Tarim Block are considered (Supplementary Data). More details are listed in Tables S4 and S5. NC-North China, T-Tarim, Au-Australia, EA- East Antarctica, WA-West Africa, SC-South China, B-Baltica, Am-Amazonia, Av-Avalonia, Si-Siberia, L-Laurentia. All data are from Ref. [84] and references therein.

We integrate available geochronological (including radiometric dating, biostratigraphy and chemostratigraphy) (Table S1) and paleomagnetic data (Table S4) to refine the ages of Ediacaran glaciations. Compared to low-latitude glaciation in the Paleoproterozoic (Siderian Period) and the Neoproterozoic (Cryogenian Period) when the largest number of glacial deposits occurred within the tropical climate belt (0–30°), very few tropical glacial deposits occurred during the three Phanerozoic ice ages, which were dominated by glaciation in the polar (60–90°) and temperate (30–60°) climate belts [52]. Given the precedent of this stark contrast in the two latitudinally based styles of glaciation in Earth history, and the aforementioned paleomagnetic, biostratigraphic and geochemical evidence against low-latitude glaciation in the Ediacaran, we assume a non-Snowball condition ruling out (or limiting) tropical Ediacaran glaciation. Due to the occurrence of appreciable paleomagnetic apparent polar wander (APW) during the Ediacaran (Fig. 4) [53,54], continental positions changed quickly such that these time-varying latitudinal constraints can be used to calibrate the highly uncertain glacial ages.

In order to be used as a reliable paleogeographic reference frame, paleomagnetic data rely on the fidelity of the assumption of the geocentric axial dipole (GAD) hypothesis. The aforementioned anomalous APW during the Ediacaran has inspired a range of interpretations, some of which call into question the fundamental GAD assumption, and therefore warrant discussion. The best dated and sampled Ediacaran APW path is that of Laurentia, but even its interpretation has been debated due to the occurrence of both high and low paleolatitudes. An oscillation between axial and equatorial dipoles has been proposed to explain the largely bimodal paleolatitudes of Laurentia [55]. However, equatorial dipoles have only been observed on gas giant planets with un-Earth-like inner and outer cores and the long durations of both pole groups of Ediacaran APW for Laurentia greatly exceed Cenozoic equatorial dipole flux patches on Earth (see discussion in Ref. [53]). Also, the similarly large Ediacaran APW path of Australia is expressed in terms of changes in declination, not inclination, and is therefore incompatible with alternating equatorial-polar dipoles.

The reliability of the magnetic field during the Ediacaran has also been questioned on the basis of the very weak paleointensity estimates of the ca. 565 Ma Sept-Îles layered mafic intrusive complex of ∼7 ZAm^2^, which are among the lowest values in the paleointensity database [56,57]. The hypothesized geomagnetic instability possibly caused by inner core nucleation in the Ediacaran [56] has, however, been challenged by strong paleointensity estimates at ca. 1.1 billion years ago on par with younger ages for which the existence of a crystallizing inner core is accepted [58]. Although we cannot discount all non-uniformitarian magnetic field hypotheses for Ediacaran time, we cannot reject the null hypothesis of a GAD field that, according to the paleolatitudes of climate-sensitive evaporites, appears to have been operational for the past 2 billion years [59]. Finally, the existence of a weak field at one age does not invalidate the GAD assumption on which several tens of high-quality paleomagnetic poles for the Ediacaran are based.

Yet another complication that needs to be addressed has plagued the Ediacaran paleomagnetic data of Laurentia, namely high-latitude overprinting, and again the Sept-Îles complex offers an illustrative example. Two magnetic remanences were identified: a low-latitude ‘A’ remanence from the intrusion and a high-latitude ‘B’ remanence from undated dikes [60]. The original study interpreted the high-latitude ‘B’ remanence as an overprint [60] and subsequent studies have generally upheld this interpretation [57,61]. As with its aforementioned single spot-reading of a weak field, the one example from the Sept-Îles complex of a high-latitude overprint for Laurentia, nonetheless, does not invalidate the multiple high-latitude poles from Laurentia that are either demonstrably, or most likely, primary [53]. For example, the high-latitude Grenville dikes ‘B’ remanence represents one of the most detailed positive baked contact tests in the Precambrian paleomagnetic database [62]. Therefore, while important to address, high-latitude overprinting cannot account for otherwise credible high paleolatitudes recorded for Laurentia during the mid-Ediacaran.

Establishing the fidelity of the Ediacaran poles considered in this study, another explanation for anomalous Ediacaran APW is true polar wander (TPW), or planetary reorientation [54,63]. As it entails wholesale motion of the entire solid Earth (mantle and crust) around the liquid outer core, TPW is detectable by paleomagnetism and should pass a global reproducibility test. In addition to the ∼90° spread of the paleomagnetic poles of Laurentia, similar large APW paths have been identified on four other continents: Australia, Avalonia, Baltica and West Africa (Fig. 4). As paleomagnetism measures both TPW and plate motion (APW = plate motion + TPW), the largely stationary hotspot track of the geophysically and geochemically inferred Sutton plume of Laurentia requires that a majority of the large APW was due to TPW [53]. The Ediacaran TPW hypothesis has been called into question [57] on the basis that the rapid APW rates violate the TPW speed limit imposed by present-day mantle viscosity (2.4° My^−1^) [64]. Nonetheless, due to well-established secular mantle cooling (∼100 °C per billion years) [65], a ∼50 °C hotter mantle in the Ediacaran would have been (assuming temperature-dependent viscosity) roughly half as viscous and therefore able to deform into a reoriented hydrostatic figure and drive TPW about twice as fast as today. While it would take nearly ∼40 My for a ∼90° reorientation to occur today, it would have only taken ∼20 My in the Ediacaran, precisely as the paleomagnetic data indicate (Fig. 4). Therefore, taking secular mantle cooling into account, the Ediacaran TPW hypothesis is theoretically plausible. The excitation for such dramatic Ediacaran rotational instability may be attributed to the large mass anomalies that would have been involved in the subducting slabs sinking through the mantle during the late Neoproterozoic assembly of megacontinent Gondwana [54]. Although all TPW, rotating about an equatorial axis defined by Earth's minimum moment of inertia (*I_min_*), involves a change in the relative identities of the other two orthogonal intermediate and maximum inertial axes when they become subequal (*I_int_*  $\approx $  *I_min_*), if the non-hydrostatic inertia tensor does not change after TPW begins then a complete ∼90° so-called ‘inertial interchange’ TPW event, or IITPW, occurs as it did in the late Ediacaran [66]. Provided the aforementioned caveats, the Ediacaran TPW hypothesis is not regarded as a fait accompli and further testing of multiple hypotheses should continue to be considered. Nevertheless, we suggest that the availability of enough credible paleomagnetic data from multiple continents exhibiting large angular dispersions is strongly suggestive of TPW warrants consideration of the paleoclimatic and biogeographic implications of such a large-scale paleogeographic reorientation.

**Figure 5.**
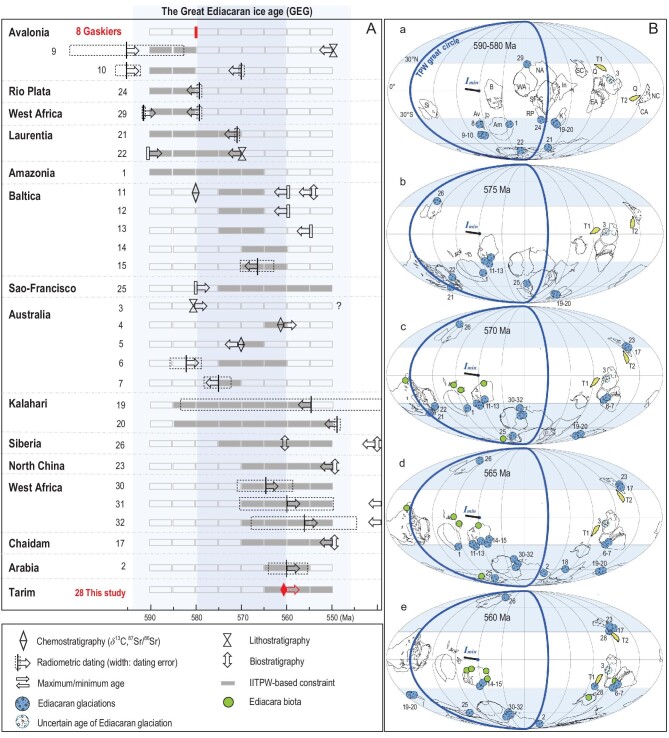
Spatiotemporal distributions for the prolonged (≥20 My) Ediacaran ice age in a continuously TPW-based (‘absolute’) framework from ca. 590–580 to 560 Ma. (A) Age constraints of Ediacaran glacial deposits in different continents. The age constraints include radiometric dating, chemostratigraphy, biostratigraphy and regional stratigraphic correlation, and are listed in Table S1. Precise radiometric age constraints of the Gaskiers glaciation in Avalon and chemostratigraphic constraint of the Hankalchough glaciation in Tarim (this study) are marked in bold red. Further IITPW-based temporal distributions are marked by gray boxes. The dark blue shadow marks the confirmed range, while the light shadow marks the possible extended range of the Great Ediacaran ice age. (B) Paleogeographic reconstructions based on IITPW in the late Ediacaran. Glacial deposits are numbered, and are listed in Table S1. The localities of Ediacara biota are modified from Refs [15,80,85]. The Avalon assemblage is plotted in 5c and 5d, and the White Sea assemblage is placed in 5e. NC-North China, T-Tarim, Q-Quruqtagh, Au-Australia, EA-East Antarctica, WA-West Africa, NA-Northeast Africa, In-India, SC-South China, SF-Sao Francisco, C-Congo, RP-Rio Plata, K-Kalahari, B-Baltica, Am-Amazonia, Av-Avalonia, Si- Siberia, L-Laurentia.

We propose that the spatiotemporal distributions of Ediacaran glacial deposits were largely controlled by the rapid latitudinal change of continents (∼90° TPW event) from ca. 590–580 to 560 Ma. For example, the 590–580 Ma glaciations in Avalonia and Laurentia occurred at mid-to-high latitudes (>30°) [8,12,67] (Fig. 5Ba); meanwhile, the absence of younger glacial deposits after 580 Ma on these continents can be attributed to their rotating (via TPW) into the tropics (<30°; Fig. 5Bc, d, e). The post-Shuram glaciations—including the Luoquan and Zhengmuguan formations of North China [51], the Hongtiegou Fm of Chaidam [29,30], the Hankalchough Fm of Tarim [46], the Billy Springs Fm in South Australia [68], the Dhaiqa Fm of Arabia [27] and the Ouarzazate Group of West Africa [69] (Table S1)—coincided with the rotation of these once-tropical continents out of the tropics by the end of the TPW event by ca. 560 Ma (Fig. 5Be).

To further constrain the ages of poorly dated Ediacaran glaciations using the systematic time-varying latitudinal changes associated with the ∼90° TPW rotation, paleomagnetic-based paleogeographic maps in 5 My increments (of equal angular rotation) from ca. 590–580 to 560 Ma were constructed independently of geochronological glacial constraints. Since a non-Snowball-Earth condition precludes (or limits) low-latitude glaciation at this age [3,4], the timing of each Ediacaran glaciation can be further refined by ascribing a possible age range to each deposit for which the set of five paleogeographic maps places each occurrence in mid-to-high latitudes (Fig. 5). By this non-Snowball rule, a two-glaciation scenario can be rejected by its failing to place nearly all glacial depositions in polar–temperate regions in either of the 590–580 Ma or 560 Ma paleogeographic maps (Fig. S8). In particular, glacial deposits of South Australia (Bunyeroo formations), Tasmania (Croles Hill Formation) and Baltica (Moelv, Mortensnes, Vil’chitsy, Kurgashlya and Tanin–Starye Pechi formations) are located in tropics in both endmember maps (Fig. S8). In contrast, treating age of these poorly dated units as a free parameter, these glacial deposits would not violate the conjectured non-Snowball condition if they occurred at 570 Ma or 565 Ma (Fig. 5Bc, d).

**Figure 6.**
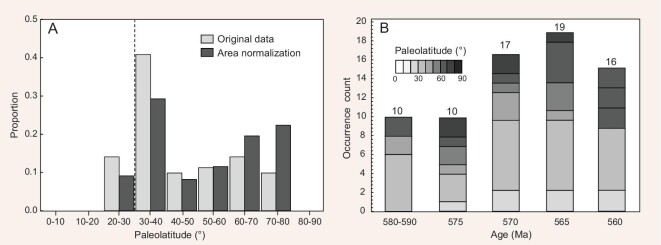
A histogram of spatiotemporal distributions for Ediacaran glacial deposits. (A) Spatial distributions of Ediacaran glacial deposits. Light shadows represent original data from Fig. 5 and dark shadows are from latitude band-area normalization of the paleolatitude estimates. (B) Temporal distributions of Ediacaran glacial deposits. Counts represent possible occurrences of each time bin.

All Ediacaran glacial deposits, except for the Egan Fm in northwest Australia (see details in Supplementary Data), can be placed in mid-to-high latitudes for at least one age interval, and each age interval has at least five possible occurrences of mid-to-high-latitude glaciation (Fig. 5). Plotted as a histogram of permissible paleolatitudes (Fig. 6A), Ediacaran glaciation occurred continuously throughout temperate and polar latitudes, where the lack of occurrences within 10° of the poles may be attributable to this bin size having the smallest area. (Not too much should be made of the small handful of possible occurrences in the tropics due to the assumption of the non-Snowball rule.) Interestingly, the latitude bin of the greatest number of possible glacial occurrences is just beyond the tropics between 30–40°, possibly indicating that Ediacaran glacial deposits largely record deglacial facies as continents glaciated at high- to mid-latitudes transited into lower mid-latitudes and melted. Thus, taking TPW into account not only reconciles the strong diachroneity between the ca. 580 Ma Gaskiers glaciation and the <560 Ma Hankalchough glaciation constrained herein, but also potentially identifies other continents with similar ages of early and late glaciation as well as those with intermediate ages of glaciation in between. This observation of a global spatiotemporal pattern may imply continuously migrating Ediacaran glaciation for >20 My (Fig. 6B).

## GREAT EDIACARAN GLACIATION

We propose a diachronous but continuous ice age, collectively called the ‘Great Ediacaran Glaciation’ (GEG), that spatiotemporally migrated across all paleocontinents from >580 Ma to <560 Ma (Figs 5 and 6), broadly consistent with the prior identification of at least four glacial advances during the late Ediacaran [7]. Lasting a minimum of 20 My, the GEG is shorter than the Sturtian and longer than Marinoan Snowball Earths, with a duration on par with some of the longer Phanerozoic ice ages [70–72]. In fact, both the migration of glacial centers across Gondwana during the late Paleozoic ice age and the onset of northern hemisphere glaciation during the Cenozoic ice age have also been attributed to TPW [52,73]. Though similar to some Phanerozoic ice ages in terms of long duration and TPW forcing, ice sheets of the GEG apparently extended to lower mid-latitudes between 30–40° (Fig. 6A), suggesting different glacial dynamics than during Phanerozoic ice ages. In contrast to the full ∼90° IITPW event in the Ediacaran, all putative TPW events during the Phanerozoic are of smaller amplitude (≤60°) [74] which may account for this difference in glacial latitudes.

Such an icehouse background for the rise of large organisms may require an important link between the GEG and biotic evolution through biogeochemical cycles. Most notably, the large-amplitude and long-lived Shuram CIE occurred squarely within the GEG (Fig. 2). In fact, plotting a histogram of the paleogeographically constrained glacial age ranges (Fig. 6) demonstrates that the largest number of potential occurrences coincide with the ca. 571–562 Ma Shuram excursion. No consensus exists for the origin of the enigmatic Shuram CIE, but two prevailing interpretations both have potential connections with the GEG. Traditionally, it was thought that the Shuram CIE might reflect the oxidation of a massive pool of dissolved organic carbon (DOC) in the deep ocean [34,75], which is generally still supported by the occurrence of seafloor anoxia before and after Shuram, but not during [37]. It is plausible that efficient, long-lived and widespread glacial weathering associated with the GEG might have increased the runoff and supply of marine nutrients (e.g. phosphorus), allowing P accumulation in the surface ocean, and accordingly would persistently sustain high primary productivity and oxygenation in the atmosphere. High seawater P concentration during the GEG is supported by the global phosphate deposition in the late Ediacaran [76–78] (Fig. 2C). More recently, it has been recognized that carbonate platforms may involve processes that produce isotopically anomalous ^13^C values without increasing global oxygen production, such as diurnal carbon cycling [41] and various forms of diagenesis [79] that are likely to be amplified in carbonate platforms during the GEG. Critically, establishing the boundary condition of the long-lived global propagation of the GEG provides key information for assessing potential links between the myriad dramatic biogeochemical events of the late Ediacaran.

Unlike the younger Shuram CIE, which is divorced from the radiation of the earliest Ediacaran fauna [37], the onset of the GEG either immediately preceded or was coincident with its first appearance (the Avalon assemblage) shortly after the Gaskiers glaciation (Fig. 2). The duration of the rest of the GEG also overlapped with the entire evolutionary window of the Ediacaran biota, which subsequently diversified at ca. 560 Ma (the White Sea Assemblage), and then had its diminishing depauperate finale after ca. 550 Ma (the Nama Assemblage) (Fig. 2; see ref [80] for discussion of potential slight chronostratigraphic overlaps between assemblages). Thus, we suggest that the spatiotemporal propagation of the GEG can account for the heretofore enigmatic diachroneity of the three Ediacaran biotic assemblages. Figure 5B shows a strong anticorrelation between glacial deposits (generally >30°) and Ediacara biota (generally <30°), implying that TPW-driven deglaciation provided an ideal ecospace and environment for life. Continents moving into low latitudes (e.g. Laurentia and Avalon) or rotating within mid-to-low latitudes (e.g. Australia and South China) would have been more likely to experience species origination, as observed, due to the latitudinal diversity gradient [81]. Thus, we suggest the morphological diachroneity of the Ediacara biota reflects to some degree the unique glacial–deglacial dynamics of the GEG, as continents rapidly transited polar-to-temperate latitudes.

## CONCLUSION

In this study, we report the presence of a globally synchronous ca. 571–562 Ma Shuram CIE, the largest negative carbonate carbon isotope excursion in Earth history, stratigraphically below the Ediacaran Hankalchough glacial deposits in the Tarim Block, confirming a post-560 Ma glaciation. The Hankalchough glaciation is a sharp contrast to the ca. 580 Ma Gaskiers glaciation. Given a non-Snowball-Earth condition ruling out (or limiting) the occurrence of low-latitude glaciation, the age ranges of ∼30 Ediacaran glaciations are further refined by tracking systematic latitude changes throughout a ∼90° TPW event from ca. 590–580 to 560 Ma. We demonstrate a diachronous but continuous ‘Great Ediacaran ice age’ lasting >20 My during this interval of late Ediacaran time. Moreover, the GEG bracketed the evolutionary window of the Ediacara biota, which may suggest that glaciation–deglaciation played a heretofore underappreciated role in the dramatic changes in the late Neoproterozoic surface environment—not only during the Cryogenian, but also during the Ediacaran.

## METHODS

### Major and minor elemental composition analyses

Carbonate samples were crushed to 200 mesh. Sample powders of 30 mg were loaded in a 15 ml centrifuge tube and dissolved in 10 ml Acetate-ammonium acetate buffer solution (HAc: NH_4_Ac = 1:5) with pH precisely adjusted to 4.5. In order to completely dissolve calcareous materials (both Ca-carbonate and dolomite), the centrifuge tubes were placed in a shaking table with a water bath temperature of 50 °C for 48 hours. After centrifugation, supernatant was collected for the element composition analysis. Elemental compositions were measured at Peking University by a Spectro Blue Sop inductively coupled plasma optical emission spectrometer (ICP-OES). All analyses were calibrated by a series of gravimetric standards with different concentrations (ranging from 0.1–10 ppm) that were run before and after every 20th sample measurements. The element concentrations were calculated with respect to carbonate mass, i.e. CaCO_3_ + MgCO_3_ = 100%. The analytical precision for major and minor elements (Mg, Ca, Fe, Mn, Al) is ±5%.

### Carbonate carbon and oxygen isotope analyses

Mirrored thin and polished thick sections were prepared. Under the guidance of petrographic observation of the thin section, sample powders were micro-drilled from the mirrored thick section to avoid sampling the diagenetic altered materials and hydrothermal veins. Carbonate carbon (δ^13^C_carb_) and oxygen (δ^18^O) isotopes were measured at the Oxy-Anion Stable Isotope Consortium (OASIC) of Louisiana State University by a Gas Bench coupled with Thermal Fisher Delta V isotope ratio mass spectrometry (IRMS). About 0.2 mg of carbonate powder was loaded in a headspace vial, and dried at 70°C for 24 hours. Five drops of phosphoric acid were added to the vial, and carbonate was converted into CO_2_ after reaction at 70°C. The isotope ratios were reported in delta-notation as per mil deviation relative to the V-Pee Dee Belemnite (V-PDB) Standard. The analytical precisions for δ^13^C_carb_ and δ^18^O are ±0.08‰ and ±0.1‰, respectively.

### Organic carbon isotope analyses

Fresh carbonate chips were crushed to 200 mesh, and ∼30 mg sample powder was leached by 30 ml 3 N HCl to dissolve all calcareous contents. The solution was centrifuged for 10 minutes at 3000 r/min and washed by deionized water three times until pH > 5. Insoluble residues were dried down at 60°C in an oven. Organic carbon isotope (δ^13^C_org_) values were analyzed by a Vario Microcube Elemental Analyzer coupled with Isoprime 100 isotope ratio mass spectrometry at the OASIC of Louisiana State University. The isotope ratios were calibrated by the standard Acetanilide-OASIC (−27.62 ± 0.02‰), and the analytical precision for δ^13^C_org_ is ±0.1‰.

### Paleogeographic reconstructions

This IITPW movement involves a wholesale rotation of the solid Earth (mantle and crust) about the minimum-inertial moment axis (*I_min_*) to align Earth's maximum moment of inertia (*I_max_*) with the spin axis [82,83]. The paleomagnetic poles for the reconstruction are filtered from the global Precambrian database (PALEOMAGIA; https://h21.it.helsinki.fi/index.php). Pole compilation and reconstruction details are provided in the Supplementary Data. Both the reconstruction and paleogeographic maps are processed using GPlates freeware (http://www.gplates.org).

## Supplementary Material

nwad117_Supplemental_File
