# Supplementary material for: A Great late Ediacaran ice age
Source: Natl Sci Rev. 2023 Apr 25;10(8):nwad117. doi: 10.1093/nsr/nwad117 (PMC10306365; doi:10.1093/nsr/nwad117)
Supplement: nwad117_Supplemental_File [file nwad117_supplemental_file.pdf]

Supplementary Information for

A Great late Ediacaran ice age

Ruimin Wang, Bing Shen, Xianguo Lang, Bin Wen, Ross N. Mitchell, Haoran Ma, Zongjun Yin, Yongbo Peng, Yonggang Liu, Chuanming Zhou

Corresponding author: B. Shen; X. Lang and B. Wen

Email: B. Shen (bingshen@pku.edu.cn), X. Lang (langxianguo19@cdut.edu.cn), B. Wen (wenbin@cug.edu.cn)

This PDF file includes:

Supplementary text
Figures S1 to S8
Tables S1 to S5
SI References

Supplementary Information Text

1. Geological setting and section description

The Neoproterozoic succession in the Quruqtagh area of Tarim Block (NW China; Fig.S1), i.e., the Quruqtagh Group, is composed of, in an ascending order, the Beiyixi, Zhaobishan, Aletonggou, Tereeken, Zhamoketi, Yukengou, Shuiquan and Hankalchough formations. The total thickness of the Quruqtagh Group is >2700m [1]. The Beiyixi, Tereeken and Hankalchough formations, composed of diamictite, pebbly sandstone, and intercalated siltstone and sandstone, represent three glacial intervals, i.e., the Beiyixi, Tereeken, and Hankalchough glaciations [2]. The Beiyixi glaciation was dated between 740 ± 7 Ma and 725 ± 10 Ma [3]. The Tereeken Formation underlies a 5-10 m-thick cap carbonate of the basal Zhamoketi Formation [4], which was proposed to be correlated with the Marinoan cap carbonate, and thus the Tereeken glaciation may be equivalent to the Marinoan glaciation. Such correlation is consistent with a zircon U-Pb age of 615 ± 6 Ma from the overlying Zhamoketi Formation [3]. The Hankalchough Formation conformably overlies the Shuiquan Formation and unconformably underlies the early Cambrian Xishanblac Formation. Therefore, the Hankalchough glaciation was an Ediacaran glaciation in the Tarim Block.

At the Mochia-Khutuk section (MK, 41°26 '29 "N, 87°51' 47" E), the Shuiquan Formation conformably overlies the dark gray siltstone/mudstone of the Yukengou Formation, and is divided into five lithological units (Figs. S2 and S3). The unit 1 (U1) consists of intercalated thin-bedded microcrystalline limestone and shale at the bottom and the reddish thin- to medium-bedded coarse crystalline dolostone at the top. The unit 2 (U2) is composed of medium- to thick-bed laminated limestone. The overlying unit 3 (U3) is characterized by limestone-marl alternations that consist of alternating depositions of cm-thick reddish-brown marlstone and light-gray limestone layers. The unit 4 (U4) is composed of thin-bedded limestone with some degree of silicification. The top of Shuiquan Formation (U5) is mainly composed of black calcareous siltstone intercalated with thin-bedded lime mudstone layers.

In the Heishan-Zhaobishan section (HZ, 41°28 '18.6 "N, 88°18' 14.5" E), the Shuiquan Formation can also be divided into five lithological members (Figs. S2 and S3), in stratigraphic order, the reddish-brown dolostone member with limestone layers at the top (M1, 5 m), the

limestone-marl alternations member (M2, 10 m), the thin-bedded limestone member with occasional occurrences of shale beds (M3, 78 m), the shale-calcareous siltstone member (M4, 12 m), and the upper thin-bedded limestone member (M5, 5 m).

The MK carbonate may deposit in shallower water than the HZ section. This interpretation is mainly supported by the following sedimentological evidence: (i) The overlying Hankalchough diamictite thickens eastward from ~50 m to ~400 m, indicating deeper basin and more accommodation to the east [5]. (ii) In the overlying Hankalchough cap carbonate, the size and content of siliciclastic component decreases from west to east, suggesting the eastward transport of siliciclasts [2]. (iii) Laminar algal dolostone and bird's eyes structure in the lower Shuiquan Formation of MK section indicate the deposition in the intertidal-supratidal environments (Fig. S4). (iv) The paleoflow direction reconstruction from the underlying Zhamoketi Formation also indicates the flow direct from southwest to northeast [6].

2. Analytical Methods for geochemical data

2.1 Major and minor elemental composition analyses

Carbonate samples were crushed to 200 mesh. 30 mg powder sample was loaded in a 15 ml centrifuge tube and was dissolved in 10 ml Acetate-ammonium acetate buffer solution (HAc:NH_4Ac=1:5) with pH precisely adjusted to 4.5. In order to completely dissolve calcareous materials (both Ca-carbonate and dolomite), the centrifuge tubes were placed in a shaking table with a water bath temperature of 50°C for 48 hours. After centrifugation, supernatant was collected for the element composition analysis. Elemental compositions were measured at Peking University by a Spectro Blue Sop inductively coupled plasma optical emission spectrometer (ICP-OES). All analyses were calibrated by series of gravimetric standards with different concentrations (ranging from 0.1-10 ppm) that were run before and after every twenty sample measurements. The element concentrations were calculated with respect to carbonate mass, i.e., $CaCO_3$+$MgCO_3$=100%. The analytical precisions for major and minor elements (Mg, Ca, Fe, Mn, Al) is ±5%.

2.2 Carbonate carbon ($\delta^{13}C_{carb}$) and oxygen isotope ($\delta^{18}O$) analyses

Mirrored thin and polished thick sections were prepared. Under the guidance of

petrographic observation of thin section, sample powders were micro-drilled from the mirrored thick section to avoid sampling the diagenetic altered materials and hydrothermal veins. Carbon and oxygen isotopes were measured at the Oxy-Anion Stable Isotope Consortium (OASIC) of Louisiana State University by a Gas Bench coupled with a Thermal Fisher Delta V isotope ratio mass spectrometry (IRMS). About 0.2 mg of carbonate powder was loaded in a headspace vial, and dried at 70°C for 24 hours. Five drops of phosphoric acid were added to the vial, and carbonate was converted CO_2 after reaction at 70 °C. The isotope ratios were reported in delta-notation as per mil deviation relative to the V-PDB Standard. The analytical precisions for $\delta^{13}C_{carb}$ and $\delta^{18}O$ are ±0.08‰ and ±0.1‰, respectively.

2.3 Organic Carbon Isotopes ($\delta^{13}C_{org}$) analyses

Fresh carbonate chips were crushed to 200 mesh, and ~30 mg sample powder was leached by 30 ml 3N HCl to dissolve all calcareous contents. The solution was centrifuged for 10 minutes at 3000 rpm and washed by Deionized water 3 times until pH > 5. Insoluble residues were dried down at 60°C in an oven. Organic Carbon Isotopes ($\delta^{13}C_{org}$) values were analyzed by a Vario Microcube Elemental Analyzer coupled with an Isoprime 100 isotope ratio mass spectrometry at the Oxy-Anion Stable Isotope Consortium (OASIC) of Louisiana State University. The isotope ratios were calibrated by the standard Acetanilide-OASIC (-27.62 ± 0.02‰), and the analytical precision for $\delta^{13}C_{org}$ is ±0.1‰.

3. Diagenetic evaluation of $\delta^{13}C_{carb}$

The investigated Shuiquan carbonates are mainly composed of dolomicrite or microcrystalline dolomite with partial recrystallization to mesocrystalline dolomite (Fig. S3). Although dolomitization is a diagenetic process, there is no evidence indicating that dolomitization would significantly modify the carbon isotopic compositions.

To evaluate whether $\delta^{13}C_{carb}$ values have been diagenetically altered, three geochemical proxies have been traditionally applied, (i) the absolute values of $\delta^{18}O$, (ii) the relationship between $\delta^{13}C_{carb}$ and $\delta^{18}O$, and (iii) Mn/Sr ratios [7-9]. In generally, carbonate samples with $\delta^{18}O > -10$ ‰, Mn/Sr ratio <10, and the absence of the positive correlation between $\delta^{13}C_{carb}$

and $\delta^{18}O$, are regarded as least altered, suggesting that although the absolute $\delta^{13}C_{carb}$ values might be altered but the stratigraphic trend was preserved [7].

There are week positive correlations between $\delta^{13}C_{carb}$ and $\delta^{18}O$, and low $\delta^{18}O$ values (ranging from −5‰ to −15‰) both in MK and HZ (Figs.2, S5, Table S2 and S3). However, carbonate depositions during the Shuram Excursion normally show a positive correlation between $\delta^{13}C_{carb}$ and $\delta^{18}O$ values, and the $\delta^{18}O$ values are normally <-10‰ [10, 11]. In fact, the global occurrence of SE is commonly considered to record a prominent ocean oxidation event rather than local diagenesis [12-15], and the primary seawater signal has been supported by various geochemical and geological evidence [16, 17]. Thus, we suggest that $\delta^{13}C_{carb}$ and $\delta^{18}O$ data could not be applied to evaluate diagenetic alteration for SE samples. In addition, it is noticed that although $\delta^{18}O$ values do not shift positively in the recovery of SE, the positive $\delta^{13}C_{carb}$ excursion is unlikely driven by a diagenetic alteration. Similar pattern is observed in SE in South China, Canada, and Siberia as well [18, 19]. Moreover, negligible diagenetic alterations could be inferred from low Mn/Sr values (<3) of most MK samples (Fig. S5).

4. The global correlations of Shuram Excursions

There are four negative $\delta^{13}C_{carb}$ excursions in the Ediacaran period, in the chronological order, EN 1 (CANCE) in the cap carbonate immediately above the Marinoan glacial deposits at ~635 Ma, EN 2 (BAINCE) represented by a brief negative $\delta^{13}C_{carb}$ excursion to ~-5‰ that might be correlated with the Gaskiers glaciation (~580 Ma), and EN 3 (DOUNCE)/Shuram Excursion (SE) characterized by a long-duration negative $\delta^{13}C_{carb}$ excursion to as low as -12‰ between ~570 Ma and ~560 Ma [12, 17], another negative excursion (WANCE) was recently reported between EN1 and EN2, and was dated at ~609 Ma [20]. Among the four Ediacaran negative excursions, SE has been widely reported, including Oman [14, 21, 22], Australia [23-25], the United States [26-28], India [29, 30], Canada [31, 32], Scotland and Ireland [33], Siberia [18, 19], Mongolia [34], South China [12, 35, 36] and Tarim (this study) (Fig. S6).

The characteristic features of SE include:

(1) **Magnitude and stratigraphic thickness.** Low $\delta^{13}C_{carb}$ values of ~-10‰ would last for 10s to 100s m in stratigraphy (e.g., ~1000 m in Siberia, ~400 m in Oman, ~600 m in the United States, ~150 m in South Australia and ~50 m in South China) (Fig. S6).

(2) **Lithology, mainly in the shallow marine carbonate successions.** In Oman, SE initiates at the limestone of Khufai Formation and continues into the alternating carbonate and siliciclastic deposition of the Shuram Formation. In the United States, SE is recorded in the intercalated carbonate and siliciclastic deposition of the Johnnie and Stirling formations. In Australia, SE occurs in the dolostone of Wonoka Formation. In South China and Siberia, SE is recorded in the limestone, dolostone, and alternated depositions of limestone and marlstone (ribbon rock) (Fig. S6).

(3) **Stratigraphic asymmetry**. SE is characterized by a rapid drop and gradual recovery in $\delta^{13}C_{carb}$ values [15, 37] (Fig. S6).

(4) **Other geochemical signals**. There are some typical geochemical features in the Shuram excursion, including the decoupling of carbonate carbon ($\delta^{13}C_{carb}$) and organic carbon ($\delta^{13}C_{org}$) isotopes [38], a strong positive correlation between $\delta^{18}O$ and $\delta^{13}C_{carb}$ [11], the concurrent decreasing of both $\delta^{34}S_{pyrite}$ and pyrite contents [12, 14, 39], and the increases of $\delta^{238}U$ and $^{87}Sr/^{86}Sr$ [39].

5. Paleogeographic reconstruction in an 'absolute' framework during the inertial-interchange true polar wander (IITPW) event from ca. 590-580 to 560 Ma

This IITPW movement involves a wholesale rotation of the solid Earth (mantle and crust) about the minimum-inertial moment axis (I_{min}) to align Earth's maximum moment of inertial (I_{max}) with the spin axis [40, 41]. Adopting the methodology of [42-45], the IITPW-movement great circle and its orthogonal axis I_{min} are defined by a composite apparent polar wander path (APWP) in a TPW-based (absolute) reference frame (Fig. S7). The most reliable paleopoles of [42-44], which includes two middle Cambrian poles from North China (Table S4), are used in this reconstruction. Through the IITPW process from ca.590-580 to 560 Ma, the entire solid Earth rotated along the great circle and different segments of a continuously kinematic paleogeography are shown in Figs.4 and 5 of the main text. Key points for the reconstruction are provided in the following:

(1) ***West Gondwana regime.*** The configuration of this regime, including the major continents of Avalonia-Amazonia, Baltica, Laurentia and their small associations (such as the Arequipa-Antofalla Terrane) [46, 47], is mainly an inherited model of [42].

(2) ***Central Gondwana***. It is widely accepted that the northern part of the Central Gondwana had welded together at least by ca. 580 Ma [48], including the (620-580 Ma) Pan-African–Brasiliano collisional belt [49, 50] and the (650-620 Ma) East African Orogeny for the accretions of the Northeast Africa, India and Madagascar [48] in its west and east, respectively. This mainland is quantitatively constrained using the paleomagnetic data for West Africa [43, 44]. To the south, although no poles obtained from Kalahari, the geological and geochronological evidence strongly supports that this craton was surrounded by open oceans (e.g., Adamastor-Khomas Ocean) until Early Cambrian [48, 51, 52].

(3) ***East Gondwana.*** The East Gondwana mainly includes Australia and East Antarctic and these two continents had shared a coherent evolution for more than 2.4 billion years before the late Cretaceous [53]. The position of this coherent is constrained by aligning the Australian poles (Au-Arl and Au-Wk) with the global APWP segment (Fig. S7), leaving an open ocean (Mawson Ocean) towards its neighboring continents of Central Gondwana [54-56].

(4) ***Asian blocks***. For the Asian blocks, including Siberia and three Chinese blocks (South China, North China and Tarim), their positions are constrained by using the paleopoles in a more comprehensive, because none of them possesses a segment of independent APWP alone in this interval (Table S4 and Fig. S7). The pole of Sc-DSTm3 from South China was obtained from the Doushantuo Formation Member 3 and represents a grand mean pole for the same rock unit [57]. Its precise age has been updated with a new geochronological study (591 ± 5 Ma) [58]. After a coincidence of this pole with the South Pole, a resemble position around India and northwestern Australia is achieved (Fig. S7) [59, 60]. For North China, an integrated paleomagnetic and paleontological study suggested that North China acted an important biogeographic link (Cambrian faunal exchange) between East Gondwana and Laurentia in the middle Cambrian [61]. For its facilitation of the migration from the conjugate margin of northwestern Laurentia in Rodinia’s configuration [62, 63], North China is positioned between East Gondwana and Laurentia by adjusting the 514-505 Ma (Nc-XW and Nc-Hf) poles close to the ca. 560 Ma group (Fig. S7). This reconstruction is further reinforced by the geo-biological evidence from the Chaidam block. On the one hand, stratigraphic records (including the post-Gaskiers Hongtiegou glaciation) and biotic assemblage (e.g., *Shaanxilithes*) in Chaidam suggest that this small block should stay together with the North China in the late Ediacaran

[64, 65]; on the other hand, the 600-580 Ma plume-related magmatism in north margin of the Chaidam-Qilian block could be well correlated with those in southeastern Australia [66]. As to the Tarim Block, there is only one pole (T-Zma) [67] from the 615 ± 6 Ma basaltic andesite of the uppermost Zhamoketi Formation during the middle- to late-Ediacaran time, and no field test is available for the age of the remanence. So, two (*'T1', 'T2'*) optional positions are provided (Figs. 3 and S7). 'T1' represents the conventional view: Tarim was at Rodinia's periphery juxtaposed to somewhere of northwestern Australia or India northwestern Australia during most Neoproterozoic time afterwards [55]. However, there are at least three challenges for this connection. First, the Tarim Block shows a more comparable stratigraphic/biological records and tectonic history with North China rather than with the nearby South China during the late Ediacaran-early Paleozoic time. The late Ediacaran glacial deposits are well developed in both Tarim (including Chaidam) and North China, not in South China [64]. Also, the time patterns of accretionary-collisional events in the Tarim Block are comparable to those in North China, which have been related to the assembly of East Gondwana during the early Paleozoic [68]. Second, Tarim needs to experience a complicated kinematics, i.e., a circuitous path around the northern Australia to come together with Chaidam in the subsequent evolution [60, 68]. Third, a long connection of Tarim and northwestern Australia cannot be achieved by fitting their Neoproterozoic-early Paleozoic APWPs as pointed out [69, 70]. In order to solve these mismatches or problems above, an alternative position juxtaposed with North China ('T2') is provided (Fig. S7). In this context, the eastern Tarim (Quruqtagh) is adjacent to both Chaidam and the western part of North China (current coordinates) where the post-SE (≤560 Ma) glaciations preserved (this study) (Figs 1 and 3; Table S1). Moreover, this juxtaposition also can facilitate Tarim's migration from the "missing-link" connection, i.e., between Australia and Laurentia near the center of Rodinia [63, 69, 71] to this configuration through the middle to late Neoproterozoic. It is worth noting that, for these two alternative options, the pole of T-Zma from the Zhamoketi andesites is either coincident with the poles of ~590-580 Ma group or those of ca. 560 Ma (Fig. S7). If a younger age is assigned to this pole, it would give some quantitative constraints on the paleolatitude (~ 30°N/S) of the Tarim block for the great Ediacaran glaciation initiation in this block.

6. Age constraints of Ediacaran glacial deposits in Figure 5

The Ediacaran glacial deposits in different continents (Table S1) are plotted in five paleogeographic maps, i.e., 590-580 Ma, 575 Ma, 570 Ma, 565 Ma and ~560 Ma maps (Figure 5B). The assignments of glacial deposits are based on the available geochronology data (radiometric ages), chemostratigraphic data (e.g., $\delta^{13}C_{carb}$ and $^{87}Sr/^{86}Sr$), biostratigraphic data (fossil records) and regional stratigraphic correlation. In addition, the paleogeographic distribution of glacial deposits should also accord with the non-Snowball Earth condition of the Ediacaran glaciation, i.e., the absence of low latitude (within 30°N/S) glaciations.

Based on the quality of geochronology, chemostratigraphy, and biostratigraphy data, the ages of Ediacaran glacial deposits are variably constrained. For example, the Gaskiers Formation in Avalonia, the Las Vantanas Formation in Rio Plata, and the Tiddiline Formation in West Africa can be unambiguously identified as 590-580 Ma glaciations, while the Dhaiqu Formation in Arabia, the Weesenstein/Clanzschwite/Orellana Formations in Cadomia, the Kahar Formation in Iran, the Hankalchough Formation in Tarim and the Pourpree I'Ahnet, Fersiga formations in West Africa can be classified as ~560 Ma (or <560 Ma) glaciations.

However, some Ediacaran glaciations can be placed into multiple paleogeographic maps based on the available age constraints. In this case, the glacial age can be additionally constrained by the principle of absence of low latitude glaciations in a non-Snowball Earth climatic condition (Fig.S8). For example, glacial deposits in Australia and Baltica are loosely constrained between 590 Ma and 560 Ma by geochronology data. Based on the new paleogeographic maps reconstructed in the context of IITPW event, South Australia and Baltica were placed in the tropical regions in both 590-580 Ma and 560 Ma maps (Figs. S8), suggesting the presence of low latitude glaciations (e.g., the Egan Formation in West Australia, the Billy Springs Formations in South Australia, the Groles Hill in Tasmania and the Moelv/Mortensnes/Vil'chitsy/Kurgashlya/Tanin to Starye Pechi formations in Baltica), which is inconsistent with the non-Snowball Earth climatic condition. Thus, we can exclude these possible duration ranges (i.e., 590-580 Ma, 575 Ma or ~560 Ma, Fig. 5) and identify that the glacial deposits in both South Australia and Baltica were deposited at ~570 Ma (570 Ma and 565 Ma, Fig. 5). In the same way, we can constrain the Gwna Group and the Squantum Formation in Avalonia at 590-580 Ma, limit the Fauquier and the Loch na Cille glaciation in

Laurentia at 590-570 Ma, and identify the Tany-Starya Pechi glacial deposits in Siberia at ~570 Ma. The Hongtiegou Formation in Chaidam and Luoquan Formation in North China can be also classified as ~560 Ma glaciations, which are consistent with fossil records [72].

Finally, there are still some Ediacaran glacial depositions, whose ages remain loosely constrained even with the consideration of paleogeographic reconstruction, including the Serra Azul Formation in Amazonia, the Iporanga Formation in Sao-Francisco, the Bokson diamictite in Siberia and the Pourpree I'Ahnet Formation, Fersiga Formation and Ouarzazate Group in West Africa. These glacial deposits are marked as possible glaciations in all paleogeographic maps in Fig. 5. We include the descriptions of Ediacaran glacial depositions in the following sections.

6.1 Serra Azul Formation in Brazil (Amazonia)

The Serra Azul Formation uncomfortably overlies dolostone of the Araras Group and is overlain by sandstones of the Raizama Formation in the northern Paraguay belt. The Serra Azul Formation, varying in thickness between 250 m and 300 m, is divided into two lithological units, Unit A (the basal unit, diamictite, ~70 m) and Unit B (the upper unit, laminated siltstones, ~200 m). Dropstone, polished and bullet-shaped mudstone clasts, multi-directional striated clasts, and incision surface (paleovalley) argue for a glacial origin of the Serra Azul diamictite in Unit A [73, 74]. The age of the Serra Azul Formation is not well-constrained. Unit A has been correlated with the Gaskiers Glaciation, based on (1) a Pb-Pb carbonate isochron age of 627±37 Ma from the underlying Araras Group, (2) an $^{40}Ar/^{39}Ar$ age of 640±15 Ma from detrital muscovite from the Serra Azul Formation [75, 76], (3) $^{87}Sr/^{86}Sr$ and $^{13}C_{carb}$ chemostratigraphic correlation [76, 77], and (4) an $^{40}Ar/^{39}Ar$ age of 544±7 Ma of detrital muscovite from the upper part of the Alto Paraguay Group [76].

6.2 Dhaiqa Formation in Saudi Arabia (Arabian)

The Dhaiqa Formation conformably overlies the Mataar Formation and uncomfortably underlies the Cambrian Siq Sandstone. Glaciomarine diamictite from the Dhaiqa Formation is composed of poorly sorted polymictic conglomerates with the occurrences of possible dropstone structures. The diamictite is overlain by a carbonate bed, which is composed of algal

boundstone with positive $\delta^{13}C_{carb}$ (~1.2 ‰) values [78]. An ash bed just below the diamictite provides a U–Pb age of 560 ± 4 Ma [79].

6.3 Egan Formation in West Australia

The Egan Formation is the lowermost unit of the Louisa Downs Group in Mt. Ramsay area, and consists of dolostone and limestone intercalated with siliciclastic depositions, including diamictite, conglomerate and sandstone [80]. The diamictite unit (~1-20 m in thickness) overlies the lower carbonate layer, and is composed of a range of sub-rounded to subangular clasts with a reddish dolomitic matrix. The glacial origin of diamictite is supported by polished, striated and faceted cobbles, grooves and chatter marks in diamictite [81, 82]. A carbonate bed immediately overlying diamictite, interpreted as the cap carbonate, has $\delta^{13}C_{carb}$ values of -2~2‰ [82]. There is no direct radiometric age constraint on the Egan Formation. However, it has been regarded as a post-Marinoan or an Ediacaran glacial deposition based on the $\delta^{13}C_{carb}$ values of ~0‰ of the cap carbonate, which are not similar with that of Marinoan cap carbonate [80].

6.4 Billy Springs Formation in South Australia

The Billy Springs Formation in the upper part of the Wilpena Group (~620-550 Ma) is composed of planar-laminated mudstone, convolute-laminated mudstone, tabular-bedded sandstone and rare diamictite (~10 m). Because of the lack of unambiguous glacial indicators (e.g., striated and faceted clasts) and limited stratigraphic and spatial distribution of diamictite, the glacial interpretation of the Billy Springs diamictite remains ambiguous [83, 84]. Although the Billy Spring Formation has no direct radiometric age constraint, the underlying Wonoka Formation yields a strong negative excursion [25] that can be compared with the 571-562 Ma Shuram Excursion [17], suggesting younger than 562 Ma.

6.5 Bunyeroo Formation in South Australia

The Bunyeroo Formation, the lower part of the Ungoolya Group, contains some typical glaciogenic sedimentary structures, such as dropstone, granule clusters, frozen aggregates, and till pellets within mudstones, implying an ice-rafted deposits [85, 86]. Available radiometric age constraints include, a Rb–Sr whole-rock shale isochron age of 609±64 Ma from the

Brachina Formation, a Rb-Sr isochron age of 588 ± 35 Ma from the Yarloo Shale (equivalent to the Bunyeroo Formation) [87], a single zircon U–Pb age of 556±24 Ma from the Bonney Sandstone [88], and an age of ~555 Ma for the Ediacara Member near the base of the Rawnsley Quartzite by compared with the age of taxa of the "White Sea Association" in South Australia [89]. In addition, the upper Wonoka Formation (presents the SE, see above) gave a minimum limit of ~570 Ma [17]. Thus, the Bunyeroo glaciation might be bracketed between ~590 Ma and ~570 Ma.

6.6 Croles Hill diamictite in Tasmania

The Kanunnah subgroup is mainly composed of, in chronological order, the Keppel Creek Formation, the Croles Hill diamictite, and the Spinks Creek volcanics. The Croles Hill Diamictite (up to 250 m thick) is overlain by 10-50 m thick red mudstone, which contains pebble- to boulder-sized clasts with the composition of basalt, sedimentary rocks, and felsic volcanic rocks. Dropstone structures intercalated with laminated siltstone and mudstone indicate an ice-rafted distal glaciomarine deposition [90]. A U–Pb zircon age of 582±4 Ma is obtained from a underlying rhyodacite [90].

6.7 Cottons Breccia of King Island (Australia)

The Cottons Breccia is the basal unit of the Grassy Group in King Island. The Cottons Breccia consists of diamictite (>100 m in thickness) and minor volcaniclastic sandstone. A glacial influenced deposition is supported by unsorted polymictic clasts, the presence of till clasts and dropstone structures in intercalated laminated siltstone [90, 91]. The overlying Cumberland Creek Dolostone (CCD) have $\delta^{13}C_{carb}$ values of -1.9‰ to -5‰ [82]. A 575±3 Ma date from the Grimes Intrusive Suite provide a minimum age constraint on the the Cottons Breccia [90]. Together with negative $\delta^{13}C_{carb}$ value of CCD, possible correlation of the Cottons Breccia with the Marinoan glaciation cannot be ruled out [82].

6.8 Gaskiers Formation in Newfoundland (Avalonia)

The Gaskiers Formation conformably overlies the Mall Bay and underlies the Drook formations of the Conception Group. The Gaskiers Formation is up to 300 m thick and is mainly

composed diamictite with the presence of dropstone structures and striated clasts [92]. A ~50 cm-thick carbonate bed that directly overlies diamictite in the Conception Bay may represent the cap carbonate of Gaskiers glaciation and shows an upward increase of $\delta^{13}C_{carb}$ from -6‰ to -1.5‰ [93]. Deposition of the Gaskiers diamictite on the Avalon Peninsula is precisely dated between 580.90 ± 0.40 and 579.88 ± 0.44 Ma by U-Pb zircon geochronology from four sections [94].

6.9 Gwna Group of Scotland in Avalonia

The Gwna Group underlies the Arfon Group and overlies the Anglesey blueschist belt [95]. Dropstone structures occur in the upper part of the mafic mudstone of the Gwna Group [96]. The Gwna Group is sandwiched between two metamorphic rock units with metamorphic ages of 595±12 Ma (Holland Arms gneiss) and 550-560 Ma (blueschist unit), respectively.

6.10 Squantum Formation/member in the Boston Basin (Avalonia)

The Squantum Formation/member is overlain by the Cambrige Argillite and underlain by the Dorchester member. A glaciogenic interpretation is fundamentally based on the appearance of poor sorted large clasts in a finer matrix [97]. Faintly striated Pebbles and thin horizon of thinly laminated pebbly argillites were identified as 'ice-rafted debris layers' containing dropstone structures. However, recent sedimentological investigation (i.e., the depositional setting of the Squantum 'Tillite' appears to be that of a submarine slope/fan setting in an open marine volcanic arc basin) suggests a non-glacial deep-marine depositional setting free of any direct glacial depositional influence [98]. An age of 595±12 Ma from welded tuff clast would provide a maximum age limitation. Glaciomarine sedimentation was followed by deposition of Cambrige Argillite with a minimum depositional age of ca.570 Ma (or younger) from the Mystic River Quarry ash bed in the Cambridge Argillite [99].

6.11 Mortensnes Formation in northern Norway (Baltica)

The Mortensnes Formation is overlain by mudstone and sandstone of the Stappogiedde Formation, and overlies Nyborg Formation in the Vestertana Group. The Mortensnes Formation (<50m) is divided into three members: (1) the lower member consisting of grey-green to purple

massive diamictite, (2) the middle member that can be divided to two sub-members composed of stratified diamictite, (3) the upper member composed of dark grey massive diamictite [100-103]. No high-resolution geochronological constraints are available. Rb-Sr ages of shales associated with the glaciogenic strata broadly provides broad constraints for the Mortensnes Formation between 630 Ma and 560 Ma [104]. Furthermore, Ediacaran fossils are reported above the Mortensnes Formation [101].

6.12 Moelv Formation in southeastern Norway (Baltica)

The Moelv Tillite (<30 m) of the Hedmark Group consists of glacial diamictite and glaciomarine laminated shale with dropstone structures [105]. Paired U–Pb and Lu–Hf ages of detrital zircons from the Rendalen Formation that underlies the Moelv Tillite have identified a youngest detrital zircons age of 616 ± 3 Ma, confirming an Ediacaran glaciation of the Moelv Tillite [105]. On the basis of sequence stratigraphy, the Moelv Formation in the Hedmark Group is generally correlated with the Mortensnes Formation (<560 Ma) [105].

6.13 Vil'chitsy Group in Eastern Europe (Baltica)

The Vil’chitsy Group in Belarus consists of two units, i.e., the lower Blon’ Formation unconformably overlying the upper Glussk Formation. The Blon’ Formation is composed of glacial sediments at the base and interglacial sandstones and sandy dolomites in its upper part. In the most complete sections, the Glussk Formation also includes three diamictite members separated by varved clays [102, 106]. A U-Pb zircon of ~555 Ma from Ust‘Pinega provides a maximum age limit [74].

6.14 Kurgashlya Formation in South Ural (Baltica)

The lower Member of the Kurgashlya Formation consists of an alternation of diamictite, dolostone and laminated siltstones with dropstone structures [102, 107]. There is no radiometric age constraint of the Kurgashlya Formation, yet it has been correlated with the ‘lower Vendian glaciation’[102].

6.15 Tanin(Tany), Koiva and Lower Starye Pechi Subformation in Middle and North Ural (Baltica)

Glacial facies are recorded within the Tany and Koiva formations, which are correlated with the lower and middle parts of the Serebryanka Group and with the lower member of the Staye Pechi Formation of the basal Sylvitsa Group. The Tany Formation (450–500 m in thickness) is composed of massive diamictites separated by sandstones and shales with dropstone structures. The Koiva Formation (250–600 m in thickness) comprises variegated clayey and silty–clayey shales, siltstones, variegated limestone, and dolostone. The Starye Pechi Formation (500 m in thickness) is largely represented by yellowish–greenish gray thin-bedded siltstone and variegated mudstone. Its lower part encloses massive and bedded diamictites intercalated by subordinate shales with dropstone structures. Diamictites of the Tany, Koiva, and Starye Pechi formations may represent distal facies of shelf glaciers and ice rafting deposition [107-109]. These glacial depositions are bracketed by a maximum age of 598.1±6 (zircons, alkali basalts from the basal part of the Tany Formation) [108] and a minimum age of 567.2±3.9 Ma (Zircons, volcanic tuffs in the Perevalok Formation) [110]

6.16 Weesenstein/Clanzschwite/Orellana diamictites in Cadomia Block

Ediacaran glacial deposits occur in the Weesenstein Group, the Clanzschwitz Group and in the Lower Alcudian Group. The Weesenstein Group consists of the older Seidewitz Formation and the younger Müglitz Formation, in which diamictite mainly occurs in the Müglitz Formation of Müglitz Valley. The metasedimentary Clanzschwitz Group is subdivided into three un-named lithological members. The youngest Member 3 is composed of diamictite, greywackes and conglomerates. Both the Weesenstein and Clanzschwite diamictites contain dropstones, flatiron-shaped pebble, and facetted pebbles. The lower Alcudian Group is characterized by a monotonous succession of turbidites composed of greywacke and mudstone, in which the ‘Orellana matrix-supported conglomerate formation (pebbly mudstones)’ is identified as the ‘Orellana diamictite’ [111].

Weesenstein diamictite: The maximum depositional age of 562±5 Ma (U-Pb detrital zircon in Purpurberg quartzite member) or 564±5 Ma (quartz wacke sample in Müglitz Formation), and the minimum age of 538±2 Ma (the age of intrusion of the Dohna granodiorite)

Clanzschwitz diamictite: The maximum depositional age of 570±8 Ma (U-Pb detrital zircon), and the minimum age of 537±5 Ma (The intrusion age of the Laas granodiorite)

Orellana diamictite: The maximum depositional age of 565 ± 4 Ma (U-Pb detrital zircon)

6.17 Hongtiegou Formation in Chaidam (North China)

The Neoproterozoic Quanji Group in the Chaidam Basin, northwest China, consists of, in ascending order, the Mahuanggou, Kubaimu, Shiyingliang, Hongzaoshan, Heitupo, Hongtiegou, and Zhoujieshan formations. The Hongtiegou Formation (~18-75 m thick) consists of green, yellow, and purplish-red massive diamictite. Dropstone structures and striated clasts are widely distributed in the Hongtiegou Formation, indicating a glacial origin [112]. In addition, the Hongtiegou glacial deposit is overlain by a ~5 m thick silty dolostone, which is identified as the 'cap carbonate' of the Hongtiegou glaciation. The Hongtiegou cap carbonate has positive $\delta^{13}C_{carb}$ of 0-2‰ [112, 113]. *Charnia* and *Shaanxilithes* from the Quanjishan assemblage likely constrains the depositional age of the Zhoujieshan Formation to be terminal Ediacaran (ca. 550–539 Ma) and the immediately underlying Hongtiegou diamictites to be late Ediacaran, probably representing post-Gaskiers glacial deposition [65, 72].

6.18 Kahar Formation in Iran (Iran)

Distinct diamictite–carbonate intervals occur in the basal Kahar Formation at Kahar Mountain and Saebandan sections. Clasts in diamictite range from gravel to boulder in size, angular to sub-rounded in shape, and are largely matrix-support. Diamictite matrix consists mainly of fine-grained quartz, mica, and feldspar, with minor diagenetic dolomite [114-116]. A dark gray carbonate layer of ~17.5 m thick abruptly overlies the diamictite layer, probably representing the cap carbonate. And the basal carbonate is brecciated. This 'Cap carbonate' has C isotope values, ranging from -1‰ to +0.5‰ in the Kahar Mountain section and displaying a decreasing trend from -0.2‰ to -4.5‰ in the Sarbandan section [117]. Detrital zircon U–Pb ages of samples from the underlying and overlying strata of the diamictite unit indicate maximum depositional ages of about 560–550

Ma, which is consistent with available $^{87}Sr/^{86}Sr$ and $^{13}C_{carb}$ chemostratigraphic data in Kahar Formation [115, 116].

6.19 Numees Formation in Namibia (Kalahari)

The Numees glacial deposit unconformity overlies the carbonate of the Dabie River Formation and underlies the dolostone of the Bloeddrif Member in the basal of Holgat Formation. ~100 m-thick carbonate of Bloeddrif Member overlies the Numees diamictite, and might represent a cap carbonate of the Numees glaciation. This 'cap carbonate' has $\delta^{13}C_{carb}$ values ranging from -4.6‰ to +1‰, and has a carbonate Pb-Pb age of 555±28 Ma [118, 119]. Thus, the Numees glaciation might be late Ediacaran in age.

6.20 VGU in Namibia

The Nama Group, in ascending order, consists of Kuibis, Schwarzrand and Fish River Subgroups. Erosional surfaces within the late Ediacaran upper Kuibis and lower Schwarzrand Subgroups of the Nama Group are interpreted as the Vingerbreek Unconformity (VGU). VGU are filled with the diamictite and sandstone. This unconformity is considered to form by glacial, proglacial and fluvial erosion. The palaeochannels (palaeovalleys) associated with this unconformity in southern Namibia and northwestern South Africa are interpreted to have formed distant from major glaciers as a result of predominantly glacio-eustatic sea level fall [115]. The glacial deposition was dated between 549±1 Ma (U-Pb age of Zaris Formation) [114]. The occurrence of the fossil *Cloudina* in the calcareous Mooifontein Member indicates that the Vingerbreek Unconformity is younger than 560(?) Ma [120].

6.21 Fauquier Formation in northern Virginia (Laurentia)

Diamictite in the basal Fauquier Formation contains both rounded and angular cobbles and boulders in coarse, cross-bedded sandstone, which are interpreted as pro-glacial fluvial outwash facies. A U–Pb TIMS zircon age of 571±1 Ma from felsic tuffs intercalated in Catoctin Formation, which is ~50 m above the Fauquier Formation [121, 122].

6.22 Inishowen - Loch na Cille beds in British-Irish (Laurentia)

The Inishowen-loch na Cille beds are the Lower unit of Southern Highland Group, which consists of ca. 600 m sandstone interbedded with three horizons of thinly laminated pelagic mudstone with dropstone structures [123]. An ash bed in the Southern Highlands Group yields a U-Pb zircon age of 601 ± 4 Ma [124]. A ca. 595 Ma age of extrusive keratophyre is obtained ~450–500 m [125] above the Tayvallich Limestone (bottom of Southern Highland Group) thereby places the Inishowen ice-rafted debris in the ca. 590–570 Ma.

6.23 Luoquan/Zhengmuguan Formation in North China

The Luoquan Formation unconformably rests upon various units, the youngest of which is the Dongjia Formation, and is conformably overlain by the Dong Po Formation [126]. The Luoquan Formation is considered as a glacial sedimentary sequence partially reworked by sediment gravity flow. In addition, Luoquan glaciation has no cap carbonate, while the Zhengmuguan glaciation has the cap carbonate with -2~-5‰ in carbon isotopes [127]. Some characteristics of sediment gravity flow existing in the Luoquan Formation diamictites indicate that glacial deposits might have been partially reworked by sediment gravity flow [128, 129]. The *Trachysphaeridium, Taeniatum, Lophosphaeridium* and *Tasmanites* from the Luoquan Formation were also reported. The latter two are elsewhere found in association with the *Asteridium-Comasphaeridium* assemblage and linked to the Ediacaran-Cambrian transition [129].

6.24 Las Ventanas Formation in Uruguay (Rio Plata)

The Las Ventanas Formation in Uruguay can be divided into three lithological members: in chronological order, La Rinconada Member, Quebrada de Viera Member, and El Perdido Member. Diamictite (with a maximum thickness of 3.8 m) occurs in the Quebrada de Viera Member, and is composed of polymictic and mostly clast-supported conglomerates, grading to conglomerate-sandstone intercalations. Dropstone structures in laminated and ferruginous siltstone may also suggest the glacial origin [130]. Basalt of the La Rinconada Member (lower Las Ventanas Formation) yields a K–Ar age of 615±30 Ma [131]. In addition, it is proposed that the Las Ventanas Formation is probably older than the Pan de Azúcar Pluton (579±1.5 Ma)

[132, 133]. Thus, an early Ediacaran age between 615 and 579 Ma of the Las Ventanas Formation has been proposed.

6.25 Iporanga Formation in Southern Brazil (Sao-Francisco)

The Iporanga Formation is composed of polymictic unsorted breccia and conglomerate within pelitic matrix, intercalated with rhythmic pelite, sandstone, arkose, and conglomerate within arkosic matrix [134]. A radiometric age of 579±31 Ma was obtained from a volcanoclastic/volcanic rock below the Iporanga Formation, and a 593±15 Ma age is from granitic clast included in the basal Iporanga Formation [135].

6.26 Bokson diamictite of Zabit Formation (Siberia)

Widespread (Bokson) diamictite occurs in the lower part of the Zabit Formation. Diamictite consists of erratic, faceted, and striated stones along with dropstone structures in thin-bedded shales [136]. The deposits underlying the Bokson diamictite contain typical Ediacaran microfossils. Thus, the diamictite has been assigned as late Ediacaran to lowermost Cambrian (Upper Vendian) glacial deposition [137].

6.27 Baykonur Formation (Siberia)

Diamictite of the Baykonur Formation consists of erratic and striated clasts, and is intercalated with shale beds containing dropstone structures [109, 138, 139]. A carbonate layer, presumably representing the 'cap carbonate', is discovered at the top of Baykonur Formation [140]. No high-resolution geochronological age is available.

6.28 Hankalchough Formation (Tarim)

The Neoproterozoic Quruqtagh Group in eastern Chinese Tianshan consists of, in ascending order, the Bayisi, Zhaobishan, Altungol, Tereeken, Zhamoketi, Yukkengol, Shuiquan, and Hankalchough Formations. The Hankalchough Formation (~400 m in thickness) is composed of gray diamictites with diverse erratic and striated stones, and rhythmically layered shales with dropstone structures. The Hankalchough diamictites are overlain by the cap dolostone (1–5 m thick) with negative $^{13}C_{carb}$ values from –4‰ to – 15‰ [2]. The underlying

Shuiquan Formation contains fossil algae *Vendotaenia* sp. [141, 142] and the characteristic organic remains of the Upper Vendian [143]. A U-Pb zircon age of 615 ± 6 Ma from the Zhamoketi Formation suggests the Ediacaran glacial deposition of the Hankalchough Formation [3].

6.29 Tiddiline Group et al. in Mococco (West Africa)

In the eastern and central Anti-Atlas, diamictites are reported from six sections: the Tiddiline Group in the Ait Ahmane and Bou Azzer sections, the Saghro Group in the Tizwat section, the Izdar Member in the Taghdout, Timzioura, Taghdout, and Ansra sections. Pebble cluster and dropstone structures within finely laminated sandy siltstone, are interpreted as the evidence of an ice rafting deposition [144]. A TIMS U-Pb detrital zircon age provides a maximum limit at 592.31 ± 0.18 Ma, while the age of a barely deformed intrusion from Bou Azzer area could be taken as a younger age constraint of 579.4 ± 1.2 Ma (multigrain zircon TIMS data) [145].

6.30 Ouarzazate Group in Morocco (West Africa)

The Quarzazate Group are composed of volcaniclastic deposits interbedded with conglomerate and glacially-derived breccia. In the Quarzazate group, erosional features are commonly interpreted as resulting from ice-sheet movement on a soft-sediment substratum, including striated and fluted surfaces. The stratified breccia is considered as a short-term pro-glacial deposition, reflecting a high-frequency melting [146]. The Bou-Azzer tillite of the Ouarzazarte Group is below an ignimbrite dated at 561 ± 9.5 Ma [147], and above an ignimbrite dated at 565 ± 6 Ma [148]. Compared with other glacial deposits in West Africa, the Bou-Azzer glaciation might be constrained between 565 Ma and 550 Ma [146].

6.31 Pourpree de l'Ahnet Group (West Africa)

the Pourpree de l'Ahnet Group in northwestern Hoggar Highland is composed of diamictite, carbonate, and chert-tuff members. The diamictite consists of erratic, faceted, and striated stones and dropstone structures [138]. The triad of the Pourpree de l'Ahnet Group unconformably overlies granites dated at 560±10 Ma (U-Pb and Rb-Sr methods) [149]. The U-

Pb and Ar-Ar dating of tuff beds from the triad upper member range from 523 to 535 Ma [150]. Accordingly, glacial deposits of the Pourpree de l'Ahnet Group might be of the Late Ediacaran to Early Cambrian age.

6.32 Fersiga Group (West Africa)

The Fersiga Group consists of continental tillites, glacio-lacustrine and glacial-fluvial sediments, and aeolian depositions. In Fersiga, the tillite glacial morphology consists of unconsolidated north-south–oriented ridges (50 m high and 3–4 km wide) with steep slopes facing east, interpreted as terminal moraines of a retreating glacier [151]. The age of the Fersiga Group is not well-constrained. A Rb-Sr isochron age of whole rocks and a U-Pb zircon age give a large range of uncertainty, ranging from 556±12 Ma to 519±11 Ma, suggesting a late Ediacaran to early Cambrian age. The radiometric age constraints are consistent with fossil record [151].

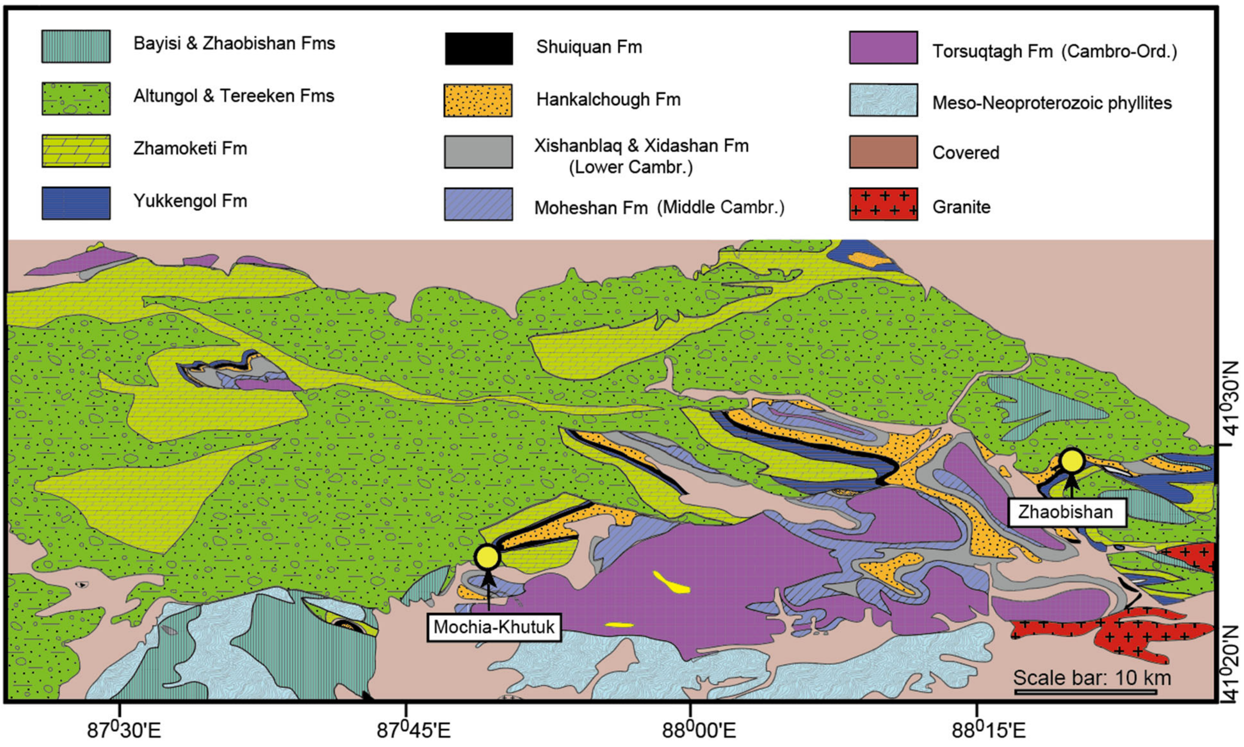

Fig. S1. Geological map of the Quruqtagh area in the Tarim Block, northwestern China. The studied sections, Mochia-Khutuk (MK) and Heishan-Zhaobishan (HZ), are marked with yellow circles.

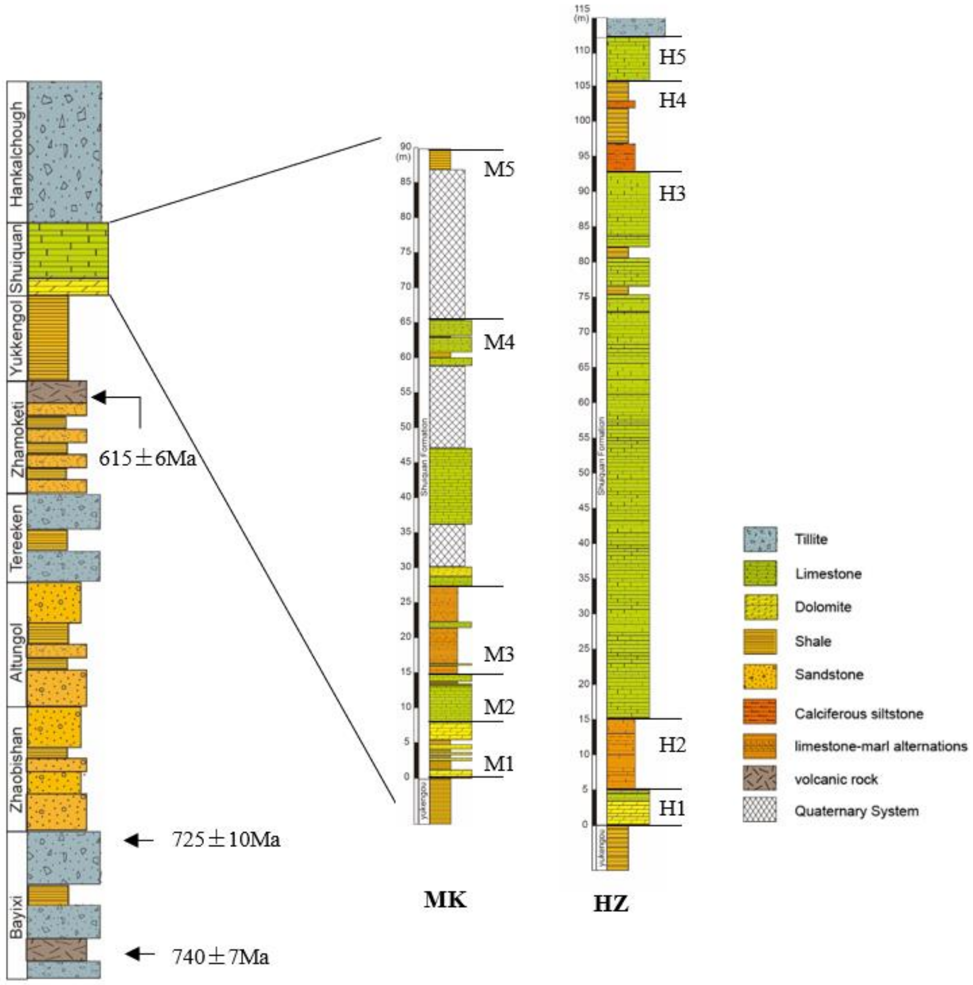

Fig. S2. The lithostratigraphy of the Neoproterozoic Quruqtagh Group and the lithostratigraphic column of the Shuiquan Formation in the Mochia-Khutuk (MK) and the Heishan-Zhaobishan (HZ) sections. The Beiyixi glaciation was dated between 740 ± 7 Ma and 725 ± 10 Ma, and the Zhamoketi Formation was dated in 615 ± 6 Ma [3].

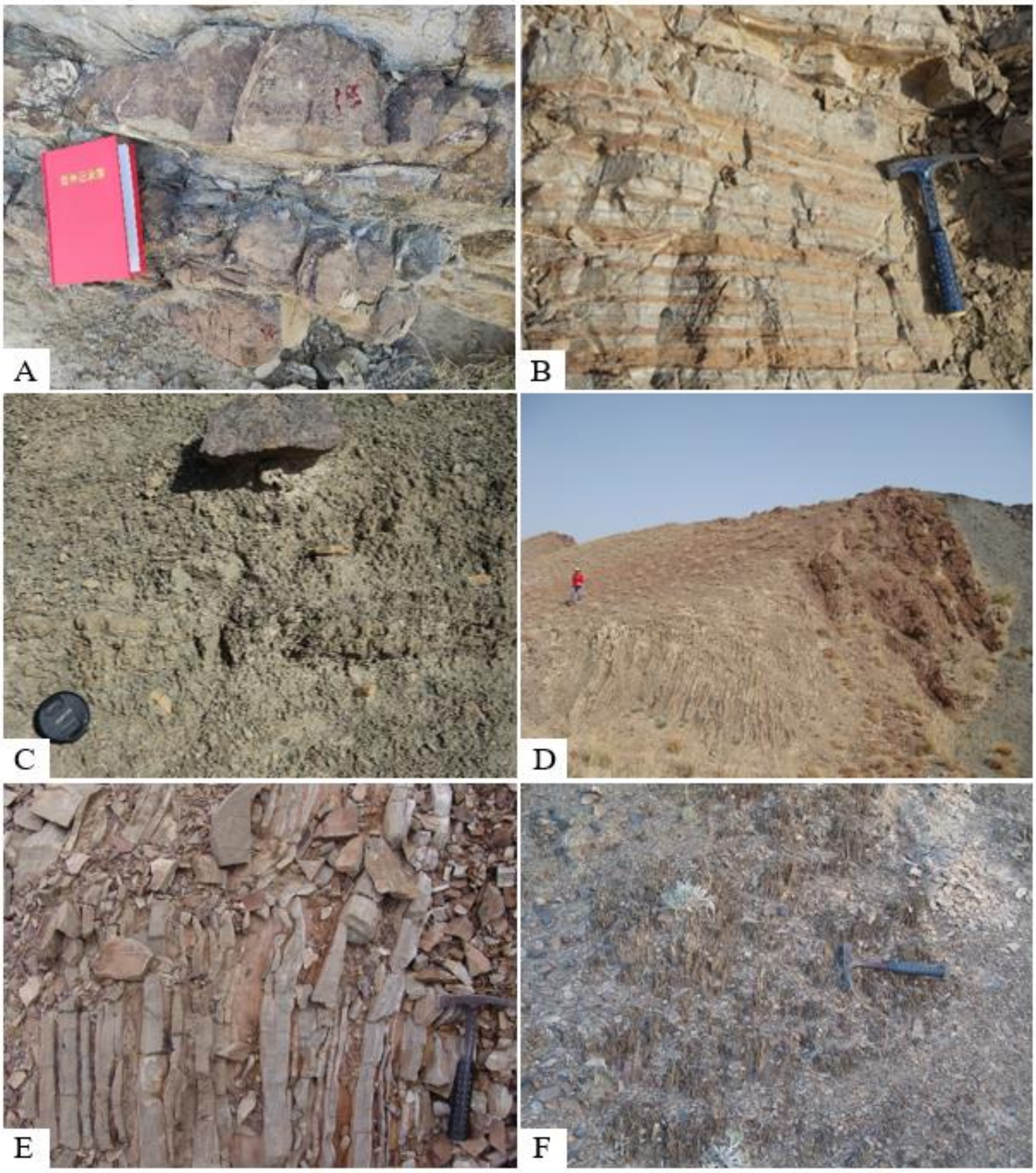

Fig. S3. Field photographs of the Shuiquan and Hankalchough formations in the Quruqtagh area, Tarim Block. A: Reddish thin- to medium-bedded coarse crystalline dolostone in Shuiquan Formation (M2 in MK), B: Limestone-marl alternations in Shuiquan Formation (M3 in MK), C: Calcareous siltstone in the top of Shuiquan Formation (M5 in MK). D: The contact between the Shuiquan and Yukengou formation in HZ. The dolostone of the basal Shuiquan Formation overlies the dark gray siltstone/mudstone of the top of Yukengou Formation. E: Reddish-brown dolostone and light-gray limestone layers on the bottom of Shuiquan Formation (H1 in HZ). F: shale-calcareous siltstone in the top of Shuiquan Formation (H4 in HZ).

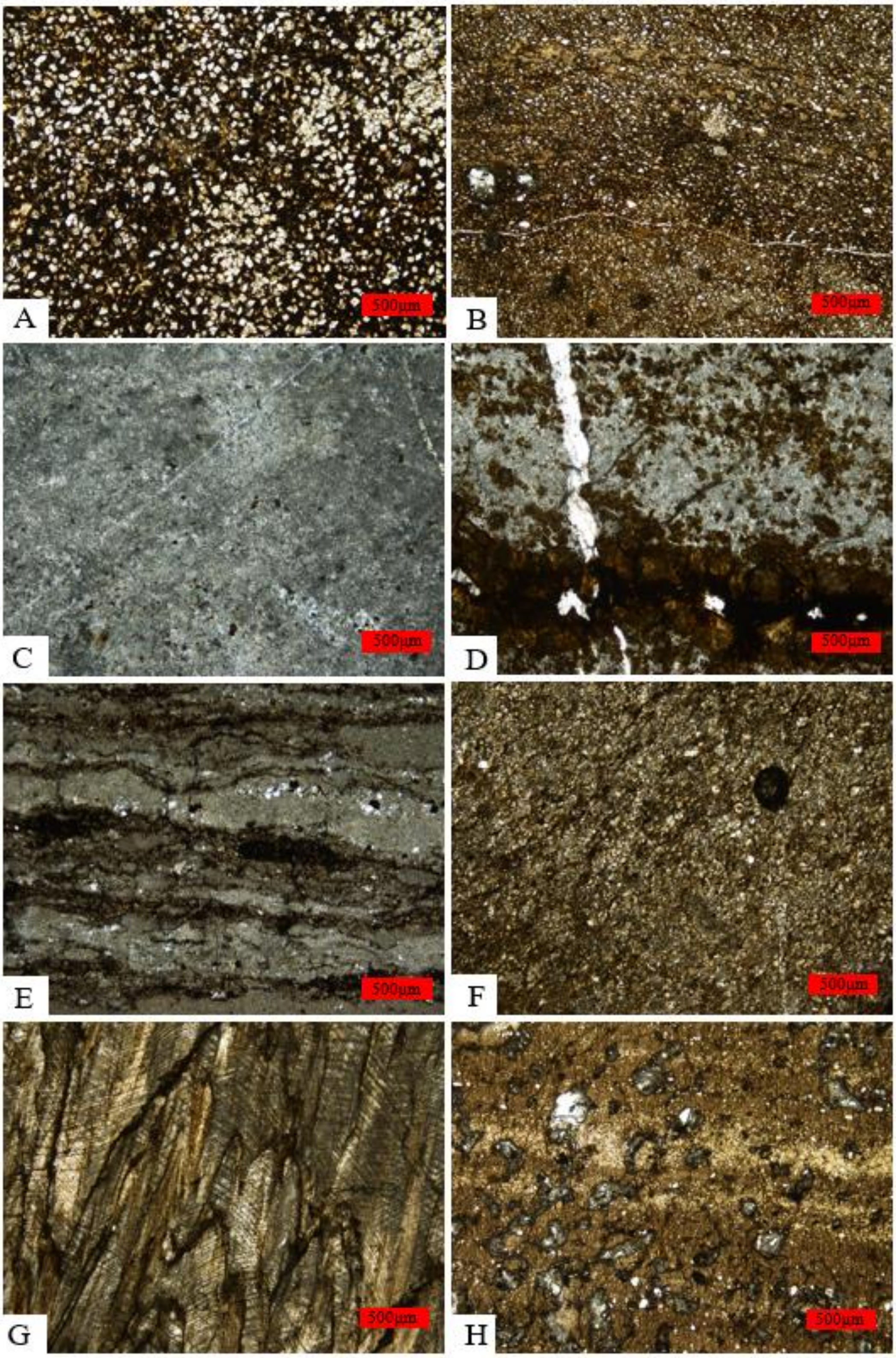

Fig. S4. Photomicrographs of carbonate from the Shuiquan Formation in MK section. (A) Coarse crystalline dolostone in U1. (B) Microcrystalline limestone in U1. (C) Micrite in U1 and U2. (D) Recrystallization of dolostone in U3. (E) laminated limestone in U2 and U3. (F)

Microcrystalline limestone in U4. (G) Crystal fan of limestone in U4. (H) Recrystallization of dolostone in U4.

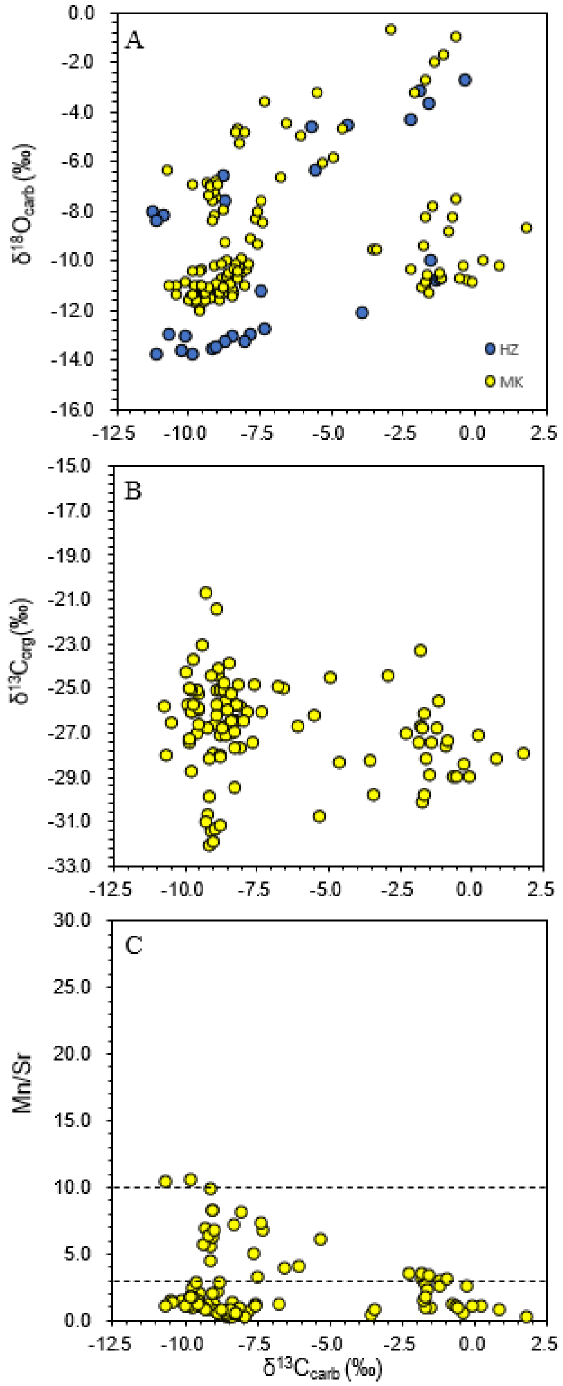

Fig. S5. The element and isotope cross-plots. (A) $\delta^{13}C_{carb}$ vs. $\delta^{18}O$, (B) $\delta^{13}C_{carb}$ vs. $\delta^{13}C_{org}$, (C) $\delta^{13}C_{carb}$ vs. Mn/Sr ratios.

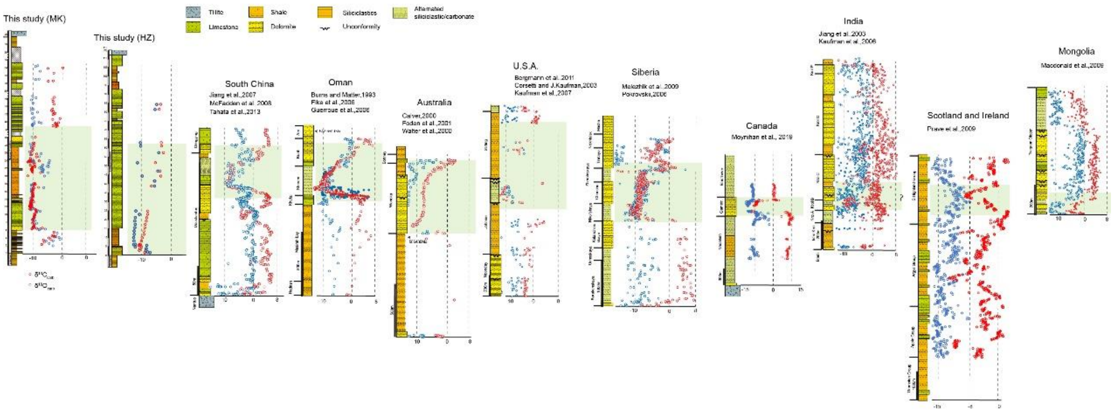

Fig. S6. The global chemostratigraphic correlations of the Shuram excursion between different localities.

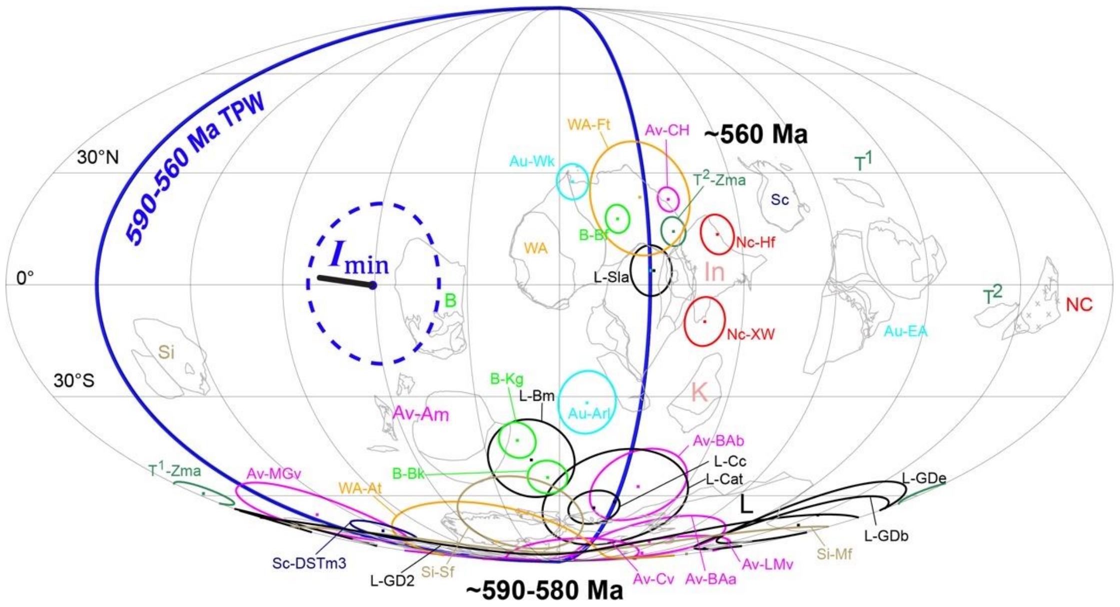

Fig. S7. Ca. 590-560 Ma TPW-portion great circle and its orthogonal axis I_{min} with 95% confidence ellipse in an absolute reference frame [45]. Key points for the reconstruction are described in the Supplementary text. The pole list and abbreviations are shown in [45]. Parameters of relative rotations for different continents are summarized in Table S5. Note: two optional positions ('*T1'*, '*T2'*) for the Tarim Block are shown. NC-North China, T-Tarim, CA-Chaidam, Au-Australia, EA- East Antarctica, WA-West Africa, NA-Northeast Africa, I-India, SC-South China, SFC-Sao Francisco-Congo, RP-Rio Plata, K-Kalahari, B-Baltica, Am-Amazonia, Av-Avalonia, Si- Siberia, L-Laurentia.

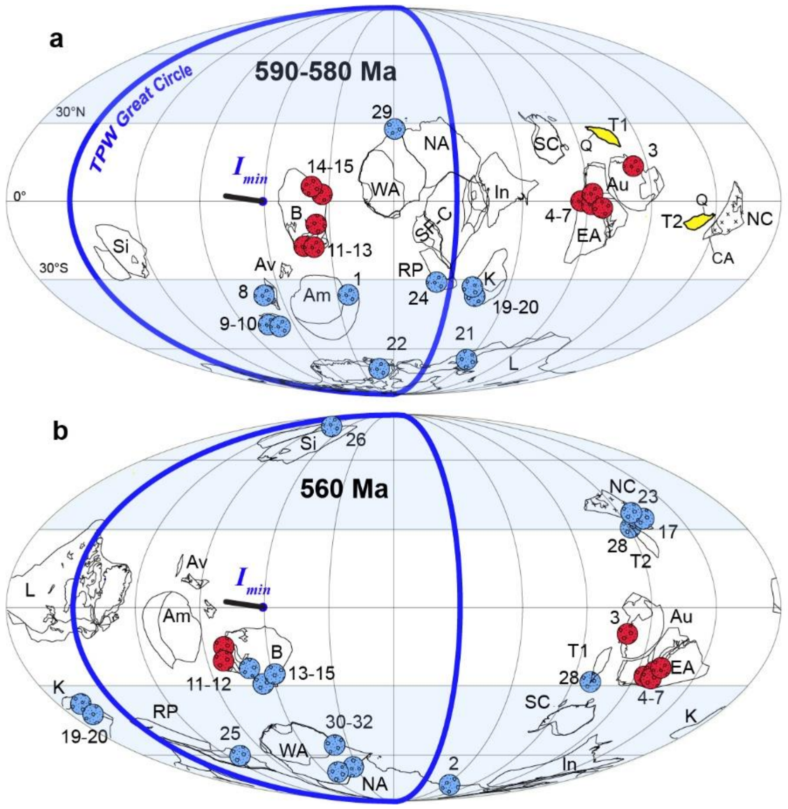

Fig. S8. The possible geographic distributions of Ediacaran glacial deposits based on poor age constraints. Under a non-Snowball Earth climatic condition, the low-latitude Ediacaran glacial deposits are unlikely, marked as red circles. Meanwhile, the presence of low-latitude glaciations in these two intervals (a and b) argues against two separate non-Snowball Earth glaciations (i.e., ~580 Ma and ~560 Ma).

Table S1. Duration of Ediacaran glaciation

No.	Palaeocontinent	Formation	Age	Succession/Basin	Country	Reference	Notes
1	Amazonia	Serra Azul Fm.	<630 Ma	Northern Paraguay belt	Brazil	Alvarenga et al., 2007; McGee et al., 2013, 2015;Babinski et al. 2006	
2	Arabia	Dhaiqa Fm.	<560 Ma		Saudi Arabia	Vickers-Rich and Fedonkin, 2007; Miller et al., 2008	An ash bed just below the diamictite provided a U–Pb age of 560±4 Ma .
3	Australia	Egan Fm.	?	Kimberley region	Northwestern Australia	Corkeron and George, 2001; Corkeron, 2007; 2011	"Cap carbonates" is not similar with Marinoan, which has higher carbon isotopes (-2~2‰) ; Stratigraphic correlation: post-marinoan.
4		Billy Springs Fm.	< 562 Ma	Adelaide Basin	South Australia	Jenkins, 2011; Counts and Amos., 2016	
5		Bunyeroo Fm.	590-570 Ma	Adelaide Basin	South Australia	Gostin et al., 2010	A Rb–Sr isochron age of 588 +- 35 Ma for the Yarloo Shale on the Stuart Shelf, equivalent to the Bunyeroo Formation. the basal part of the Wonoka Formation, and provided a revised mean age of 593 +-32 Ma for the Bunyeroo Formation. <10 m
6		Croles Hill diamictite	<582 Ma	Kanunnah subgroup	Tasmania	Calver et al., 2004	U-Pb zircon, <582±4 Ma
7		Cottons Breccia	>575 Ma	Grassy Group	King Island	Walter et al., 2000; Calver et al., 2004; Direen and Jago, 2008; Corkeron, 2011	U-Pb zircon, >575±3 Ma ; ca.600 Ma or between 586 and 572 Ma (Direen and Jago, 2008). Stratigraphic correlation with Tasmania?
8	Avalonia	Gaskiers Fm.	~580 Ma	eastern Newfoundland	Canada	Eyles et al., 1989; Pu et al., 2016	Tuff beds, between 580.90 ± 0.40 and 579.88 ± 0.44 Ma; Carbon isotopes of "Cap carbonate" is -6‰ ~ -2‰ in Harbour Main section (50cm), and -8‰~-5‰ in Kitchuses section (20cm)
9		Gwna Group	595-550 Ma	Anglesey Island (Llanddwyn island), Wales	UK,Scotland	Kawai et al., 2008	Gwna Group is sandwiched between two types of metamorphic rocks that have metamorohic ages of Rb–Sr whole rock isochron age of 595 ±12 Ma, and 550-560 Ma. Ice-rafted dropstones
10		Squantum Fm.	595-570 Ma	Boston Basin	USA	Sayles, 1914; Thompson and Bowring, 2000; Carto, 2012	U-Pb tuff clast from Squantum diamictite; 595.5±2 Ma. U-Pb ash bed; ~570 Ma or younger?

11	Baltica	Mortensnes Fm.	630-560 Ma	Verstertana	North Norway	Farmer et al., 1992; Chumakov, 2007; Rice et al., 2011	
12		Moelv Fm.	620~560 Ma		South Norway	Bingen et al., 2005	U-Pb and Lu-Hf detrital zircons; correlation
13		Vil'chitsy Group	>555 Ma	Eastern Europe	Eastern Europe (Belarus)	Chumakov, 2004, 2007; McGee et al., 2013	U-Pb zircon Ust'Pinega?
14		Kurgashlya Fm.	?	Belaya River	South Ural	Chumakov, 1998 and 2007	"lower Vendian"?
15		Tany, Koiva and Lower Starye Pechi formations	598-567 Ma	North and Middle Urals	Ural	Chumakov, 2011; Maslov et al., 2013; Grazhdankin et al., 2011	U-Pb zircons SHRIMP, volcanic tuffs, between 598.1±6 Ma (Zircons, LA-ICP-MS, alkali basalts) and 567.2±3.9 Ma (Zircons, LA-ICP-MS, volcanic tuffs)
16	Cadomia	Weesenstein/Clanzschwite/Orellana diamictite	565-540 Ma	Cadomian orogen	NE Bohemian massif and SW Iberia	Linnemann et al., 2018	
17	Chaidam	Hongtiegou Fm.	~560 Ma?		China	Shen et al., 2007, 2010; Pang et al., 2021	Fossils (>547 Ma?); 'Cap carbonate' has higher carbon isotopes with 0-2‰
18	Irania	Kahar Fm.	<563 Ma	Central Alborz Mountains (Kahar Mountain and Saebandan section)	Northern Iran	Etemad-Saeed et al., 2016	U–Pb detrital zircons (<563.1±3.9 Ma) below the Kahar Fm
19	Kalahari	Numees Fm.	>555 Ma	Namibia	Namibia, South Africa	Frimmel et al.,2002; Fölling et al., 2002	‘cap carbonate’ has a carbonate Pb-Pb age of 555±28 Ma
20		VGU	>550 Ma	Nama Foreland Basin	Namibia	Grotzinger et al., 1995; Germs and Gaucher, 2012;	A U-Pb zircon age of 549±1 Ma from the underlying Zaris Formation further implies the VGU is at least younger than 550 Ma
21	Laurentia	Fauquier Fm.	>571 Ma	Northern Virginia	USA	Southworth et al., 2009; Hebert et al., 2010	U-Pb TIMS zircon analyses of felsic tuffs intercalated with meta-basalt flows within 50m of the base of the formation provide an age of 571±1 Ma.
22		Inishowan-Lochna Cille beds	590-570 Ma	Dalradian surpergroup	British-Irish	McCay et al., 2006; Chumakov, 2007;Dempster et al., 2002	

23	North China	Luoquan/Zhengmuguan	~560 Ma?	Qinling	China	Guan et al., 1986; Wu and Guan, 2009; Heron et al., 2018; Yang et al., 2019	Fossils; Carbon isotopes of 'Cap carbonate' are -2~-5‰
24	Rio Plata	Las Ventanas Fm.	> 579 Ma	Western Gondwana	Uruguay	Gaucher et al., 2008, Oyhantcabal et al., 2006; Sanchez Bettucci and Linares, 1996; Blanco and Gaucher, 2005	K-Ar Basalt (615±30 Ma); Ar-Ar post-collisional plutons(579± 1.5 Ma)
25	Sao-Francisco	Iporanga Fm	<580 Ma	São Francisco	Southeast Brazil	Perdoncini and Soares, 1992; Campanha et al., 2008	U-Pb zircon SHRIMP ~580Ma is assigned for the base of this units
26	Siberia	Bokson diamictite (Zabit Fm.)	~560 Ma	South of the East Sayan Mountains	Mongolia;Buryatian Republic	Osokin and Tyzhinov, 1998; Chumakov, 2011	Stratigraphic position;biostratigraphic data; Fossils: the Upper Vendian *Cloudina* sp. have been found in the diamictite matrix; 'Cap carbonate' is 7.5-10m in thickness.
27		Baykonur Fm.	~560 Ma	Ulutau Mountains of central Kazakhatan	Kazakhstan/Kyrgyzstan,Asia	Chumakov et al., 2009 and 2011	Correlation? Baykonurian Glaciation
28	Tarim	Hankalchough	<562 Ma	Quruqtagh	China	Xiao et al., 2004; Xu et al., 2009; Shen et al., 2011	this study, younger than SE ; Carbon isotopes of 'Cap carbonate' are various (-4 ~ -15‰)
29	West Africa	Tiddiline Group et al.	592-579 Ma	Anti-Atlas	Morocco	Letsch et al., 2018; Inglis et al., 2005	U-Pb zircon, between 592.31 ± 0.18 Ma and 579.4 ± 1.2 Ma; Anti-Atlas glaciation
30		Ouarzazate Group	<565 Ma	Anti-Atlas	Morocco	Vernhet et al., 2012; Karaoui et al., 2015; Blein et.al., 2014； Letsch et al., 2019	Bou-Azzer glaciations, 565-550 Ma ;The Bou-Azzer tillite of the Ouarzazarte Group of Morocco is below an ignimbrite dated at 561 ± 9.5 Ma, and above an ignimbrite dated at 565 ± 6 Ma.
31		Pourpree l' Ahnet Group	560-530 Ma	Northwest Hoggar Highland	Algeria	Chumakov, 2009; Caby and Fabre, 1981; Deynoux et al., 2006	560+-10 Ma (U-Pb and Rb-Sr methods, granites) to 530 Ma (U-Pb and Ar-Ar data, tuffs from the upper member); 'Cap carbonate' has been reported
32		Fersiga Group	556-519 Ma	Northwest Ahaggar and south of the Taoudenni basin	Algeria	Bertrand-Sarfati et al., 1995	Early Cambrian glaciation? (Fossils); 519±11 Ma (Rb/Sr isochron on whole rocks); 556+-12 Ma (U/Pb on zircon)

Table S2. Carbonate Carbon ($\delta^{13}C_{carb}$), Oxygen Isotopes($\delta^{18}O$) and Organic Carbon Isotopes ($\delta^{13}C_{org}$) of Shuiquan Formation, MK section, South China.

Formation	Samples	Lithology	Height (m)	Carb. Content (%)	Mg/Ca	Fe/carb (ppm)	Mn/carb (ppm)	Al/carb (ppm)	Fe/Mn	Al/Mg	Mn/Sr	$\delta^{13}C_{carb}$ (‰)	$\delta^{18}O_{carb}$ (‰)	$\delta^{13}C_{org}$ (‰)	△carb-org (‰)
Shuiquan	19XE-SQ-51	black calcareous siltstone	95.00	2.11	0.435	250647.33	1553.60	1.29	161.33	3.23	1.64				
Shuiquan	19XE-SQ-50	black calcareous siltstone	93.00	0.69	0.592	4173991.12	8505.81	0.26	490.72	19.24	3.27				
Shuiquan	19XE-SQ-49	black calcareous siltstone	91.00	0.91	0.411	3063087.48	6537.56	1.51	468.54	21.19	2.16				
Shuiquan	19XE-SQ-48	black calcareous siltstone	89.00	1.56	0.141	1237743.50	3134.32	1.92	394.90	26.01	1.15				
Shuiquan	19XE-SQ-47	interbedded lime mudstone	64.80	90.57	0.001	8221.82	448.44	914.87	18.33	4.57	0.35	1.82	-8.64	-27.94	29.76
Shuiquan	19XE-SQ-46	interbedded lime mudstone	64.60	95.68	0.001	9490.41	558.26	1221.62	17.00	5.28	0.47	-3.56	-9.49	-28.28	24.71
Shuiquan	19XE-SQ-45	interbedded lime mudstone	63.30	88.37	0.002	6859.93	1038.16	2537.74	6.61	6.39	1.03	-1.49	-7.81	-28.95	27.46
Shuiquan	19XE-SQ-44	interbedded lime mudstone	61.60		0.002	3386.28	716.18	2175.00	4.73	5.17	0.99				
Shuiquan	19XE-SQ-43	interbedded lime mudstone	61.10	74.65	0.002	5329.69	918.46	2949.60	5.80	7.49	1.00	-1.71	-8.22	-30.11	28.39
Shuiquan	19XE-SQ-42	interbedded lime mudstone	61.00	85.72	0.002	3596.71	630.52	1988.20	5.70	4.04	0.84	-3.45	-9.49	-29.79	26.35
Shuiquan	19XE-SQ-41	thin-bedded limestone	46.47	60.32	0.004	22985.67	2888.01	14094.68	7.96	15.14	3.06	-1.19	-10.67	-25.60	24.41
Shuiquan	19XE-SQ-40	thin-bedded limestone	45.96	73.58	0.002	10060.67	2671.43	3639.05	3.77	7.68	3.10	-1.79	-9.40	-26.74	24.95
Shuiquan	19XE-SQ-39	thin-bedded limestone	45.37	77.12	0.002	9852.31	2529.56	3941.82	3.89	10.43	2.70	-1.71	-11.08	-26.83	25.11
Shuiquan	19XE-SQ-38	thin-bedded limestone	44.70	29.89										-27.23	
Shuiquan	19XE-SQ-37	thin-bedded limestone	44.52	72.45	0.002	8484.86	2305.02	3410.34	3.68	7.34	2.67	-1.20	-10.51	-26.81	25.61
Shuiquan	19XE-SQ-36	thin-bedded limestone	44.30	35.65											
Shuiquan	19XE-SQ-35	thin-bedded limestone	43.50	11.40										-28.23	
Shuiquan	19XE-SQ-34	thin-bedded limestone	43.16	57.58	0.002	13282.11	3132.72	5299.13	4.24	11.23	3.53	-1.84	-11.10	-27.46	25.62
Shuiquan	19XE-SQ-33	thin-bedded limestone	42.90	6.42										-27.17	
Shuiquan	19XE-SQ-32	thin-bedded limestone	42.73	74.15	0.001	6583.19	2826.13	3254.08	2.33	12.38	3.58	-2.26	-10.33	-27.08	24.81
Shuiquan	19XE-SQ-31	thin-bedded limestone	42.48	66.18	0.002	14911.90	2933.96	6461.48	5.08	11.98	3.46	-1.58	-11.24	-28.16	26.58
Shuiquan	19XE-SQ-30	thin-bedded limestone	41.97	47.27	0.005	28968.79	2938.54	16265.15	9.86	14.88	3.16	-0.92	-8.85	-27.66	26.74
Shuiquan	19XE-SQ-29	thin-bedded limestone	41.80	9.84										-27.68	
Shuiquan	19XE-SQ-28	thin-bedded limestone	41.63	60.18	0.002	11197.10	2173.44	5020.54	5.15	11.51	2.30	-1.67	-10.76	-26.12	24.46
Shuiquan	19XE-SQ-27	thin-bedded limestone	41.20												
Shuiquan	19XE-SQ-26	thin-bedded limestone	40.70	14.23										-25.55	
Shuiquan	19XE-SQ-25	marlstone and limestone	40.52	95.18	0.002	1874.84	657.88	356.24	2.85	0.63	0.88	0.86	-10.15	-28.15	29.00
Shuiquan	19XE-SQ-24	marlstone and limestone	40.27		0.002	5805.92	816.84	360.89	7.11	0.93	1.49	-1.76	-10.87		
Shuiquan	19XE-SQ-23	marlstone and limestone	39.33	95.17	0.002	3564.28	719.15	398.36	4.96	0.85	1.11	0.26	-10.01	-27.18	27.44
Shuiquan	19XE-SQ-22	marlstone and limestone	39.16	95.15	0.002	3025.54	859.71	219.72	3.52	0.57	1.28	-0.80	-8.22	-27.33	26.53
Shuiquan	19XE-SQ-21	marlstone and limestone	38.99	89.51	0.002	3938.91	714.44	311.38	5.51	0.75	1.06	-0.64	-7.48	-28.99	28.35
Shuiquan	19XE-SQ-20	marlstone and limestone	38.82		0.002	1922.35	666.33	258.09	2.88	0.51	0.63	-0.38	-10.15		
Shuiquan	19XE-SQ-19	marlstone and limestone	38.65	91.53	0.002	3130.10	2556.31	130.57	1.22	0.25	2.57	-0.27	-10.76	-28.43	28.17
Shuiquan	19XE-SQ-18-2	marlstone and limestone	38.50	68.73										-28.50	

Shuiquan	19XE-SQ-18-1	marlstone and limestone	38.48	73.73	0.001	2863.20	1444.36	669.19	1.98	2.44	1.70	-1.68	-10.58	-29.81	28.13
Shuiquan	19XE-SQ-17	marlstone and limestone	38.40	79.11	0.002	2523.62	893.47	381.57	2.82	0.75	1.15	-0.09	-10.82	-29.01	28.92
Shuiquan	19XE-SQ-16-2	marlstone and limestone	38.23	83.00	0.002	3882.99	760.56	804.81	5.11	1.98	0.91	-0.54	-10.72	-29.01	28.46
Shuiquan	19XE-SQ-16-1	marlstone and limestone	37.50	81.14										-27.46	
Shuiquan	19XE-SQ-15	marlstone and limestone	37.00	11.20										-29.54	
Shuiquan	19XE-SQ-14	marlstone and limestone	30.18	92.09	0.001	883.48	174.28	227.77	5.07	0.79	0.45	-8.04	-11.00	-27.69	19.65
Shuiquan	19XE-SQ-13	marlstone and limestone	29.90	88.43	0.001	1195.74	130.21	320.77	9.18	0.92	0.26			-25.84	
Shuiquan	19XE-SQ-12	marlstone and limestone	28.52	88.66	0.001	1311.42	503.54	235.17	2.60	0.83	1.42	-8.40	-10.49	-27.09	18.70
Shuiquan	19XE-SQ-11	marlstone and limestone	28.24	86.34	0.001	1434.64	320.52	235.57	4.48	0.94	0.72	-8.90	-11.62	-25.07	16.16
Shuiquan	19XE-SQ-10	marlstone and limestone	28.00												
Shuiquan	19XE-SQ-9	limestone-marl alternations	27.69	91.84	0.001	1664.60	343.87	268.59	4.84	1.07	0.74	-8.58	-11.27	-26.71	18.13
Shuiquan	19XE-SQ-8	limestone-marl alternations	27.14	89.50	0.001	1412.76	404.57	386.77	3.49	1.78	1.21	-8.97	-11.19	-26.59	17.62
Shuiquan	19XE-SQ-7	limestone-marl alternations	26.59	88.56	0.001	1686.80	741.77	220.62	2.27	1.07	2.16	-8.90	-10.97		
Shuiquan	19XE-SQ-6-1	limestone-marl alternations	26.04	80.84	0.001	1425.46	651.64	324.12	2.19	1.41	1.68			-23.58	
Shuiquan	19XE-SQ-6-2	limestone-marl alternations	25.80	84.49								-9.56	-11.94	-25.13	15.57
Shuiquan	19XE-SQ-5	limestone-marl alternations	25.48	71.78	0.037	18517.08	2014.97	564.72	9.19	0.07	3.26	-7.48	-7.55		
Shuiquan	19XE-SQ-4	limestone-marl alternations	25.21	94.38	0.001	1280.55	591.16	478.89	2.17	2.45	2.04	-9.51	-11.52		
Shuiquan	19XE-SQ-3	limestone-marl alternations	24.66	77.12	0.046	34657.84	4019.15	847.28	8.62	0.08	4.98	-7.63	-8.32	-27.46	19.83
Shuiquan	19XE-SQ-2	limestone-marl alternations	24.20	83.75	0.001	1831.76	889.20	449.05	2.06	1.35	2.45	-9.75	-11.63	-28.73	18.98
Shuiquan	19XE-SQ-1	limestone-marl alternations	24.00	62.89	0.001	1713.01	992.74	592.33	1.73	1.91	2.91	-9.59	-12.01	-27.02	17.43
Shuiquan	19MK-SQ2-1-1	limestone-marl alternations	23.55	72.25	0.031	17413.41	2195.48	584.65	7.93	0.08	3.87	-6.57	-4.44	-25.03	18.46
Shuiquan	19MK-SQ2-1-2	limestone-marl alternations	23.55	61.94	0.003	3940.31	618.73	1320.67	6.37	1.82	1.17	-9.51	-10.32	-25.27	15.75
Shuiquan	19MK-SQ2-2-1	limestone-marl alternations	23.40	64.06	0.001	2853.12	572.86	902.60	4.98	3.19	1.37	-9.60	-10.44	-25.93	16.33
Shuiquan	19MK-SQ2-2-2	limestone-marl alternations	23.40		0.001	2122.36	526.34	343.35	4.03	1.44	1.50	-10.09	-10.85		
Shuiquan	19MK-SQ2-3	limestone-marl alternations	23.37	66.66	0.001	2556.08	573.72	1157.20	4.46	5.21	1.58	-10.45	-11.02	-26.57	16.12
Shuiquan	19MK-SQ2-4-1	limestone-marl alternations	23.28	76.31	0.002	3281.19	801.73	1389.37	4.09	3.21	1.73	-9.82	-10.45	-27.46	17.65
Shuiquan	19MK-SQ2-4-2	limestone-marl alternations	23.28	79.64	0.001	2599.53	618.72	1434.62	4.20	6.61	1.41	-10.43	-11.33		
Shuiquan	19MK-SQ2-5	limestone-marl alternations	23.18	70.96	0.001	2366.80	534.11	752.53	4.43	3.21	1.13	-10.66	-11.00	-28.01	17.35
Shuiquan	19MK-SQ2-6	limestone-marl alternations	23.00	72.86	0.054	37410.61	3499.67	2566.92	10.69	0.21	7.22	-8.29	-4.64	-29.46	21.17
Shuiquan	19MK-SQ2-7-1	limestone-marl alternations	22.82	39.00	0.044	9620.66	3239.41	1203.21	2.97	0.12	6.78	-7.34	-3.57	-26.06	18.72
Shuiquan	19MK-SQ2-7-2	limestone-marl alternations	22.82	66.32								-8.31	-4.83	-25.73	17.42
Shuiquan	19MK-SQ2-8	limestone-marl alternations	22.63	74.75	0.060	34028.67	4329.09	4251.91	7.86	0.31	10.66	-9.83	-6.94	-27.30	17.47
Shuiquan	19MK-SQ2-9	limestone-marl alternations	22.36	40.05	0.050	37589.35	3794.45	1295.97	9.91	0.11	7.86			-26.30	
Shuiquan	19MK-SQ2-10	limestone-marl alternations	22.20	21.07										-25.74	
Shuiquan	19MK-SQ2-11	limestone-marl alternations	22.00	75.24								-8.22	-5.24	-26.46	18.24
Shuiquan	19MK-SQ2-12	limestone-marl alternations	21.99	79.47	0.054	17052.45	4042.11	232.05	4.22	0.02	8.13	-8.06	-4.79	-25.86	17.80
Shuiquan	19MK-SQ2-13	limestone-marl alternations	21.90	77.23	0.056	13740.94	3971.01	256.80	3.46	0.02	8.34	-9.03	-7.46	-27.91	18.88
Shuiquan	19MK-SQ2-14	limestone-marl alternations	21.80	77.93	0.065	34327.17	5746.83	318.24	5.97	0.02	5.66	-9.11	-7.19	-29.84	20.74

Shuiquan	19MK-SQ2-15	limestone-marl alternations	21.71	69.34	0.071	27615.23	5644.35	309.16	4.89	0.02	6.20	-9.06	-8.16	-31.40	22.34
Shuiquan	19MK-SQ2-16	limestone-marl alternations	21.53	79.75	0.073	30122.48	5255.23	2052.82	5.73	0.13	4.59			-29.24	
Shuiquan	19MK-SQ2-17	limestone-marl alternations	21.34	78.98	0.074	30270.22	5196.99	1801.23	5.82	0.11	4.57	-9.14	-7.56	-32.12	22.98
Shuiquan	19MK-SQ2-18	limestone-marl alternations	21.25	80.40	0.069	36343.36	5849.12	348.46	6.21	0.02	5.73	-9.37	-6.88		
Shuiquan	19MK-SQ2-19	limestone-marl alternations	21.16	78.97								-9.19	-6.91	-30.72	21.53
Shuiquan	19MK-SQ2-20	limestone-marl alternations	21.07	76.47	0.073	27844.40	5134.93	970.59	5.42	0.06	8.28	-9.02	-6.73	-31.93	22.91
Shuiquan	19MK-SQ2-21	limestone-marl alternations	20.88	84.08	0.077	33491.01	5354.93	990.48	6.25	0.06	6.92	-9.30	-7.33	-30.97	21.67
Shuiquan	19MK-SQ2-22	limestone-marl alternations	20.70	85.82										-31.73	
Shuiquan	19MK-SQ2-23	limestone-marl alternations	20.61	79.43	0.074	27948.59	4399.42	1337.45	6.35	0.08	6.34	-9.18	-6.98		
Shuiquan	19MK-SQ2-24	limestone-marl alternations	20.42	77.92	0.075	30094.44	5312.06	961.61	5.67	0.06	6.87	-8.96	-6.92	-31.37	22.40
Shuiquan	19MK-SQ2-25	limestone-marl alternations	20.24	92.84	0.083	7108.19	957.05	247.78	7.43	0.01	4.01	-6.06	-4.95	-26.74	20.68
Shuiquan	19MK-SQ2-26	limestone-marl alternations	19.32	92.21	0.082	17726.05	3669.59	192.11	4.83	0.01	10.44	-10.69	-6.37	-25.82	15.13
Shuiquan	19MK-SQ-72	limestone-marl alternations	17.20	89.25	0.001	1339.33	249.76	715.76	5.36	2.31	0.79	-8.16	-10.50	-24.82	16.66
Shuiquan	19MK-SQ-71	limestone-marl alternations	17.10	90.78										-25.63	
Shuiquan	19MK-SQ-70	limestone-marl alternations	16.93	91.98	0.001	1060.23	128.04	579.53	8.28	1.67	0.36	-7.94	-10.35		
Shuiquan	19MK-SQ-69	limestone-marl alternations	16.84	84.65	0.001	976.91	136.72	710.08	7.15	2.86	0.46	-8.41	-11.25	-25.85	17.44
Shuiquan	19MK-SQ-68	limestone-marl alternations	16.65	87.34	0.001	1178.51	120.55	683.83	9.78	2.26	0.37	-8.55	-10.87	-25.61	17.05
Shuiquan	19MK-SQ-67	limestone-marl alternations	16.10	81.54	0.036	9122.09	594.84	575.43	15.34	0.07	1.22	-6.73	-6.62	-24.94	18.22
Shuiquan	19MK-SQ-66	limestone-marl alternations	15.92	80.05	0.001	1019.14	123.19	655.16	8.27	2.51	0.27	-8.45	-11.14	-23.87	15.42
Shuiquan	19MK-SQ-65	limestone-marl alternations	15.64	84.46	0.001	1050.78	195.91	679.00	5.36	2.41	0.52	-8.77	-11.18	-25.06	16.29
Shuiquan	19MK-SQ-64	limestone-marl alternations	15.18	86.41	0.001	1079.30	208.90	396.91	5.17	1.29	0.59	-8.79	-11.25	-28.03	19.24
Shuiquan	19MK-SQ-63	limestone-marl alternations	15.00	82.62	0.001	1074.72	217.10	383.96	4.95	1.56	0.68	-8.93	-11.30	-26.23	17.30
Shuiquan	19MK-SQ-62	limestone-marl alternations	14.54	86.56	0.001	1223.13	191.43	484.51	6.39	1.64	0.60	-8.83	-10.91	-24.42	15.59
Shuiquan	19MK-SQ-61	limestone-marl alternations	14.26	87.60	0.001	1486.95	215.15	666.26	6.91	3.35	0.68	-8.87	-10.71	-21.46	12.59
Shuiquan	19MK-SQ-61A	limestone-marl alternations	13.80	73.58	0.001	1517.08	194.07	661.51	7.82	3.37	0.62	-8.86	-10.99	-24.06	15.19
Shuiquan	19MK-SQ-60	limestone-marl alternations	13.71	84.66	0.001	1453.37	185.19	503.24	7.85	2.44	0.44	-8.63	-10.62	-25.11	16.48
Shuiquan	19MK-SQ-59	limestone-marl alternations	13.43	83.36	0.001	1455.20	195.45	431.76	7.45	1.92	0.46	-8.77	-11.06	-27.17	18.40
Shuiquan	19MK-SQ-58	limestone-marl alternations	13.16	88.31	0.001	1411.32	218.78	337.14	6.45	1.39	0.85	-9.22	-11.47	-26.81	17.59
Shuiquan	19MK-SQ-57	limestone-marl alternations	13.00	86.02										-27.08	
Shuiquan	19MK-SQ-56	limestone-marl alternations	12.80	81.17										-25.30	
Shuiquan	19MK-SQ-55	limestone-marl alternations	12.60	88.42	0.001	1651.09	238.17	454.42	6.93	2.06	0.80	-9.01	-10.92		
Shuiquan	19MK-SQ-54	limestone-marl alternations	12.42	82.10	0.001	1730.81	322.39	366.96	5.37	1.51	1.31	-9.27	-11.13		
Shuiquan	19MK-SQ-53	limestone-marl alternations	12.33	87.53	0.001	1550.11	309.08	387.53	5.02	1.62	1.16	-9.48	-11.69	-26.58	17.10
Shuiquan	19MK-SQ-52	limestone-marl alternations	11.96	59.91	0.002	3426.07	312.99	1383.22	10.95	3.01	0.70	-8.49	-11.45		
Shuiquan	19MK-SQ-51-2	limestone-marl alternations	11.68									-9.58	-11.36		
Shuiquan	19MK-SQ-51-1	limestone-marl alternations	11.68	82.24	0.004	9092.55	505.14	523.29	18.00	0.58	1.16	-9.41	-11.61		
Shuiquan	19MK-SQ-50	limestone-marl alternations	11.59	71.04	0.001	1859.45	309.91	409.37	6.00	1.57	1.05	-9.70	-11.51	-23.69	13.99
Shuiquan	19MK-SQ-49	microcrystalline limestone	11.41	75.60	0.001	1952.00	342.68	534.65	5.70	1.97	1.07	-9.49	-11.31	-25.97	16.48

Shuiquan	19MK-SQ-48	microcrystalline limestone	11.32	80.77	0.001	1406.41	343.07	301.37	4.10	1.43	1.51	-9.57	-11.03	-25.13	15.56
Shuiquan	19MK-SQ-47	microcrystalline limestone	11.22	76.80	0.001	1509.44	377.36	278.06	4.00	1.26	1.50	-9.77	-11.42	-25.08	15.30
Shuiquan	19MK-SQ-46	microcrystalline limestone	11.13	69.13	0.001	1924.96	339.28	671.50	5.67	2.96	1.02	-9.38	-11.25	-23.05	13.68
Shuiquan	19MK-SQ-45	microcrystalline limestone	11.04	61.68	0.001	1996.73	288.97	595.33	6.91	2.20	0.77	-9.30	-11.04	-20.68	11.38
Shuiquan	19MK-SQ-44	microcrystalline limestone	10.95	81.19	0.001	1872.43	325.06	935.26	5.76	3.81	0.98	-9.77	-11.67	-26.05	16.27
Shuiquan	19MK-SQ-43	microcrystalline limestone	10.86	76.78	0.001	2020.47	341.26	1170.03	5.92	4.16	1.08	-9.90	-11.46	-25.76	15.86
Shuiquan	19MK-SQ-42	microcrystalline limestone	10.76	85.10	0.001	2096.62	395.48	955.90	5.30	4.16	1.69	-9.78	-10.99	-26.08	16.30
Shuiquan	19MK-SQ-41	microcrystalline limestone	10.67	69.26	0.002	7067.84	382.33	1416.49	18.49	3.32	1.06	-9.96	-11.62	-24.25	14.29
Shuiquan	19MK-SQ-40	microcrystalline limestone	10.40	74.92	0.002	2838.42	459.65	1335.73	6.18	3.12	1.31	-9.51	-10.97	-25.93	16.41
Shuiquan	19MK-SQ-39	microcrystalline limestone	10.25	83.48										-25.03	
Shuiquan	19MK-SQ-38	microcrystalline limestone	10.21	68.61	0.004	8384.34	816.98	1127.34	10.26	1.20	2.05	-9.07	-10.19	-24.48	15.41
Shuiquan	19MK-SQ-37	microcrystalline limestone	10.03	71.74	0.001	2719.40	639.86	801.27	4.25	2.75	1.80	-9.73	-11.55	-25.74	16.01
Shuiquan	19MK-SQ-36	microcrystalline limestone	9.94	62.31	0.001	1456.96	435.75	712.17	3.34	3.23	1.72	-9.84	-11.37	-25.05	15.20
Shuiquan	19MK-SQ-35	microcrystalline limestone	9.84	90.82	0.001	1207.07	241.41	1096.34	5.00	3.10	0.73	-7.84	-9.06	-26.07	18.23
Shuiquan	19MK-SQ-34	microcrystalline limestone	9.57	92.67	0.001	1002.38	397.25	771.85	2.52	2.41	1.21	-7.57	-7.99	-24.84	17.26
Shuiquan	19MK-SQ-33	microcrystalline limestone	9.29	96.53	0.001	1215.53	378.45	744.07	3.21	2.13	1.05	-7.56	-9.31		
Shuiquan	10MK-SQ-32	microcrystalline limestone	9.00	92.88										-26.78	
Shuiquan	19MK-SQ-31	microcrystalline limestone	8.74	92.40	0.001	1207.51	137.10	921.01	8.81	2.70	0.35	-7.93	-10.12	-26.52	18.59
Shuiquan	19MK-SQ-30	microcrystalline limestone	8.60	91.53										-25.70	
Shuiquan	19MK-SQ-29	microcrystalline limestone	8.46	92.03	0.001	1201.14	298.89	561.46	4.02	1.72	0.92	-8.10	-9.94		
Shuiquan	19MK-SQ-28	microcrystalline limestone	8.20	91.04										-26.88	
Shuiquan	19MK-SQ-27	microcrystalline limestone	8.10	91.15	0.001	1239.27	181.42	675.46	6.83	2.33	0.51	-8.22	-10.22	-25.73	17.52
Shuiquan	19MK-SQ-26	microcrystalline limestone	7.82	92.95	0.002	971.63	113.30	532.16	8.58	1.46	0.31	-8.35	-10.11	-26.45	18.11
Shuiquan	19MK-SQ-25	microcrystalline limestone	7.54	92.79	0.001	1021.68	185.29	666.69	5.51	2.05	0.56	-8.40	-10.52	-26.49	18.08
Shuiquan	19MK-SQ-24	microcrystalline limestone	7.27	92.38	0.001	1895.57	197.16	435.38	9.61	1.89	0.41	-8.53	-10.92	-25.99	17.47
Shuiquan	19MK-SQ-23	microcrystalline limestone	6.99	91.64	0.001	1112.97	215.13	899.62	5.17	2.77	0.73	-8.63	-9.98	-24.72	16.09
Shuiquan	19MK-SQ-22	microcrystalline limestone	6.81	92.58	0.001	1126.64	207.05	1012.86	5.44	3.04	0.59	-8.58	-10.51	-27.17	18.60
Shuiquan	19MK-SQ-21	microcrystalline limestone	6.26	92.72	0.001	1155.49	210.88	993.41	5.48	2.93	0.73	-8.73	-9.28	-26.78	18.05
Shuiquan	19MK-SQ-20	microcrystalline limestone	5.98	92.28	0.001	1225.59	204.26	897.69	6.00	2.93	0.62	-8.87	-10.10	-25.73	16.86
Shuiquan	19MK-SQ-19	microcrystalline limestone	5.34	92.64	0.001	1266.62	211.96	741.85	5.98	2.51	0.63	-8.26	-10.67	-26.97	18.72
Shuiquan	19MK-SQ-18	microcrystalline limestone	5.06	93.09	0.001	1185.51	222.95	636.70	5.32	2.28	0.77	-8.41	-10.34	-25.29	16.88
Shuiquan	19MK-SQ-17	microcrystalline limestone	4.97	93.28	0.001	1519.38	247.96	722.01	6.13	2.69	0.59	-8.26	-10.44	-27.75	19.48
Shuiquan	19MK-SQ-16	microcrystalline limestone	4.78	90.68	0.001	1326.26	313.96	625.90	4.22	2.00	0.76	-8.75	-11.06	-28.06	19.31
Shuiquan	19MK-SQ-15	limestone	4.69	91.43	0.077	21375.25	3422.17	304.42	6.25	0.02	2.83	-8.78	-7.95	-31.19	22.42
Shuiquan	19MK-SQ-14	limestone	4.60	82.75	0.082	12191.67	3172.75	186.08	3.84	0.01	6.13	-5.31	-6.03	-30.78	25.47
Shuiquan	19MK-SQ-13	limestone	4.51	54.02	0.079	30411.90	7441.18	507.94	4.09	0.03	7.29	-7.36	-8.45		
Shuiquan	19MK-SQ-12	limestone	4.42	87.22	0.084	36968.58	10291.88	360.93	3.59	0.02	9.93	-9.13	-8.39	-28.19	19.06
Shuiquan	19MK-SQ-11	limestone	3.50	36.30	0.068	44001.35	12396.36	19851.40	3.55	1.28	42.35	-1.08	-1.64		

Shuiquan	19MK-SQ-10	limestone	2.58	70.20	0.075	41126.60	16293.08	10241.51	2.52	0.60	41.63	-1.39	-2.02	-27.48	26.09
Shuiquan	19MK-SQ-9	limestone	2.39	83.00	0.079	51970.41	18354.04	6567.90	2.83	0.37	33.41	-1.76	-2.75	-23.31	21.55
Shuiquan	19MK-SQ-8	shale	2.30	72.66										-25.29	
Shuiquan	19MK-SQ-7	limestone	2.21	48.89	0.077	34346.26	16004.59	8760.90	2.15	0.51	35.73	-0.65	-0.96		
Shuiquan	19MK-SQ-6	limestone	1.84	62.47	0.060	12363.95	19329.70	5746.00	0.64	0.42	44.84	-2.92	-0.67	-24.42	21.50
Shuiquan	19MK-SQ-5	limestone	1.38	40.97	0.076	67851.51	22006.04	7102.36	3.08	0.42	38.98	-2.14	-3.22		
Shuiquan	19MK-SQ-5A	limestone	1.29	74.00	0.069	70155.56	29993.84	4019.62	2.34	0.26	47.85	-4.91	-5.82	-24.55	19.64
Shuiquan	19MK-SQ-4	shale	0.80												
Shuiquan	19MK-SQ-3	limestone	0.64	72.09	0.072	64297.94	36453.19	4979.15	1.76	0.31	36.82	-4.62	-4.68	-28.33	23.71
Shuiquan	19MK-SQ-2	shale	0.30	9.54										-27.71	
Shuiquan	19MK-SQ-1	limestone	0.00	67.36	0.071	25761.20	56680.31	4692.32	0.45	0.29	49.47	-5.50	-3.26	-26.26	20.76

Table S3. Carbonate Carbon ($\delta^{13}C_{carb}$), Oxygen Isotopes($\delta^{18}O$) and Organic Carbon Isotopes ($\delta^{13}C_{org}$) of Shuiquan Formation, HZ section, South China.

Formation	Samples	Lithology	Height (m)	$\delta^{13}C_{carb}$ (‰)	$\delta^{18}O_{carb}$ (‰)
Shuiquan	AFQ536	shale-calcareous siltstone	434.5		
Shuiquan	AFQ537	shale-calcareous siltstone	436		
Shuiquan	AFQ538	shale-calcareous siltstone	438		
Shuiquan	AFQ539	shale-calcareous siltstone	439		
Shuiquan	AFQ540	shale	442		
Shuiquan	AFQ541	thin-bedded limestone	443		
Shuiquan	AFQ542	shale	448		
Shuiquan	AFQ543	shale-calcareous siltstone	452		
Shuiquan	AFQ544	thin-bedded limestone	458	-1.6	-3.6
Shuiquan	AFQ545	thin-bedded limestone	461	-2.2	-4.4
Shuiquan	AFQ546	thin-bedded limestone	471		
Shuiquan	AFQ547	thin-bedded limestone	476		
Shuiquan	AFQ548	thin-bedded limestone	481	-1.3	-10.7
Shuiquan	AFQ549	thin-bedded limestone	483	-1.2	-10.7
Shuiquan	AFQ550	thin-bedded limestone	487	-0.3	-2.7
Shuiquan	AFQ551	thin-bedded limestone	490		
Shuiquan	AFQ552	thick-bedded limestone	493	-3.9	-12.1
Shuiquan	AFQ553	thick-bedded limestone	498	-1.9	-3.1
Shuiquan	AFQ554	thick-bedded limestone	501	-1.5	-10.0
Shuiquan	AFQ555	thick-bedded limestone	504	-4.4	-4.5
Shuiquan	AFQ556	thick-bedded limestone	507	-5.7	-4.6
Shuiquan	AFQ557	light-gray limestone	522	-7.3	-12.8
Shuiquan	AFQ558	light-gray limestone	524	-7.4	-11.2
Shuiquan	AFQ559	light-gray limestone	526	-7.8	-13.0
Shuiquan	AFQ560	light-gray limestone	528	-8.0	-13.2
Shuiquan	AFQ561	light-gray limestone	530	-8.4	-13.0
Shuiquan	AFQ562	limestone-marl alternations	532	-8.7	-13.2
Shuiquan	AFQ563-a	brown limestone		-5.6	-6.4
Shuiquan	AFQ563-b	limestone-marl alternations	534	-9.1	-13.5
Shuiquan	AFQ564	limestone-marl alternations	536	-9.0	-13.5
Shuiquan	AFQ565	limestone-marl alternations	538	-9.9	-13.7
Shuiquan	AFQ566	limestone-marl alternations	540	-10.2	-13.6
Shuiquan	AFQ567	limestone-marl alternations	542	-10.7	-13.0
Shuiquan	AFQ568	limestone-marl alternations	542.2	-11.1	-13.7
Shuiquan	AFQ569	reddish-brown dolomite	542.6	-11.2	-8.0
Shuiquan	AFQ570	light-gray limestone	543.6	-10.1	-13.0
Shuiquan	AFQ571	reddish-brown dolomite	544.6	-10.9	-8.2
Shuiquan	AFQ572	reddish-brown dolomite	545.6	-11.1	-8.4
Shuiquan	AFQ573	reddish-brown dolomite	546.6	-8.7	-7.6
Shuiquan	AFQ574	reddish-brown dolomite	547	-8.7	-6.6

Table S4. Paleomagnetic poles for the absolute reconstruction in Figures 5 and S7

Pole ID	Rock Unit (Area)	Age	Age range (Ma)		P-lat	P-long	A_{95}	1	2	3	4	5	6	7	Rf	Reference
		(Ma)	Max	Min	(°N)	(°E)	(°)									
Laurentia (L)																
L-Sla	Sept-Iies intrusion (Shallow)	565 ± 4	569	561	-20	321	6.7	1	1	1	1	1	0	0	5	Tanczyk et al., 1987
L-Cat	Catoctin Basalts (steep)	572 ± 5	577	567	42	297	17.0	1	1	1	1	1	1	1	7	Meert et al., 1994
L-Cc	Callander Alkaline Complex intrusive	577 ± 1	578	576	46	301	6.0	1	1	1	1	0	1	1	6	Symons and Chiasson, 1991
L-Bm	Baie des Moutons complex	583 ± 2	585	581	42.6	332.7	12.0	1	1	1	0	0	0	1	4	McCausland et al., 2011
L-GDb	Grenville Dikes (Steep)	587.3 ± 0.7	588	587	55.7	233.4	10.6	1	1	1	1	1	0	1	6	Halls et al., 2015
L-GDe	Grenville Dikes (Steep)	586 ± 4	590	581	51.5	231.2	11.6	1	1	1	1	1	0	1	6	Hyodo and Dunlop, 1993
L-GD2	Grenville Dikes (Steep)	590 ± 2	592	588	61.6	249.9	14.5	1	0	1	1	1	1	1	6	Murthy, 1971
Avalonia (Av)																
Av-CH	Crown Hill Fm, Newfoundland, Canada	< 557+/-14 Ma; 566+/-13	570	540	-48.1	9.9	3.3	1	1	1	1	1	1	1	7	Wen et al., 2020
Av-BAb	Bonavista Bull Arm Fm, Newfoundland, Canada	592 ± 2.2; 591.3 ± 1.6	594	589	-15.5	278.2	11.9	1	1	1	1	1	1	1	7	Wen et al., 2020
Av-BAa	Argentia Bull Arm Formation, Newfoundland, Canada	< 600			-2.9	256.3	10.6	0	1	1	1	1	1	1	6	Pisarevsky et al., 2012; reference age of Mills et al., 2017
Av-MGv	Marystown Group volcanic-sedimentary sequence, Newfoundland, Canada	585 ± 2; 580 ± 3; 576.8 ± 2.6; 575 ± 2	587	573	26.6	239.6	11.1	1	1	1	1	1	1	1	7	McNamara et al., 2001; Sparkes and Dunning, 2014
Av-LMv	Lynn-Mattapan Volcanics, New England, USA	595.8 ± 1.2; 597.4 ± 1.5; 596.0 ± 1.4; 595.7 ± 1.6	599	594	60	206	8.4	1	1	1	1	1	1	1	7	Thompson et al., 2007
Av-Cv	Caldecote Volcanics, England (East Avalonia)	603 ± 2	605	601	-5	329.7	8.2	1	1	1	1	1	0	1	6	Vizan et al., 2003
West Africa (WA)																
WA-Ft	Fajjoud and Tadoughast Volcanics, Morocco	566 ± 6; 564 ± 6; 567 ± 5; 565 ± 5; 556± 5	572	551	21.9	31	15.6	1	1	1	1	0	1	1	6	Robert et al., 2017, 2018
WA-At	Adrar-n-takoucht Volcanics	572 ± 5; 570 ± 6	577	564	-57.6	295.6	15.7	1	0	1	1	0	1	1	5	Robert et al., 2017, 2018
Australia (Au)																

Au-Wk	Wonoka Formation, S. Australia	~ 565	Fossils		-5.2	30.5	4.9	0	1	0	1	1	1	1	5	Schmidt and Williams, 2010
Au-Arl	Lower Arumbera sandstone	~ 570	Strati		-53.9	348.1	8.8	0	0	1	1	1	1	1	5	Mitchell et al., 2010
Baltica (B)																
B-Bf	Basu Formation	570-560	Fossils		-2	6	3.8	1	1	1	1	1	1	0	6	Levashova et al., 2015
B-Kg	Kurgashlya formation	600-570?			50.9	314.5	5.3	0	1	1	1	1	1	1	6	Lubnina et al., 2014
B-Bk	Bakeevo formation	600-570?			42.3	299.1	5.3	0	1	1	1	1	1	1	6	Lubnina et al., 2014
**Tarim (T)**																
T-Zma	Zhamoketi Formation	< 600			4.9	326.7	3.9	0	1	1	0	0	0	1	3	Zhao et al., 2014
**Siberia (Si)**																
Si-Mf	Minya Formation	590-570			33.7	217.2	12.7	0	0	1	0	1	1	0	3	Kravchinsky et al., 2001
Si-Sf	Shaman Formation	590-570	Fossils		32	251.1	13.8	0	1	1	1	1	1	0	5	Kravchinsky et al., 2001
South China (Sc)																
Sc-DSTm3	Doushantuo Formation Member3	591.1 ± 5.3	596	586	-31.3	349.8	4.1	0	1	1	1	1	1	1	6	Jing et al., 2018; age from Zhu et al., 2013
North China (Nc)																
Nc-Hf	Hsuchuang Formation	505	Fossils		31.8	320.4	5.3	1	1	1	1	1	1	0	6	Zhao et al., 2020
Nc-XW	Xinjin and Wudaotang formations	514-509	Fossils		18.5	341.9	6.5	1	1	1	1	1	0	1	6	Huang et al., 1999

Notes: P-lat/P-long, latitude/longitude of a paleomagnetic pole. $A_{95,}$radius of the pole 95% confidence cone. R_f, Reliability factor of Meert et al., 2020.

Reference：

Halls, H.C., Lovette, A., Hamilton, M., Söderlund, U., 2015. A paleomagnetic and U–Pb geochronology study of the western end of the Grenville dyke swarm: Rapid changes in paleomagnetic field direction at ca. 585Ma related to polarity reversals? Precambrian Research 257, 137-166.

Huang, B., Yang, Z., Otofuji, Y.-i., Zhu, R., 1999. Early Paleozoic paleomagnetic poles from the western part of the North China Block and their implications. Tectonophysics 308, 377-402.

Hyodo, H., Dunlop, D.J., 1993. Effect of anisotropy on the paleomagnetic contact test for a Grenville Dike. Journal of Geophysical Research: Solid Earth 98, 7997-8017.

Jing, X., Yang, Z., Tong, Y., Wang, H., Xu, Y., 2018. Identification of multiple magnetizations of the Ediacaran strata in South China. Geophysical Journal International 212, 54-75.

Kravchinsky, V.A., Konstantinov, K.M., Cogné, J.P., 2001. Palaeomagnetic study of Vendian and Early Cambrian rocks of South Siberia and Central Mongolia: was the Siberian platform assembled at this time? Precambrian Research 110, 61-92.

Levashova, N.M., Bazhenov, M.L., Meert, J.G., Danukalov, K.N., Golovanova, I.V., Kuznetsov, N.B., Fedorova, N.M., 2015. Paleomagnetism of upper Ediacaran clastics from the South Urals: Implications to paleogeography of Baltica and the opening of the Iapetus Ocean. Gondwana Research 28, 191-208.

Lubnina, N.V., Pisarevsky, S.A., Puchkov, V.N., Kozlov, V.I., Sergeeva, N.D., 2014. New paleomagnetic data from Late Neoproterozoic sedimentary successions in Southern Urals, Russia: implications for the Late Neoproterozoic paleogeography of the Iapetan realm. International Journal of Earth Sciences 103, 1317-1334.

McCausland, P.J.A., Hankard, F., Van der Voo, R., Hall, C.M., 2011. Ediacaran paleogeography of Laurentia: Paleomagnetism and 40Ar–39Ar geochronology of the 583Ma Baie des Moutons syenite, Quebec. Precambrian Research 187, 58-78.

McNamara, A.K., Niocaill, C.M., van der Pluijm, B.A., der Voo, R.V., 2001. West African proximity of the Avalon terrane in the latest Precambrian. Geological Society of America Bulletin 113, 1161.

Meert, J.G., Van der Voo, R., Payne, T.W., 1994. Paleomagnetism of the Catoctin volcanic province: A new Vendian-Cambrian apparent polar wander path for North America. Journal of Geophysical Research: Solid Earth 99, 4625-4641.

Meert JG, Pivarunas AF, Evans DAD, Pisarevsky SA, Pesonen LJ et al. (2020). The magnificent seven: a proposal for modest revision of the quality index. Tectonophysics 790: 228549.

Mills, A.J., Dunning, G.R., Murphy, M., Langille, A., 2017. New geochronological constraints on the timing of magmatism for the Bull Arm Formation, Musgravetown Group, Avalon Terrane, northeastern Newfoundland. Current Research (2017) Newfoundland and Labrador Department of Natural Resources Geological Survey, Report 17-1, 1-17.

Mitchell, R.N., Evans, D.A.D., Kilian, T.M., 2010. Rapid Early Cambrian rotation of Gondwana. Geology 38, 755-758.

Murthy, G.S., 1971. The Paleomagnetism of Diabase Dikes from the Grenville Province. Canadian Journal of Earth Sciences 8, 802.

Pisarevsky, S.A., McCausland, P.J.A., Hodych, J.P., O’Brien, S.J., Tait, J.A., Murphy, J.B., Colpron, M., 2012. Paleomagnetic study of the late Neoproterozoic Bull Arm and Crown Hill formations (Musgravetown Group) of eastern Newfoundland: implications for Avalonia and West Gondwana paleogeography1This article is one of a series of papers published in CJES Special Issue: In honour of Ward Neale on the theme of Appalachian and Grenvillian geology. Canadian Journal of Earth Sciences 49, 308-327.

Robert, B., Besse, J., Blein, O., Greff-Lefftz, M., Baudin, T., Lopes, F., Meslouh, S., Belbadaoui, M., 2017. Constraints on the Ediacaran inertial interchange true polar wander hypothesis: A new paleomagnetic study in Morocco (West African Craton). Precambrian Research 295, 90-116.

Robert, B., Greff-Lefftz, M., Besse, J., 2018. True Polar Wander: A Key Indicator for Plate Configuration and Mantle Convection During the Late Neoproterozoic. Geochemistry, Geophysics, Geosystems 19, 3478-3495.

Schmidt, P.W., Williams, G.E., 2010. Ediacaran palaeomagnetism and apparent polar wander path for Australia: no large true polar wander. Geophysical Journal International 182, 711-726.

Sparkes, G., Dunning, G., 2014. Late Neoproterozoic epithermal alteration and mineralization in the western Avalon zone: a summary of mineralogical investigations and new u/pb geochronological results. Current Research (2014) Newfoundland and Labrador Department of Natural Resources, Geological Survey, Report 14-1, 99-128.

Symons, D.T.A., Chiasson, A.D., 1991. Paleomagnetism of the Callander Complex and the Cambrian apparent polar wander path for North America. Canadian Journal of Earth Sciences 28, 355.

Tanczyk, E.I., Lapointe, P., Morris, W.A., Schmidt, P.W., 1987. A paleomagnetic study of the layered mafic intrusion at sept-hes, Quebec1. Can. J. Earth Sci. 24, 1431-1438.

Thompson, M.D., Grunow, A.M., Ramezani, J., 2007. Late Neoproterozoic paleogeography of the Southeastern New England Avalon Zone: Insights from U-Pb geochronology and paleomagnetism. Geological Society of America Bulletin 119, 681-696.

Vizan H, Carney JN, Turner P, Ixer RA, Tomasso M et al. (2003). Late Neoproterozoic to Early Palaeozoic palaeogeography of Avalonia: some palaeomagnetic constraints from Nuneaton, central England. Geological Magazine 140 (6): 685-705.

Wen, B., Evans, D.A.D., Anderson, R.P., McCausland, P.J.A., 2020. Late Ediacaran paleogeography of Avalonia and the Cambrian assembly of West Gondwana. Earth and Planetary Science Letters 552, 116591.

Zhao, H., Zhang, S., Zhu, M., Ding, J., Li, H., Yang, T., Wu, H., 2020. Paleomagnetic insights into the Cambrian biogeographic conundrum: Did the North China craton link Laurentia and East Gondwana? Geology 49, 372-376.

Zhao, P., Chen, Y., Zhan, S., Xu, B., Faure, M., 2014. The Apparent Polar Wander Path of the Tarim block (NW China) since the Neoproterozoic and its implications for a long-term Tarim–Australia connection. Precambrian Research 242, 39-57.

Zhu, B., Becker, H., Jiang, S.-Y., Pi, D.-H., Fischer-Gödde, M., Yang, J.-H., 2013. Re–Os geochronology of black shales from the Neoproterozoic Doushantuo Formation, Yangtze platform, South China. Precambrian Research 225, 67-76.

Table S5. Rotation parameters for the absolute reconstruction in this study

Plate ID	Age	Euler Pole		Angle of rotation	Relative to
	Ma	(°N)	(°E)	(°)	
West Africa(714)	560	-5.8	111.8	105.7	Absolute reference(000)
West Africa(714)	590	-20.8	-169.7	-16.9	Absolute reference(000)
Northeast Africa(715)	590-560	-26.3	-164.4	0.5	West Africa(714)
Sao Francisco-Congo(717)	590-560	-68.4	-135.8	0.3	West Africa(714)
Kalahari(716)	590-560	-52.9	-76.5	-5.0	West Africa(714)
Rio Plata(294)	590-560	-73.9	134.2	-62.0	West Africa(714)
India(501)	590-560	41.4	24.2	302.6	West Africa(714)
South China(602)	590-560	23.4	92.7	78.2	India(501)
Baltica(302)	590-560	9.1	9.3	99.3	West Africa(714)
Avalonia(108)	590-560	-5.8	158.8	-84.1	West Africa(714)
Amazonia(201)	590-560	-12.9	36.7	327.1	Avalonia(108)
Laurentia(199)	590-560	-3.8	169.3	-137.9	West Africa(714)
Australia(804)	590-560	-21.6	-54.6	43.3	West Africa(714)
Siberia(400)	590-560	54.6	162.3	-302.6	Laurentia(199)
North China(601)	590-560	-17.9	-51.0	172.9	Siberia(400)
Tarim(605) option 1	590-560	12.1	95.7	197.3	Australia(804)
Tarim(605) option 2	590-560	14.9	109.8	332.5	North China(601)

SI References:

1. Xu B, Jian P, Zheng H *et al.* U-Pb zircon geochronology and geochemistry of Neoproterozoic volcanic rocks in the Tarim Block of northwest China: implications for the breakup of Rodinia supercontinent and Neoproterozoic glaciations. *Precambrian Research*. 2005; **136**(2): 107-123.
2. Shen B, Xiao S, Bao H *et al.* Carbon, sulfur, and oxygen isotope evidence for a strong depth gradient and oceanic oxidation after the Ediacaran Hankalchough glaciation. *Geochimica et Cosmochimica Acta*. 2011; **75**(5): 1357-1373.
3. Xu B, Xiao S, Zou H *et al.* SHRIMP zircon U–Pb age constraints on Neoproterozoic Quruqtagh diamictites in NW China. *Precambrian Research*. 2009; **168**(3-4): 247-258.
4. Xiao S. Neoproterozoic glaciations and biological evolution: Data from the Tarim and Yangtze blocks, China. In: Reimold WU, Hofmann A (eds.). *Abstract Volume of GSA/GSSA Field Forum "Processes on the Early Earth"* 2004. 99.
5. Gao Z, Zhu S. *Precambrian geology in Xinjiang, China*. Urumuqi, China: Xinjiang People's Publishing House, 1984.
6. Li H, Dong Y. Sedimentary features of the Sinian Zhamoketi Formation in the Middle Quruqtagh area of Xinjiang. *Xinjiang Geology*. 1991; **9**(4): 340-351.
7. Kaufman AJ, Knoll AH. Neoproterozoic variations in the C-isotope composition of sea water: Stratigraphic and biogeochemical implications. *Precambrian Research*. 1995; **73**(3-4): 27-49.
8. Jacobsen SB, Kaufman AJ. The Sr, C and O isotopic evolution of Neoproterozoic seawater. *Chemical Geology*. 1999; **161**: 37-57.
9. Knauth LP, Kennedy MJ. The late Precambrian greening of the Earth. *Nature*. 2009; **460**(7256): 728-732.
10. Cui H, Kaufman AJ, Xiao S *et al.* Methane-derived authigenic carbonates from the uppermost Doushantuo Formation in South China: Was the Ediacaran Shuram Excursion a globally synchronized early diagenetic event? *Chemical Geology*. 2017; **450**: 59-80.
11. Derry LA. A burial diagenesis origin for the Ediacaran Shuram-Wonoka carbon isotope anomaly. *Earth and Planetary Science Letters*. 2010; **294**(1-2): 152-162.
12. McFadden KA, Huang J, Chu X *et al.* Pulsed oxidation and biological evolution in the Ediacaran Doushantuo Formation. *Proceedings of the National Academy of Sciences*. 2008; **105**: 3197-3202.
13. Shields GA, Mills BJW, Zhu M *et al.* Unique Neoproterozoic carbon isotope excursions sustained by coupled evaporite dissolution and pyrite burial. *Nature Geoscience*. 2019; **12**(10): 823-827. doi: 10.1038/s41561-019-0434-3
14. Fike DA, Grotzinger JP, Pratt LM *et al.* Oxidation of the Ediacaran ocean. *Nature*. 2006; **444**(7120): 744-747.
15. Grotzinger JP, Fike DA, Fischer WW. Enigmatic origin of the largest-known carbon isotope excursion in Earth's history. *Nature Geoscience*. 2011; **4**(5): 285-292. doi: 10.1038/ngeo1138
16. Husson JM, Higgins JA, Maloof AC *et al.* Ca and Mg isotope constraints on the origin of Earth’s deepest C excursion. *Geochimica et Cosmochimica Acta*. 2015; **160**(0): 243-266. doi: http://dx.doi.org/10.1016/j.gca.2015.03.012
17. Gong Z, Li M. Astrochronology of the Ediacaran Shuram carbon isotope excursion, Oman.

Earth and Planetary Science Letters. 2020; **547**: 116462.

18. Melezhik VA, Pokrovsky BG, Fallick AE *et al.* Constraints on 87Sr/86Sr of Late Ediacaran seawater: insight from Siberian high-Sr limestones. *Journal of the Geological Society*. 2009; **166**(1): 183-191. doi: 10.1144/0016-76492007-171
19. Pokrovskii BG, Melezhik VA, Bujakaite MI. Carbon, oxygen, strontium, and sulfur isotopic compositions in late Precambrian rocks of the Patom Complex, central Siberia: Communication 1. results, isotope stratigraphy, and dating problems. *Lithol Miner Resour*. 2006; **41**(5): 450-474.
20. Chen B, Hu C, Mills BJW *et al.* A short-lived oxidation event during the early Ediacaran and delayed oxygenation of the Proterozoic ocean. *Earth and Planetary Science Letters*. 2022; **577**.
21. Burns SJ, Matter A. Carbon isotope record of the latest Proterozoic from Oman. *Eclogae Geologicae Helvetiae*. 1993; **86**: 595-607.
22. Guerroue E, Allen PA, Cozzi A *et al.* 50-Myr recovery from the largest negative $\delta^{13}C$ excursion in the Ediacaran ocean. *Terra Nova*. 2006; **18**(2): 147-153.
23. Calver CR. Isotope stratigraphy of the Ediacarian (Neoproterozoic III) of the Adelaide Rift Complex, Australia, and the overprint of water column stratification. *Precambrian Research*. 2000; **100**(1): 121-150. doi: https://doi.org/10.1016/S0301-9268(99)00072-8
24. Foden J, Barovich K, Jane M *et al.* Sr-isotopic evidence for Late Neoproterozoic rifting in the Adelaide Geosyncline at 586 Ma: implications for a Cu ore forming fluid flux. *Precambrian Research*. 2001; **106**(3): 291-308. doi: https://doi.org/10.1016/S0301-9268(00)00132-7
25. Walter MR, Veevers JJ, Calver CR *et al.* Dating the 840–544 Ma Neoproterozoic interval by isotopes of strontium, carbon, and sulfur in seawater, and some interpretative models. *Precambrian Research*. 2000; **100**(1): 371-433. doi: https://doi.org/10.1016/S0301-9268(99)00082-0
26. Bergmann KD, Zentmyer RA, Fischer WW. The stratigraphic expression of a large negative carbon isotope excursion from the Ediacaran Johnnie Formation, Death Valley. *Precambrian Research*. 2011; **188**(1-4): 45-56. doi: 10.1016/j.precamres.2011.03.014
27. Corsetti FA, J. Kaufman A. Stratigraphic investigations of carbon isotope anomalies and Neoproterozoic ice ages in Death Valley, California. *Geological Society of America Bulletin*. 2003; **115**(8): 916-932. doi: 10.1130/b25066.1
28. Kaufman AJ, Corsetti FA, Varni MA. The effect of rising atmospheric oxygen on carbon and sulfur isotope anomalies in the Neoproterozoic Johnnie Formation, Death Valley, USA. *Chemical Geology*. 2007; **237**(1-2): 47-63. doi: 10.1016/j.chemgeo.2006.06.023
29. Jiang G, Christie-Blick N, Kaufman AJ *et al.* Carbonate platform growth and cyclicity at a terminal Proterozoic passive margin, Infra Krol Formation and Krol Group, Lesser Himalaya, India. *Sedimentology*. 2003; **50**(5): 921-952. doi: 10.1046/j.1365-3091.2003.00589.x
30. Kaufman AJ, Jiang G, Christie-Blick N *et al.* Stable isotope record of the terminal Neoproterozoic Krol platform in the Lesser Himalayas of northern India. *Precambrian Research*. 2006; **147**(1): 156-185. doi: https://doi.org/10.1016/j.precamres.2006.02.007
31. Moynihan DP, Strauss JV, Nelson LL *et al.* Upper Windermere Supergroup and the transition from rifting to continent-margin sedimentation, Nadaleen River area, northern Canadian Cordillera. *GSA Bulletin*. 2019; **131**(9-10): 1673-1701. doi: 10.1130/B32039.1
32. Macdonald FA, Schmitz MD, Crowley JL *et al.* Calibrating the Cryogenian. *Science*. 2009; **327**: 1241.
33. Prave AR, Fallick AE, Thomas CW *et al.* A composite C-isotope profile for the

Neoproterozoic Dalradian Supergroup of Scotland and Ireland. *Journal of the Geological Society*. 2009; **166**(5): 845-857. doi: 10.1144/0016-76492008-131

34. Macdonald FA, Jones DS, Schrag DP. Stratigraphic and tectonic implications of a newly discovered glacial diamictite-cap carbonate couplet in southwestern Mongolia. *Geology*. 2009; **37**(2): 123-126. doi: 10.1130/g24797a.1

35. Jiang G, Kaufman AJ, Christie-Blick N *et al.* Carbon isotope variability across the Ediacaran Yangtze platform in South China: Implications for a large surface-to-deep ocean δ13C gradient. *Earth and Planetary Science Letters*. 2007; **261**(1-2): 303-320.

36. Tahata M, Ueno Y, Ishikawa T *et al.* Carbon and oxygen isotope chemostratigraphies of the Yangtze platform, South China: Decoding temperature and environmental changes through the Ediacaran. *Gondwana Research*. 2013; **23**(1): 333-353.

37. Le Guerroué E, Allen PA, Cozzi A. Chemostratigraphic and sedimentological framework of the largest negative carbon isotopic excursion in Earth history: The Neoproterozoic Shuram Formation (Nafun Group, Oman). *Precambrian Research*. 2006; **146**(1-2): 68-92. doi: 10.1016/j.precamres.2006.01.007

38. Swanson-Hysell NL, Rose CV, Calmet CC *et al.* Cryogenian Glaciation and the Onset of Carbon-Isotope Decoupling. *Science*. 2010; **328**(5978): 608-611.

39. Cui H, Kaufman AJ, Xiao S *et al.* Redox architecture of an Ediacaran ocean margin: Integrated chemostratigraphic (δ13C–δ34S–87Sr/86Sr–Ce/Ce*) correlation of the Doushantuo Formation, South China. *Chemical Geology*. 2015; **405**: 48-62. doi: http://dx.doi.org/10.1016/j.chemgeo.2015.04.009

40. Kirschvink JL, Ripperdan RL, Evans DAD. Evidence for a large-scale reorganization of early Cambrian continental masses by inertial interchange true polar wander. *Science*. 1997; **277**: 541.

41. Evans DAD. True polar wander and supercontinents. *Tectonophysics*. 2003; **362**(1): 303-320. doi: https://doi.org/10.1016/S0040-1951(02)000642-X

42. Wen B, Evans DAD, Anderson RP *et al.* Late Ediacaran paleogeography of Avalonia and the Cambrian assembly of West Gondwana. *Earth and Planetary Science Letters*. 2020; **552**: 116591.

43. Robert B, Besse J, Blein O *et al.* Constraints on the Ediacaran inertial interchange true polar wander hypothesis: A new paleomagnetic study in Morocco (West African Craton). *Precambrian Research*. 2017; **295**: 90-116.

44. Robert B, Greff-Lefftz M, Besse J. True Polar Wander: A Key Indicator for Plate Configuration and Mantle Convection During the Late Neoproterozoic. *Geochemistry, Geophysics, Geosystems*. 2018; **19**(9): 3478-3495.

45. Wen B, Luo C, Li Y *et al.* Late Ediacaran inertial-interchange true polar wander (IITPW) event: a new road to reconcile the enigmatic paleogeography prior to the final assembly of Gondwana. *Turkish Journal of Earth Sciences*. 2022; **31**.

46. Escayola MP, van Staal CR, Davis WJ. The age and tectonic setting of the Puncoviscana Formation in northwestern Argentina: An accretionary complex related to Early Cambrian closure of the Puncoviscana Ocean and accretion of the Arequipa-Antofalla block. *Journal of South American Earth Sciences*. 2011; **32**(4): 438-459. doi: https://doi.org/10.1016/j.jsames.2011.04.013

47. Vizan H, Carney JN, Turner P *et al.* Late Neoproterozoic to Early Palaeozoic palaeogeography of Avalonia: some palaeomagnetic constraints from Nuneaton, central England. *Geological Magazine*. 2003; **140**(6): 685-705. doi: 10.1017/S001675680300832X

48. Schmitt RdS, Fragoso RdA, Collins AS. Suturing Gondwana in the Cambrian: The Orogenic

Events of the Final Amalgamation. In: Siegesmund S, Basei MAS, Oyhantçabal P, et al. (eds.). *Geology of Southwest Gondwana*. Cham: Springer International Publishing; 2018. 411-432.

49. Ganade de Araujo CE, Rubatto D, Hermann J *et al.* Ediacaran 2,500-km-long synchronous deep continental subduction in the West Gondwana Orogen. *Nature Communications*. 2014; **5**(1): 5198. doi: 10.1038/ncomms6198

50. Cordani UG, Pimentel MM, Ganade De Araújo CE *et al.* Was there an Ediacaran Clymene Ocean in central South America? *American Journal of Science*. 2013; **313**(6): 517. doi: 10.2475/06.2013.01

51. John T, Schenk V, Mezger K *et al.* Timing and PT Evolution of Whiteschist Metamorphism in the Lufilian Arc–Zambezi Belt Orogen (Zambia): Implications for the Assembly of Gondwana. *The Journal of Geology*. 2004; **112**(1): 71-90. doi: 10.1086/379693

52. Merdith AS, Collins AS, Williams SE *et al.* A full-plate global reconstruction of the Neoproterozoic. *Gondwana Research*. 2017; **50**: 84-134. doi: https://doi.org/10.1016/j.gr.2017.04.001

53. Boger SD. Antarctica — Before and after Gondwana. *Gondwana Research*. 2011; **19**(2): 335-371. doi: https://doi.org/10.1016/j.gr.2010.09.003

54. Meert JG, van der Voo R, Ayub S. Paleomagnetic investigation of the Neoproterozoic Gagwe lavas and Mbozi complex, Tanzania and the assembly of Gondwana. *Precambrian Research*. 1995; **74**(4): 225-244. doi: https://doi.org/10.1016/0301-9268(95)00012-T

55. Li ZX, Bogdanova SV, Collins AS *et al.* Assembly, configuration, and break-up history of Rodinia: a synthesis. *Precambrian Research*. 2008; **160**: 179.

56. Abrajevitch A, Van der Voo R. Incompatible Ediacaran paleomagnetic directions suggest an equatorial geomagnetic dipole hypothesis. *Earth and Planetary Science Letters*. 2010; **293**(1): 164-170. doi: https://doi.org/10.1016/j.epsl.2010.02.038

57. Jing X, Yang Z, Tong Y *et al.* Identification of multiple magnetizations of the Ediacaran strata in South China. *Geophysical Journal International*. 2018; **212**(1): 54-75. doi: 10.1093/gji/ggx396

58. Zhu B, Becker H, Jiang S-Y *et al.* Re–Os geochronology of black shales from the Neoproterozoic Doushantuo Formation, Yangtze platform, South China. *Precambrian Research*. 2013; **225**(0): 67-76. doi: http://dx.doi.org/10.1016/j.precamres.2012.02.002

59. Yao W-H, Li Z-X, Li W-X *et al.* From Rodinia to Gondwanaland: A tale of detrital zircon provenance analyses from the southern Nanhua Basin, South China. *American Journal of Science*. 2014; **314**(1): 278. doi: 10.2475/01.2014.08

60. Zhao G, Wang Y, Huang B *et al.* Geological reconstructions of the East Asian blocks: From the breakup of Rodinia to the assembly of Pangea. *Earth-Science Reviews*. 2018; **186**: 262-286. doi: https://doi.org/10.1016/j.earscirev.2018.10.003

61. Zhao H, Zhang S, Zhu M *et al.* Paleomagnetic insights into the Cambrian biogeographic conundrum: Did the North China craton link Laurentia and East Gondwana? *Geology*. 2020; **49**(4): 372-376. doi: 10.1130/g47932.1

62. Zhao H, Zhang S, Ding J *et al.* New geochronologic and paleomagnetic results from early Neoproterozoic mafic sills and late Mesoproterozoic to early Neoproterozoic successions in the eastern North China Craton, and implications for the reconstruction of Rodinia. *GSA Bulletin*. 2019; **132**(3-4): 739-766. doi: 10.1130/b35198.1

63. Ding J, Zhang S, Evans DAD *et al.* North China craton: The conjugate margin for northwestern Laurentia in Rodinia. *Geology*. 2021. doi: 10.1130/g48483.1

64. Zhou C, Yuan X, Xiao S *et al.* Ediacaran integrative stratigraphy and timescale of China. *Sci China Earth Sci*. 2019; **62**(1): 7-24.
65. Pang K, Wu C, Sun Y *et al.* New Ediacara-type fossils and late Ediacaran stratigraphy from the northern Qaidam Basin (China): Paleogeographic implications. *Geology*. 2021.
66. Xu X, Song S, Su L *et al.* The 600–580Ma continental rift basalts in North Qilian Shan, northwest China: Links between the Qilian-Qaidam block and SE Australia, and the reconstruction of East Gondwana. *Precambrian Research*. 2015; **257**: 47-64. doi: https://doi.org/10.1016/j.precamres.2014.11.017
67. Zhao P, Chen Y, Zhan S *et al.* The Apparent Polar Wander Path of the Tarim block (NW China) since the Neoproterozoic and its implications for a long-term Tarim–Australia connection. *Precambrian Research*. 2014; **242**: 39-57. doi: 10.1016/j.precamres.2013.12.009
68. Han Y, Zhao G, Cawood PA *et al.* Tarim and North China cratons linked to northern Gondwana through switching accretionary tectonics and collisional orogenesis. *Geology*. 2016; **44**(2): 95-98. doi: 10.1130/g37399.1
69. Wen B, Evans DAD, Li Y-X. Neoproterozoic paleogeography of the Tarim Block: An extended or alternative "missing-link" model for Rodinia? *Earth and Planetary Science Letters*. 2017; **458**: 92-106. doi: https://doi.org/10.1016/j.epsl.2016.10.030
70. Huang B, Piper JDA, Sun L *et al.* New paleomagnetic results for Ordovician and Silurian rocks of the Tarim Block, Northwest China and their paleogeographic implications. *Tectonophysics*. 2019; **755**: 91-108. doi: https://doi.org/10.1016/j.tecto.2019.02.010
71. Wen B, Evans DAD, Wang C *et al.* A positive test for the Greater Tarim Block at the heart of Rodinia: Mega-dextral suturing of supercontinent assembly. *Geology*. 2018; **46**(8): 687-690. doi: 10.1130/g40254.1
72. Shen B, Xiao S, Dong L *et al.* Problematic macrofossils from Ediacaran successions in the North China and Chaidam blocks: implications for there evolutionary root and biostratigraphic significance. *Journal of Paleontology*. 2007; **81**: 1396-1411.
73. de Alvarenga CJS, Figueiredo MF, Babinski M *et al.* Glacial diamictites of Serra Azul Formation (Ediacaran, Paraguay belt): Evidence of the Gaskiers glacial event in Brazil. *Journal of South American Earth Sciences*. 2007; **23**(2-3): 236-241. doi: 10.1016/j.jsames.2006.09.015
74. McGee B, Collins AS, Trindade RIF. A Glacially Incised Canyon in Brazil: Further Evidence for Mid-Ediacaran Glaciation? *The Journal of Geology*. 2013; **121**(3): 275-287.
75. Babinski M, Trindade RIF, Alvarenga CJS *et al.* Chronology of Neoproterozoic ice ages in central Brazil. *Am Symp Isot Geol, 5th (Punta del Este, Uruguay, 2006)*. 2006: 223-226.
76. McGee B, Collins AS, Trindade RIF *et al.* Investigating mid-Ediacaran glaciation and final Gondwana amalgamation using coupled sedimentology and 40Ar/ 39Ar detrital muscovite provenance from the Paraguay Belt, Brazil. *Sedimentology*. 2015; **62**(1): 130-154. doi: https://doi.org/10.1111/sed.12143
77. McGee B, Collins AS, Trindade RIF *et al.* Age and provenance of the Cryogenian to Cambrian passive margin to foreland basin sequence of the northern Paraguay Belt, Brazil. *GSA Bulletin*. 2015; **127**(1-2): 76-86. doi: 10.1130/b30842.1
78. Miller N, Johnson P, Stern B. Marine versus non-marine environments for the Jibalah Group, NW Arabian shield: A sedimentologic and geochemical survey and report of possible metazoa in the Dhaiqa formation. *Arabian Journal for Science and Engineering*. 2008; **33**: 55-77.
79. Fedonkin MA, Gehling JG, Grey K *et al.* *The Rise of Animals: Evolution and Diversification of*

the Kingdom Animalia. Baltimore: Jouns Hopkins University Press, 2007.

80. Corkeron ML. Chapter 65 Neoproterozoic glacial deposits of the Kimberly Region and northwestern Northern Territory, Australia. *Geological Society, London, Memoirs*. 2011; **36**(1): 659.

81. Corkeron ML, George AD. Glacial incursion on a Neoproterozoic carbonate platform in the Kimberley region, Australia. *Geological Society of America Bulletin*. 2001; **113**(9): 1121-1132.

82. Corkeron ML. 'Cap carbonates' and Neoproterozoic glacigenic successions from the Kimberley region, north-west Australia *Sedimentology*. 2007; **54**: 871-903.

83. Counts JW, Amos KJ. Sedimentology, depositional environments and significance of an Ediacaran salt-withdrawal minibasin, Billy Springs Formation, Flinders Ranges, South Australia. *Sedimentology*. 2016; **63**(5): 1084-1123. doi: https://doi.org/10.1111/sed.12250

84. Jenkins RJ. Billy Springs Glaciation, South Australia. In: E. Arnaud, Halverson GP, Sheilds-Zhou G (eds.). *The Geological Record of Neoproterozoic Glaciations*. London: Geol. Soc. London Mem.; 2011. 693-699.

85. Young T. The Bunyeroo Formation and its possible cold-water marine setting *BSc (Honours) thesis*. University of Adelaide, University of Adelaide, 1995.

86. Gostin VA, McKirdy DM, Webster LJ *et al.* Ediacaran ice-rafting and coeval asteroid impact, South Australia: insights into the terminal Proterozoic environment. *Australian Journal of Earth Sciences*. 2010; **57**(7): 859-869. doi: 10.1080/08120099.2010.509408

87. Webb AW, Coats RP, Fanning CM *et al.* Geochronological framework of the Adelaide Geosyncline. *Geological Society of Australia Abstracts*. 1983; **10**: 7-9.

88. Ireland TR, Flo¨ttmann T, Fanning CM *et al.* Development of the early Paleozoic Pacific margin of Gondwana from detrital-zircon ages across the Delamerian orogen. *Geology*. 1998; **26**(3): 243-246. doi: 10.1130/0091-7613(1998)026<0243:DOTEPP>2.3.CO;2

89. Martin MW, Grazhdankin DV, Bowring SA *et al.* Age of Neoproterozoic bilaterian body and trace fossils, White Sea, Russia: Implications for metazoan evolution. *Science*. 2000; **288**: 841-845.

90. Calver CR, Black LP, Everard JL *et al.* U-Pb zircon age constraints on late Neoproterozoic glaciation in Tasmania. *Geology*. 2004; **32**(10). doi: 10.1130/g20713.1

91. Direen NG, Jago JB. The Cottons Breccia (Ediacaran) and its tectonostratigraphic context within the Grassy Group, King Island, Australia: A rift-related gravity slump deposit. *Precambrian Research*. 2008; **165**(1): 1-14. doi: https://doi.org/10.1016/j.precamres.2008.05.008

92. Eyles N, Eyles CH. Glacially-influenced deep-marine sedimentation of the Late Precambrian Gaskiers Formation, Newfoundland, Canada. *Sedimentology*. 1989; **36**(4): 601-620.

93. Myrow PM, Kaufman AJ. A newly discovered cap carbonate above Varanger-age glacial deposits in Newfoundland, Canada. *Journal of Sedimentary Research*. 1999; **69**: 784-793.

94. Pu J, Bowring SA, Ramezani J *et al.* Dodging snowballs: Geochronology of the Gaskiers glaciation and the first appearance of the Ediacaran biota. *Geology (Boulder)*. 2016; **44**(11): 955-958.

95. Kawai T, Windley BF, Terabayashi M *et al.* Geotectonic framework of the Blueschist Unit on Anglesey–Lleyn, UK, and its role in the development of a Neoproterozoic accretionary orogen. *Precambrian Research*. 2007; **153**(1): 11-28. doi: https://doi.org/10.1016/j.precamres.2006.11.002

96. Kawai T, Windley BF, Terabayashi M *et al.* Neoproterozoic glaciation in the mid-oceanic realm: An example from hemi-pelagic mudstones on Llanddwyn Island, Anglesey, UK.

Gondwana Research. 2008; **14**(1): 105-114.
97. Sayles RW. The Squantum Tillite. *Bulletin of the Museum of Comparative Zoology at Harvard College*. 1914; **56**: 141-175.
98. Carto SL, Eyles N. Sedimentology of the Neoproterozoic (c. 580Ma) Squantum 'Tillite', Boston Basin, USA: Mass flow deposition in a deep-water arc basin lacking direct glacial influence. *Sedimentary Geology*. 2012; **269-270**: 1-14. doi: 10.1016/j.sedgeo.2012.03.011
99. Thompson MD, Bowring SA. Age of the Squantum 'tillite', Boston basin, Massachusetts: U-Pb zircon constraints on terminal Neoproterozoic glaciation. *American Journal of Science*. 2000; **300**: 630-655.
100. Edwards MB. Sedimentology of the Upper Proterozoic Glacial Record, Vestertana Group, Finnmark, North Norway. *Norges Geologiske Undersøkelse Bulletin*. 1984; **394**: 76.
101. Farmer J, Vidal G, Moczydłowska M *et al.* Ediacaran fossils from the Innerelv Member (late Proterozoic) of the Tanafjorden area, northeastern Finnmark. *Geological Magazine*. 1992; **129**(2): 181-195. doi: 10.1017/S001675680000827X
102. Chumakov NM. Climates and climate zonality of the Vendian: geological evidence. *Geological Society, London, Special Publications*. 2007; **286**(1): 15-26. doi: 10.1144/sp286.2
103. Rice AHN, Edwards, M.B., Hansen, T., Arnaud, E., & Halverson, G. P. Glacigenic rocks of the Smalfjord and Mortensnes Formations, Vestertana Group, E. Finnmark, Norway. *Geological Society of London*. 2011; **Memoirs 36**: 593-602.
104. Gorokhov IM, Siedlecka A, Roberts D *et al.* Rb–Sr dating of diagenetic illite in Neoproterozoic shales, Varanger Peninsula, northern Norway. *Geological Magazine*. 2001; **138**(5): 541-562. doi: 10.1017/S001675680100574X
105. Bingen B, Griffin WL, Torsvik TH *et al.* Timing of Late Neoproterozoic glaciation on Baltica constrained by detrital zircon geochronology in the Hedmark Group, south-east Norway. *Terra Nova*. 2005; **17**(3): 250-258. doi: https://doi.org/10.1111/j.1365-3121.2005.00609.x
106. Chumakov NM. Trends in Global Climate Changes Inferred from Geological Data. *Stratigraphy and Geological Correlation*. 2004; **12**.
107. Chumakov NM. The key section of Vendian glacial deposits in the South Urals (Kurgashly Formation, Krivoluksky graben). In: Knipper AL, Kurenkov CA, Semikhatov MA (eds.). *The Urals: Fundamental Problems of Geodynamics and Stratigraphy*. Moscow: Nauka; 1998. 1398153.
108. Maslov AV, Meert J, Levashova NM *et al.* New constraints for the age of Vendian glacial deposits (Central Urals). *Doklady Earth Sciences*. 2013; **449**(1): 303-308.
109. Chumakov NM. Chapter 24 The Neoproterozoic glacial formations of the North and Middle Urals. *Geological Society, London, Memoirs*. 2011; **36**(1): 289. doi: 10.1144/M36.23
110. Grazhdankin DV, Marusin VV, Meert J *et al.* Kotlin regional stage in the South Urals. *Doklady Earth Sciences*. 2011; **440**(1): 1222. doi: 10.1134/S1028334X11090170
111. Linnemann U, Pidal AP, Hofmann M *et al.* A ~565 Ma old glaciation in the Ediacaran of peri-Gondwanan West Africa. *International Journal of Earth Sciences*. 2018; **107**(3): 885-911.
112. Shen B, Xiao S, Zhou C *et al.* Carbon and sulfur isotope chemostratigraphy of the Neoproterozoic Quanji Group of the Chaidam Basin, NW China: Basin stratification in the aftermath of an Ediacaran glaciation postdating the Shuram event? *Precambrian Research*. 2010; **177**(3): 241-252. doi: https://doi.org/10.1016/j.precamres.2009.12.006
113. Wang R, Xing C, Wen B *et al.* The origin of Ediacaran glacial cap carbonate (in press). 2023.
114. Grotzinger JP, Bowring SA, Saylor BZ *et al.* Biostratigraphic and geochronologic constraints

on early animal evolution. *Science*. 1995; **270**: 598-604.

115. Germs GJB, Gaucher C. Nature and extent of a late Ediacaran (ca. 547Ma) glacigenic erosion surface in southearn Africa. *South African Journal of Geology*. 2012; **115**(1): 91-102.

116. Hofmann M, Linnemann U, Hoffmann K-H *et al.* The four Neoproterozoic glaciations of southern Namibia and their detrital zircon record: The fingerprints of four crustal growth events during two supercontinent cycles. *Precambrian Research*. 2015; **259**: 176-188. doi: https://doi.org/10.1016/j.precamres.2014.07.021

117. Etemad-Saeed N, Hosseini-Barzi M, Adabi MH *et al.* Evidence for ca. 560Ma Ediacaran glaciation in the Kahar Formation, central Alborz Mountains, northern Iran. *Gondwana Research*. 2016; **31**: 164-183.

118. Frimmel HE, Fölling PG, Eriksson PG. Neoproterozoic tectonic and climatic evolution recorded in the Gariep Belt, Namibia and South Africa. 2002; **14**(1): 55-67. doi: https://doi.org/10.1046/j.1365-2117.2002.00166.x

119. Fölling PG, Frimmel HE. Chemostratigraphic correlation of carbonate successions in the Gariep and Saldania Belts, Namibia and South Africa. *Basin Research*. 2002; **14**(1): 69-88. doi: https://doi.org/10.1046/j.1365-2117.2002.00167.x

120. Gaucher C, A.N. S, Halverson GP *et al.* The Neoproterozoic and Cambrian: a time of upheavals, extremes and innovations. In: Gaucher C, Sial AN, Halverson GP, et al. (eds.). *Neoproterozoic-Cambrian Tectonics, Global Change and Evolution: a focus on southwestern Gondwana*. Netherlands: Developments in Precambrian Geology; 2009. 3-11.

121. Southworth S, Tollo RP, Aleinikoff JN *et al.* New geologic map and geochronology of the Shenandoah National Park region, Virginia. *Geological Society of America Abstracts with Programs*. 2009; **41**: 365.

122. Hebert CL, Kaufman AJ, Penniston-Dorland SC *et al.* Radiometric and stratigraphic constraints on terminal Ediacaran (post-Gaskiers) glaciation and metazoan evolution. *Precambrian Research*. 2010; **182**(4): 402-412.

123. McCay GA, Prave AR, Alsop GI *et al.* Glacial trinity: Neoproterozoic Earth history within the British-Irish Caledonides. *Geology*. 2006; **34**(11). doi: 10.1130/g22694a.1

124. Dempster TJ, Rogers G, Tanner PWG *et al.* Timing of deposition, orogenesis and glaciation within the Dalradian rocks of Scotland: Constraints from U-Pb zircon ages. *Journal of the Geological Society, London*. 2002; **159**: 83-94.

125. Halliday AN, Graham CM, Aftalion M *et al.* The depositional age of the Dalradian Supergroup: U-Pb and Sm-Nd isotopic studies of the Tayvallich Volcanics, Scotland. *Journal of the Geological Society*. 1989; **146**(1): 3.

126. Guan B, Wu R, Hambrey MJ *et al.* Glacial sediments and erosional pavements near the Cambrian- Precambrian boundary in western Henan Province, China. *Journal of the Geological Society, London*. 1986; **143**: 311-323.

127. Yang J, Lyons TW, Zeng Z *et al.* Geochemical constraints on the origin of Neoproterozoic cap carbonate in the Helan Mountains, North China: Implications for mid-late Ediacaran glaciation? *Precambrian Research*. 2019; **331**. doi: 10.1016/j.precamres.2019.105361

128. Ruitang W, Baode G. Glacigenic Characteristics of the Luoquan Formation and Sediment Gravity Flow Reworking on It: Glacigenic Characteristics of Luoquan Formation. *Acta Geologica Sinica-english Edition - ACTA GEOL SIN-ENGL ED*. 2009; **1**: 325-339. doi: 10.1111/j.1755-6724.1988.mp1003007.x

129. Le Heron DP, Vandyk TM, Wu G *et al.* New perspectives on the Luoquan Glaciation (Ediacaran-Cambrian) of North China. *The Depositional Record*. 2018; **4**(2): 274-292.

130. Gaucher C, Blanco G, Chiglino L *et al.* Acritarchs of Las Ventanas Formation (Ediacaran, Uruguay): Implications for the timing of coeval rifting and glacial events in western Gondwana. *Gondwana Research*. 2008; **13**(4): 488-501. doi: https://doi.org/10.1016/j.gr.2007.05.008

131. Sanchez Bettucci L, Linares E. Primeras edades en Basaltos del Complejo Sierra de las Animas. *XIII Congreso Geológico Argentino y III Congreso de Exploración de Hidrocarburos, Actas*. 1996; **1**: 399-404.

132. Oyhantçabal P, Siegesmund S, Wemmer K *et al.* Age and geochemical signature of post-collisional plutons of the southern extreme of the Dom Feliciano Belt (Uruguay). *South American Symposium on Isotope Geology (Short Papers)*. 2006: 148.

133. Blanco G, Gaucher C. Estratigrafía, Paleontología y Edad de la Formación Las Ventanas (Neoproterozoico, Uruguay). *Latin American journal of sedimentology and basin analysis*. 2005; **12**: 109-124.

134. Perdoncini LC, Soares PC. Depósitos glaciogênicos do Proterozóico Superior no leste do Paraná. *Proceedings of Congresso Brasileiro de Geologia 37th*. 1992; **2**: 452-453.

135. Campanha GAC, Basei MS, Tassinari CCG *et al.* Constraining the age of the Iporanga Formation with SHRIMP U-Pb zircon: Implications for possible Ediacaran glaciation in the Ribeira Belt, SE Brazil. *Gondwana Research*. 2008; **13**(1): 117-125. doi: https://doi.org/10.1016/j.gr.2007.05.010

136. Osokin PV, Tyzhinov AV. Precambrian tilloids of the Oka-Khubsugul phosphorite-bearing basin (eastern Sayan, northwestern Mongolia). *Lithol Miner Resour*. 1998; **33**: 142-154.

137. Chumakov NM. Glacial deposits of the Bokson Group, East Sayan Mountains, Buryatian Republic, Russian Federation. *Geological Society, London, Memoirs*. 2011; **36**(1): 285. doi: 10.1144/M36.23

138. Chumakov NM. The Baykonurian glaciohorizon of the Late Vendian. *Stratigraphy and Geological Correlation*. 2009; **17**(4): 373-381.

139. Chumakov NM. Chapter 11.2 Neoproterozoic Glacial Events in Eurasia. In: Gaucher C, Sial AN, Frimmel HE, et al. (eds.). *Developments in Precambrian Geology*. Elsevier; 2009. 389-403.

140. Chumakov NM. Glacial deposits of the Baykonur Formation, Kazakhstan and Kyrgyzstan. In: Arnaud E, Halverson GP, Shields-Zhou G (eds.). *The Geological Record of Neoproterozoic Glaciations*: Geological Society of London; 2011. 0.

141. Wang F. Sinian microfossils from south-west China. *Nature*. 1981; **294**: 74-76.

142. Xiao S, Bao H, Wang H *et al.* The Neoproterozoic Quruqtagh Group in eastern Chinese Tianshan: evidence for a post-Marinoan glaciation. *Precambrian Research*. 2004; **130**(1-4): 1-26. doi: 10.1016/j.precamres.2003.10.013

143. Fedonkin MA, Simonetta A, Ivantsov AY. New data on *Kimberella*, the Vendian mollusc-like organism (White Sea region, Russia): palaeoecological and evolutionary implications. In: Vickers-Rich P, Komarower P (eds.). *The Rise and Fall of the Ediacaran Biota*: Geological Society of London Special Publications 286; 2007. 157-179.

144. Letsch D, Large SJE, Buechi MW *et al.* Ediacaran glaciations of the west African Craton – Evidence from Morocco. *Precambrian Research*. 2018; **310**: 17-38.

145. Inglis JD, D'Lemos RS, Samson SD *et al.* Geochronological constraints on late Precambrian intrusion, metamorphism, and tectonism in the Anti-Atlas Mountains. *J Geol*. 2005; **113**: 439-

450.

146. Vernhet E, Youbi N, Chellai EH *et al.* The Bou-Azzer glaciation: Evidence for an Ediacaran glaciation on the West African Craton (Anti-Atlas, Morocco). *Precambrian Research*. 2012; **196-197**: 106-112. doi: https://doi.org/10.1016/j.precamres.2011.11.009

147. Letsch D, Large SJE, Bernasconi SM *et al.* Northwest Africa's Ediacaran to early Cambrian fossil record, its oldest metazoans and age constraints for the basal Taroudant Group (Morocco). *Precambrian Research*. 2019; **320**: 438-453. doi: https://doi.org/10.1016/j.precamres.2018.11.016

148. Karaoui B, Breitkreuz C, Mahmoudi A *et al.* U–Pb zircon ages from volcanic and sedimentary rocks of the Ediacaran Bas Draâ inlier (Anti-Atlas Morocco): Chronostratigraphic and provenance implications. *Precambrian Research*. 2015; **263**: 43-58. doi: https://doi.org/10.1016/j.precamres.2015.03.003

149. Caby R, Fabre J. Late Proterozoic to Early Palaeozoic Diamictites/Tillites and Associated Glaciogenic Sediments in the Serie Pourpree of Western Hoggar, Algeria. In: Hambrey MJ, Harland WB (eds.). *Earth's Pre-Pleistocene Glacial Record*. Cambridge: Cambridge Univ. Press; 1981. 140-145.

150. Deynoux M, Affaton P, Trompette R *et al.* Pan–African tectonic evolution and glacial events registered in Neoproterozoic to Cambrian cratonic and foreland basins of West Africa. *Afr Earth Sci*. 2006; **46**: 397-426.

151. Bertrand-Sarfati J, Moussine-Pouchkine A, Amard B *et al.* First Ediacaran fauna found in western Africa and evidence for an Early Cambrian glaciation. *Geology*. 1995; **23**: 133-136.
